# Hydroxamic acid derivatives as HDAC1, HDAC6 and HDAC8 inhibitors with antiproliferative activity in cancer cell lines

**DOI:** 10.1038/s41598-020-67112-4

**Published:** 2020-06-26

**Authors:** Yudibeth Sixto-López, José Antonio Gómez-Vidal, Nuria de Pedro, Martiniano Bello, Martha Cecilia Rosales-Hernández, José Correa-Basurto

**Affiliations:** 1 Laboratorio de Diseño y Desarrollo de Nuevos Fármacos e Innovación Biotecnológica (Laboratory for the Design and Development of New Drugs and Biotechnological Innovation)-SEPI, Escuela Superior de Medicina, Instituto Politécnico Nacional, 11340 Mexico City, Mexico; 2 Departamento de Química Farmacéutica y Orgánica, Facultad de Farmacia, Universidad de Granada, 18071 Granada, Spain; 3 Fundación MEDINA, Centro de Excelencia en Investigación de Medicamentos Innovadores en Andalucía, 18016 Granada, Spain; 4 Laboratorio de Biofísica y Biocatálisis, Sección de Estudios de Posgrado e Investigación, Escuela Superior de Medicina, Instituto Politécnico Nacional, Ciudad de México, Mexico

**Keywords:** Cheminformatics, Pharmacology, Small molecules

## Abstract

Histone deacetylases (HDACs) belong to a family of enzymes that remove acetyl groups from the ɛ-amino of histone and nonhistone proteins. Additionally, HDACs participate in the genesis and development of cancer diseases as promising therapeutic targets to treat cancer. Therefore, in this work, we designed and evaluated a set of hydroxamic acid derivatives that contain a hydrophobic moiety as antiproliferative HDAC inhibitors. For the chemical structure design, in silico tools (molecular docking, molecular dynamic (MD) simulations, ADME/Tox properties were used to target Zn^2+^ atoms and HDAC hydrophobic cavities. The most promising compounds were assayed in different cancer cell lines, including hepatocellular carcinoma (HepG2), pancreatic cancer (MIA PaCa-2), breast cancer (MCF-7 and HCC1954), renal cancer (RCC4-VHL and RCC4-VA) and neuroblastoma (SH-SY5Y). Molecular docking and MD simulations coupled to the MMGBSA approach showed that the target compounds have affinity for HDAC1, HDAC6 and HDAC8. Of all the compounds evaluated, YSL-109 showed the best activity against hepatocellular carcinoma (HepG2 cell line, IC_50_ = 3.39 µM), breast cancer (MCF-7 cell line, IC_50_ = 3.41 µM; HCC1954 cell line, IC_50_ = 3.41 µM) and neuroblastoma (SH-SY5Y cell line, IC_50_ = 6.42 µM). *In vitro* inhibition assays of compound YSL-109 against the HDACs showed IC_50_ values of 259.439 µM for HDAC1, 0.537 nM for HDAC6 and 2.24 µM for HDAC8.

## Introduction

Histone deacetylases (HDACs) are a family of enzymes able to remove acetyl groups from lysine residues in histones and nonhistone proteins, which includes chaperone proteins and transcription factors, among others^1,2^; the opposite effect is exerted by histone acetyltransferases (HATs)^3^. The family of HDACs include 18 members classified into four classes: class I (HDACs 1, 2, 3 and 8), class II subdivides into IIa (HDACs 4, 5, 7 and 9) and IIb (HDACs 6 and 10) and class IV (HDAC11), all of which are Zn^2+^ dependent, whereas class III, also called sirtuins, are NAD^+^-dependent enzymes^2^.

Due to the biological importance of HDACs, their inhibition has emerged as an important strategy to treat several diseases, including cancer^4–6^, neurodegenerative diseases^7,8^, cardiac diseases^9^, immune diseases^10^, inflammatory diseases^11^, and other disorders^12,13^.

It has been described that HDAC inhibition produces cell cycle arrest, the inhibition of tumor angiogenesis, differentiation of some transformed cell lines^14^ and/or apoptosis in tumor cells^4,5,15^, showing particular importance as a pharmacological target for cancer treatment. Currently on the market, there are five HDAC-related compounds approved for cancer treatment: vorinostat (suberoylanilide hydroxamic acid, SAHA), romidepsin, chidamide and Belinostat for T-cell lymphoma and panobinostat for the treatment of myeloma^16,17^.

HDAC1, HDAC6 and HDAC8 are isoforms belonging to different HDAC classes, and localize in different locations within the cell^2^. HDAC1 and HDAC8 are mainly localized in the nucleus^2^ while HDAC6 is mainly localized in the cytoplasm^18^. These HDACs have been linked to cancer progression and development^19^ and are also related to neurodegenerative diseases^7,18,20,21^. These differences suggest the importance in the development of isoform-selective HDAC inhibitors (HDACi) to enhance drug efficacy and decrease side effects^22,23^. HDAC inhibitors have common structural features that allow them to interact with HDAC enzymes by means of noncovalent interactions. The general pharmacophore of HDACi include (a) a zinc chelating group, (b) a linker group, which is generally hydrophobic to fit the catalytic site channel, and (c) a capping group, which interacts with the hydrophobic surface region and is the main factor responsible for isoform selectivity^24–26^. Therefore, structural insights into the binding differences among HDAC isoforms that permits the design of new HDACi with improved affinity and selectivity need to be explored. In this sense, several structural studies have been performed revealing particular differences among HDAC isoforms. In the case of HDAC8, experimental and theoretical studies have revealed the presence of several possible binding sites, including the catalytic site (CS)^27,28^, an alternate site of entry adjacent to the catalytic site that reaches the CS and consists of a 14 Å so-called tunnel adjacent to the catalytic site pocket (ACSP)^27,29^, and this a 14 Å tunnel^26,30^ and the acetyl-release channel (HSAC)^31,32^ are mostly characterized by their hydrophobic nature. In addition, selective HDAC1 inhibitors can be obtained by exploiting the foot pocket (14 Å channel), which is observed in class I HDACs but not in class IIa^33^, which is important for the correct catalytic activity of the enzyme^34^. On the other hand, molecular modeling studies indicate that DD2-HDAC6 (the catalytic domain 2 of HDAC6) has structural differences in the cap region, which is more flexible in comparison to the class I isoforms^22,35–37^. It also possesses a tunnel that is wider and more shallow^7,37^, structural data supported by X-ray diffraction studies placed at Protein Data Base (PDB): 5EDU (Human), 5EEF, 5EEI, 5EEK, 5EEM, 5EEN, 5EF7, 5EF8, 5EF8, 5EFG 5EFH, 5EFJ, 5EFK and 5EFN (Danio), and therefore, it has been suggested that the use of bulky and aromatic moieties would increase the selectivity for HDAC6^18,26^.

Therefore, we designed a new series of compounds that contain two pharmacophore moieties (hydroxamic and aromatic) to target HDAC1, HDAC6 and HDAC8 to be assayed as antiproliferative compounds on cancer cell lines (HepG2, MCF-7, SH-SY5Y, MIA PaCa-2, HCC1954, RCC4-VA, RCC4-VHL), exploring the binding pose using molecular docking and molecular dynamics (MD) simulations on HDAC1, HDAC6 and HDAC8 and further validation with *in vitro* assays.

## Results and Discussion

**Scheme 1:**
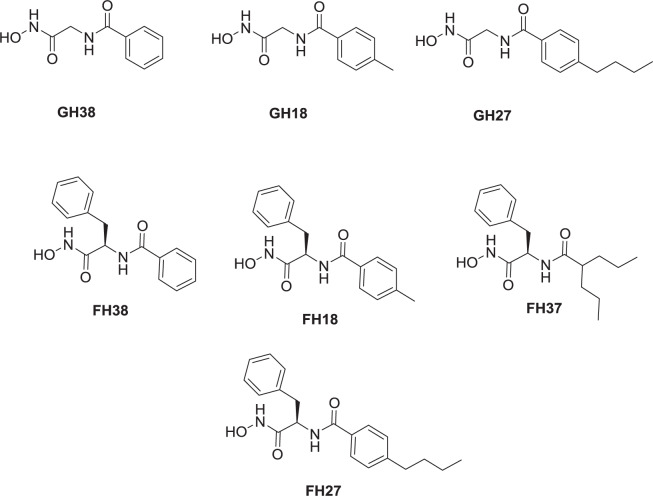
First generation of hydroxamic acid derivatives, including the lead compound FH27.

HDACs deacetylate histone and nonhistone proteins^2^, and an imbalance in their function or expression is involved in diverse disease states, such as cancer^4–6^ and neurodegenerative diseases^7^, among others^9–12^. HDACs are one of the most promising therapeutic targets for the treatment of cancer and have been widely studied^15,19,20,38,39^. First, a set of 7 hydroxamic acid derivatives were developed (GH38, GH18, GH27, FH38, FH18, FH27 and FH37, Scheme 1), and FH27 was identified as a lead compound, through cytotoxic evaluation on HepG2, MCF-7 and MIA PaCa-2, where 50 µM of the above compound were tested in order to obtain the % of inhibition in the cell viability (Table 1), besides from a previous work, it was found by blind molecular docking that FH27 did not reached the catalytic site of HDAC1 and HDAC8, reaching HDAC6 over the other two isoforms (paper not published yet) taken into account the *in silico* approach and *in cytotoxicity*, this compound was used to develop a second series of compounds to target HDAC1, 6 and 8.

**Table 1:** Results of the cytotoxic evaluation in HepG2, MCF-7 and MIA PaCa-2 cell lines as shown as the % inhibition at 50 µM.

Compound	HepG2		MCF-7		MIA PaCa-2	
	IC_50_	% Inhibition at 50 µM	IC_50_	% Inhibition at 50 µM	IC_50_	% Inhibition at 50 µM
**GH38**	>50	1.01	>50	16.10	>50	0.18
**GH18**	>50	3.08	>50	17.22	>50	4.44
**GH27**	>50	1.38	>50	40.40	>50	10.72
**FH38**	>50	14.07	>50	20.98	>50	0.43
**FH18**	>50	5.52	>50	41.24	>50	0.05
**FH27**	>50	35.36	>50	37.59	>50	2.82
**FH37**	>50	48.56	>50	49.11	>50	7.47

### Lead compound optimization

**Figure 1:**
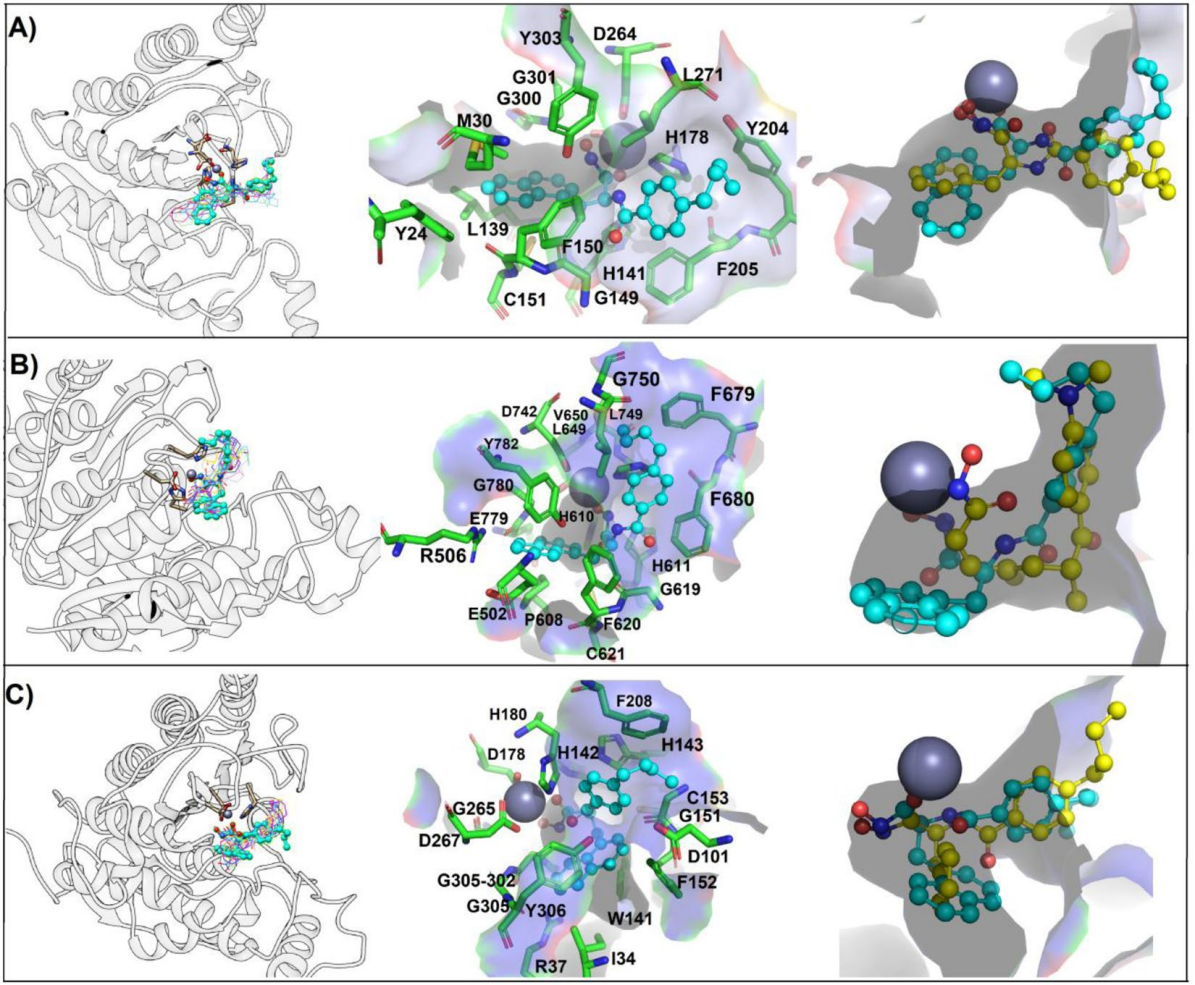
Binding mode of studied compounds retrieved from focused molecular docking. (**A**) HDAC1, (**B**) HDAC6 and (**C**) HDAC8. From left panel HDAC is in white ribbon representation, FH27, YSL99, YSL106, YSL112, YSL116, YSL121, YSL125 and YSL129 are depicted as wire, while YSL109 is depicted as cyan ball and stick, in the middle panel a zoom on the YSL109 and residues with which it interacts are depicted, YSL109 is depicted as cyan ball and stick, while interacting residues as green sticks; and finally in the right panel a surface representation of the catalytic tunnel is depicted in gray, YSL109 is depicted in cyan, while FH27 is depicted in yellow ball and stick, Zn is depicted as gray sphere. Figure built with Pymol and UCSF Chimera softwares.

**Scheme 2:**
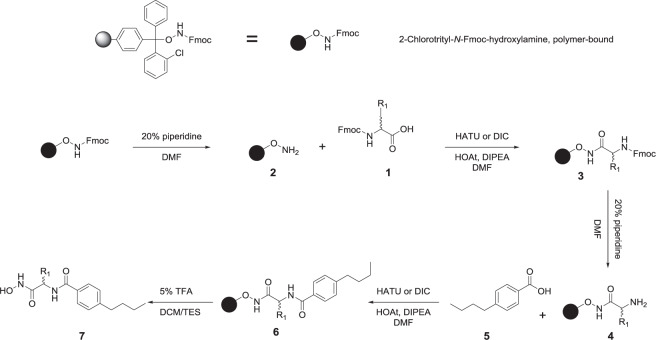
Solid-phase synthesis of hydroxamic acid derivatives.

Molecular modeling studies showed that FH27 reaches the catalytic site and is coordinated with Zn in bidentate way (2.887 and 2.677 Å) (Fig. 1B), and interacts with residues belonging to the internal cavity, leaving free the cap region of DD2-HDAC6; thus, there were opportunities to include voluminous groups such as the bulky naphthyl, electron donating (-OH) or electron withdrawing (–F, –I, –NO_2_) groups in the cap region composed of a phenyl group^7,35,36,40^. Additionally, with the aim of exploring whether the stereochemical properties affect the biological effects, the *R* form of the lead compound (YSL-106) was synthesized. As a result, eight compounds were synthesized (Table 2 following a solid-phase synthesis protocol (Scheme 2).

**Table 2:** Antiproliferative activities of the second series of compounds against different cancer cell lines.

Second Generation		IC_50_ (µM)						
Name	R_1_	HepG2	MIA PaCa-2	MCF-7	HCC1954	RCC4-VA	RCC4-VHL	SH-SY5Y

YSL-99		30.88	>50	8.96	42.90	>50	>50	36.19
YSL-106		11.55	21.55	4.94	26.10	12.92	21.10	18.91
YSL-109		3.39	>50	3.41	3.77	24.87	32.69	6.42
YSL-112		10.85	>50	7.27	7.62	31.87	46.50	21.44
YSL-116		17.98	>50	27.88	17.97	38.87	44.17	19.62
YSL-121		41.14	>50	>50	8.49	>50	>50	1.41
YSL-125		15.69	42.52	16.64	14.77	10.46	8.02	6.50
YSL-129		>50	>50	>50	>50	>50	>50	>50

### Molecular docking

Molecular docking procedures were used to generate the starting coordinates of the HDAC ligands to perform the MD simulation studies. The lead compound and derivatives showed a common binding mode within HDAC1 (Fig. 1A). The hydroxamic group of the ligands reached the bottom of the hydrophobic tunnel and were coordinated with the Zn^2+^ through hydroxamic group, except YS-106, that is slightly displaced toward the internal cavity of HDAC1 and interacted with Zn^2+^ through the oxygen of the amide function. The phenyl moiety of the compounds is inserted into the 14 Å tunnel and exposed its aliphatic portion toward the surface region. YSL-109 and YSL-112 fit better into the 14 Å tunnel because their naphthyl portion was accommodated better along the tunnel and, at the same time, the hydroxyl group of the hydroxamic acid moiety interacted with Zn^2+^ (Fig. 1A).

All the tested ligands recognize and make similar binding mode on HDAC6 and were coordinated with Zn^2+^(Fig. 1B), except for YSL-121 (See Supplementary information, Figs. S1 and S2). All compounds inserted the naphthyl portion toward the 14 Å, except YSL-112; instead, it occupied the hydrophobic tunnel together with the linker (aromatic and aliphatic) region. YSL-121 was accommodated into the hydrophobic tunnel in a reverse manner, where the aliphatic chain was oriented toward the Zn^2+^ or the hydrophobic portion was placed in the surface region (See Supplementary information, Fig. S2). Finally, the remaining ligands repeated the same binding mode as that observed for the other isoforms (hydroxamic acid toward the Zn^2+^ atom), except YSL-106, which did not interact with Zn^2+^ even when it was placed at the end of the catalytic tunnel (Fig. 1B). Trichostatin A (TSA), which has an aromatic and a hydroxamic groups reproduces the typical binding mode reported for HDACs^28,41,42^.

While for HDAC8, all compound except YSL-106, share a similar binding mode, since they were coordinated with Zn^2+^ in a monodentate way through the hydroxyl portion of the hydroxamic group, while the phenyl and naphthyl rings were inserted into the 14 Å tunnel and the aliphatic chain protrude to the outer of the catalytic tunnel, in case of YSL-106, the only difference regarding the other molecules was that it did not coordinate the hydroxamic portion with Zn^2+^ (Fig. 1C) (Supplementary information, Figs. S1 and S2).

### Antiproliferative activity

Furthermore, we evaluated the antiproliferative effect of the synthesized second series of compounds in different cancer cell lines. As shown in Table 2, addition of the hydroxyl group at the *para* position of YSL-99 did not show a significant effect on cytotoxicity; however, it showed cytotoxicity effects against the MCF-7 breast cancer cell line.

The *R* isomer of the lead compound (YSL-106) was synthesized to evaluate the stereoselectivity of HDAC inhibitors on biological activity since there have been previous reports where this influence has been described^43,44^. YSL-106 shows an enhanced cytotoxic effect compared to FH27. Further, studies need to be performed to evaluate the *in vitro* isoform selectivity of this compound to explain our biological findings, but it seems that YSL-106 shows improved biological activity, which might be at the expense of isoform selectivity as the MD simulations point out (see further details).

The addition of bulkier substituents was studied by adding α- and β-naphthyl residues, obtaining YSL-109 and YSL-112, respectively. With regard to their cytotoxic activity, YSL-109 in most cases showed better activity than YSL-112.

YSL-129 did not show biological activity, which could be due to the elimination of the β-carbon, suggesting that the presence of the β-carbon is important for the biological activity of these compounds.

Fluorine and iodine were added to YSL-116 and YSL-121, which are electron donating groups; moreover, the electron withdrawing nitro group was added to YSL-125. The inclusion of fluorine in the structure of YSL-116 detrimentally affected the antiproliferative effect, but this compound was still superior to FH27 (5.59 µM v*s* 13.70 µM ± 4.10). In contrast, YSL-121 showed more limited antiproliferative effects because these effects were only observed in hepatoma HepG2 cells (41.14 µM), breast cancer HCC1954 cells (8.49 µM), and neuroblastoma SH-SY5Y cells (1.41 µM), with the best activity being observed in the SH-SY5Y cells.

YSL-125 showed a better cytotoxic effect in the renal carcinoma RCC4-VA and RCC4-VHL cell lines, of which RCC4-VA shows overexpression of hypoxia-inducible-factor (HIF), which is a gene involved in the regulation of the expression of a large number of target genes involved in tumor progression^45^, including those regulated by oxygen^46^. On the other hand, RCC4-VHL shows a normal level of expression of HIF, which corresponds to the wild type cell line^46^. The cytotoxic effect of YSL-125 was similar in both cell lines; therefore, we can broadly suggest that this compound may have HDAC6 inhibitory activity based on the structural similarity with YSL-109 since HDAC6 in numerous studies has been shown to be involved in the degradation of HIF^47,48^ through a VHL-independent way^49^.

### Molecular dynamics simulations

Once the biological activities of the compounds were determined, MD simulations of the complexes (compound-HDAC) of the nine compounds with biological activity were carried out to predict the affinity for HDAC1, HDAC6 and HDAC8. HDAC1 shares sequence homology with HDAC2 and HDAC3 and is generally inhibited with almost the same strength by inhibitors^50^, and so in this way, the MD simulations may encompass or have a notion about the possible behavior of the compounds with regard to class I HDACs.

First, the starting coordinates of the compounds were obtained for molecular docking using the improved forcefield Autodock4zn^51^. Since the criteria of selection was to take the coordinates of the ligand coordinated with the Zn^2+^ by its hydroxamic moiety, this objective was achieved by reducing the size of the grid box in all cases at the expense of increasing the value of the binding free of energy (Table S1), even allowing this value to become positive, such as was the case for FH27 and the compounds from the second series in complex with HDAC6, except for TSA (−6.11 kcal/mol). In the case of HDAC1, the binding free energies were thermodynamically favorable in the range of −40 to −11 kcal/mol. For HDAC8, the binding free energy values ranged from −32 to −6 kcal/mol, except for YSL-109 and YSL-112, which are naphthyl derivatives that showed positive values (Supplementary information, Table S1). The positive values obtained might indicate that this is not the most favorable conformer of the ligand; thus, favorable interactions were not established. However, with the use of this conformation as the starting structure for MD simulations, it was indicated that these conformations are stable if this binding mode was maintained, or they were deemed not stable if they changed their position or broke the complex during the MD simulation time.

**Figure 2:**
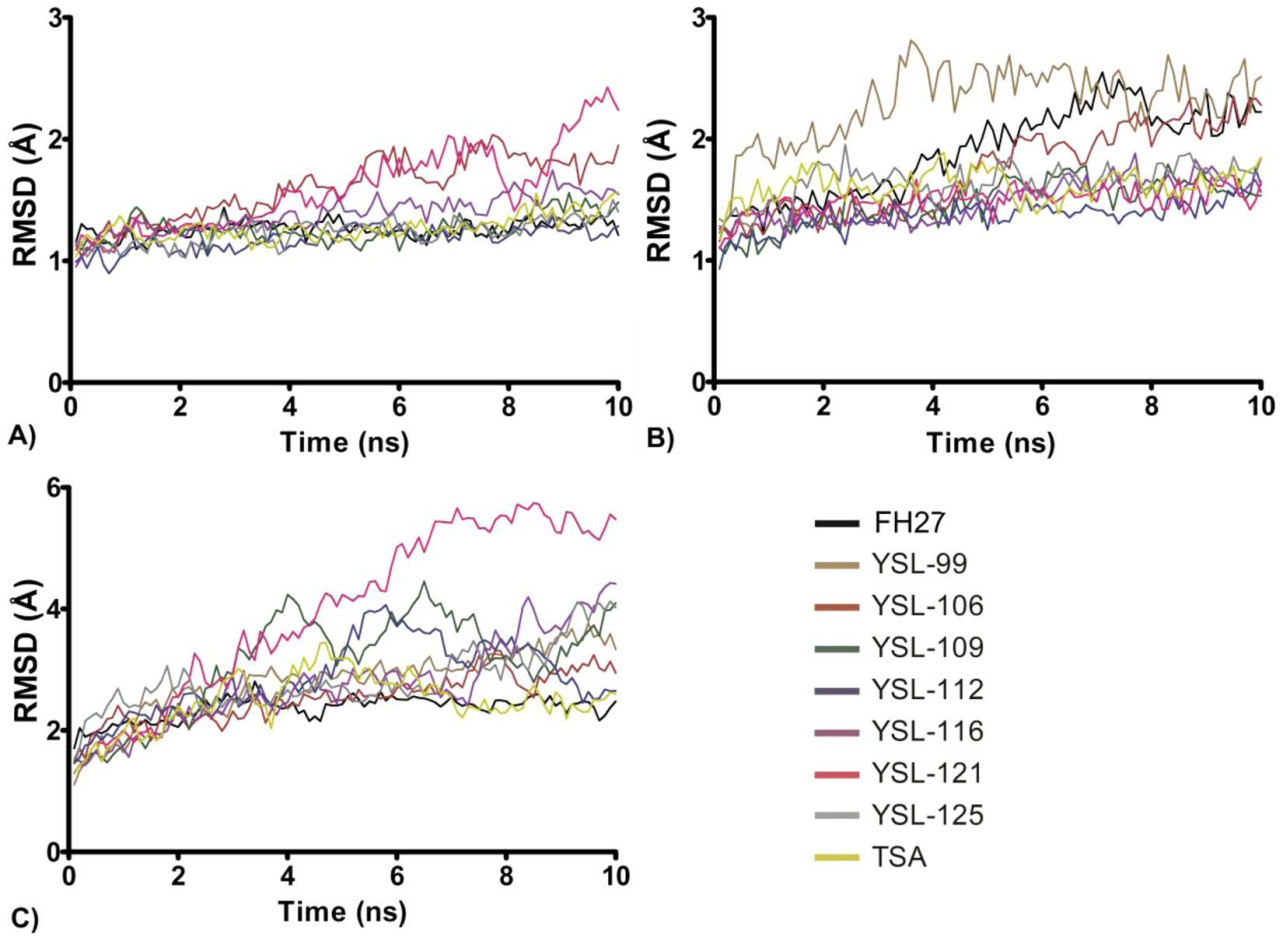
Root mean square deviations (RMSDs) of the ligand-HDAC complexes. (**A**) RMSDs of HDAC1-ligand, (**B**) RMSDs of HDAC6-ligand, and (**C**) RMSDs of HDAC8-ligand. Figure built with GraphPad software.

**Figure 3:**
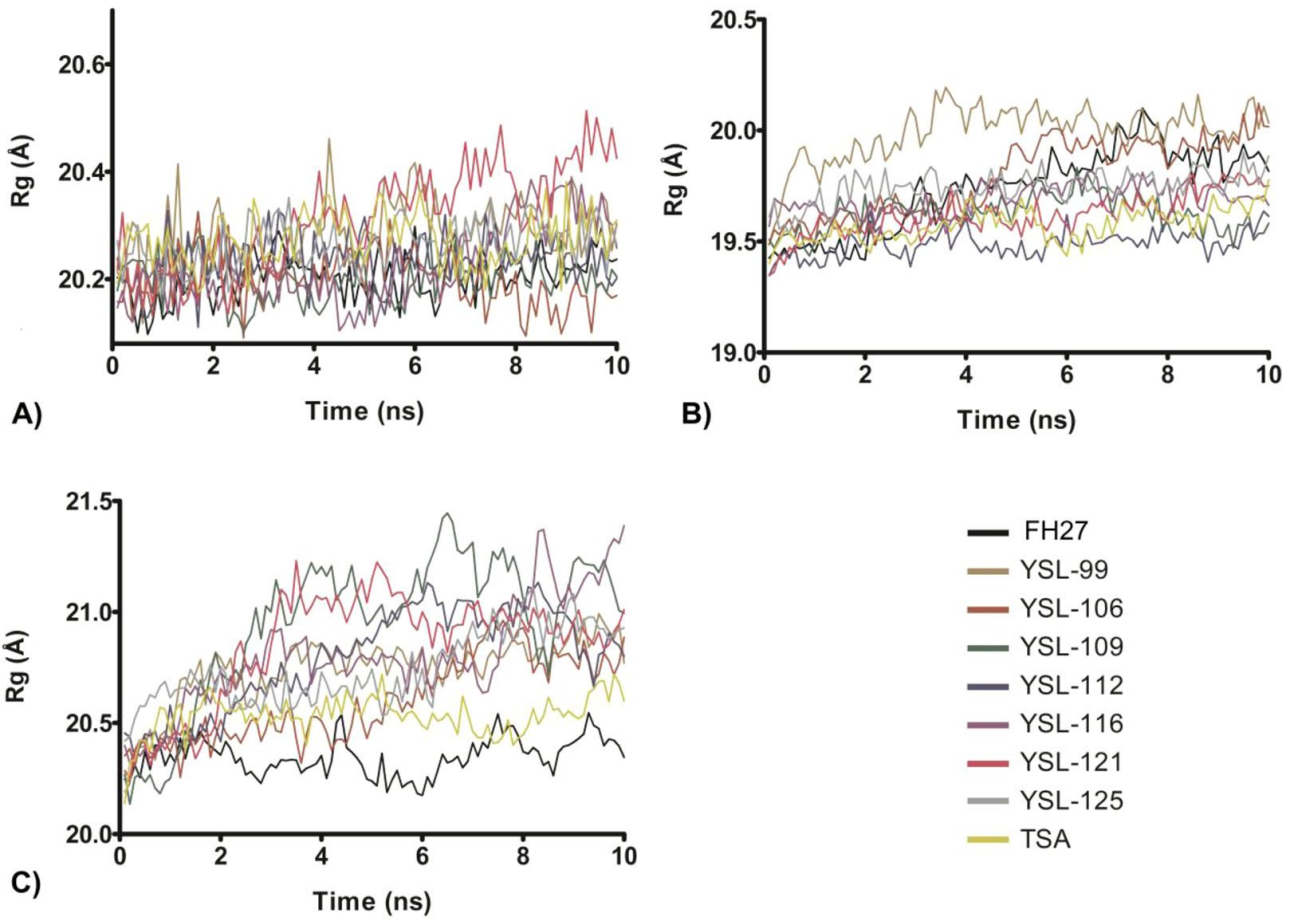
Radius of gyration (Rg) of the ligand-HDAC complexes. (**A**) Rg of HDAC1-ligand, (**B**) Rg of HDAC6-ligand, and (**C**) Rg of HDAC8-ligand. Figure built with GraphPad software.

To determine the average deviations in the atomic positions and stability under the MD simulations, the RMSD values of the trajectories of the protein-ligand complexes were calculated (Supplementary information, Table S2). The RMSD showed that the HDAC1-ligand complex reached a faster constant structural behavior (Fig. 2A) than the protein-ligand complexes formed with HDAC6 (Fig. 2B) and HDAC8 (Fig. 2C). HDAC1 showed mean RMSD values between 1.24 and 1.87 Å, while the RMSD values for HDAC6 were between 1.54 and 2.45 Å and those for the HDAC8 complexes were between 2.45 and 5.38 Å (Table S2). The Rg values of the complexes in HDAC1 ranged from 20.194 Å ± 0.037 Å to 20.388 Å ± 0.055 Å (Fig. 3A), for HDAC6, this range was from 19.506 Å ± 0.050 Å to 20.0494 Å ± 0.066 Å (Fig. 3B), and for HDAC8, this range was from 20.352 Å ± 0.093 Å to 21.117 Å ± 0.146 Å (Fig. 3C), demonstrating that the three systems have a similar degree of compactness (Supplementary information, Table S2). Based on this analysis, the first 10 ns were excluded from the structural and energetic analyses.

**Figure 4:**
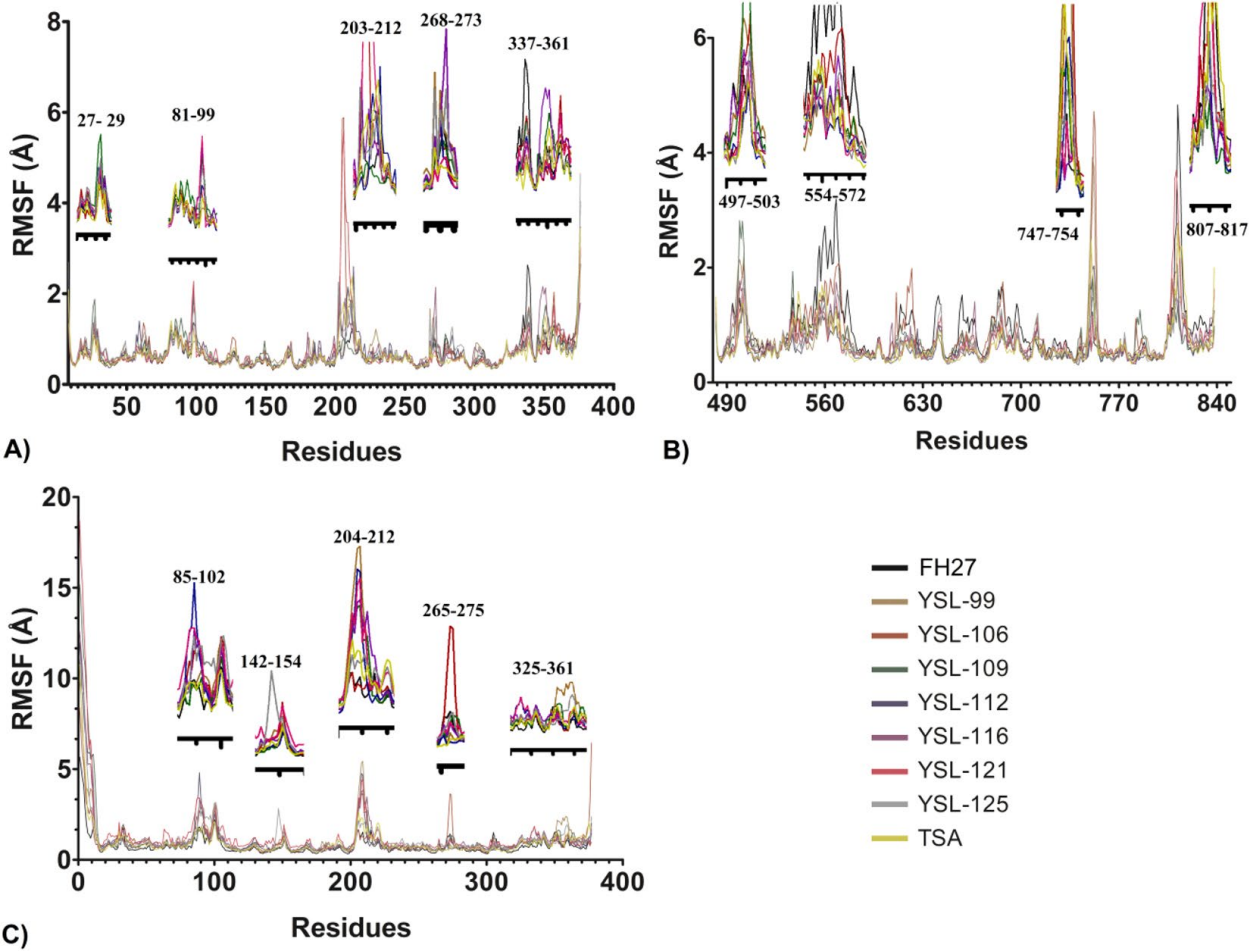
Root mean square fluctuations (RMSFs) of ligand-HDAC complexes. (**A**) RMSFs of HDAC1-ligand, (**B**) RMSFs of HDAC6-ligand, and (**C**) RMSFs of HDAC8-ligand. Regions with higher fluctuations were enlarged and depicted in the upper part or on the side of the corresponding region. Figure built with GraphPad software.

The RMSF analysis for the HDAC1 complexes showed that YSL-121 and YSL-106 displayed higher fluctuations (>3 Å) in the E203-R212 region, while YSL-109 decreased the RMSF below 1 Å. Moreover, the other ligands oscillated between 2.6 Å−1 Å. This E203-R212 region is part of the loop surrounding the cap region that leads to the catalytic site. In apo-HDAC1, this region was also observed with higher RMSF values. The target ligands were able to reduce the RMSF in comparison to the apo-HDAC1 model^52^. Other regions with relatively higher fluctuations are the G27-P29, P81-D99, and G268-C273 regions, whose structural behaviors were also found in a previous work of ligands with inhibitory activity against HDAC1^52^. However, the E337-F341 and S346-E361 regions also displayed high fluctuations, a structural feature that had not been reported for HDAC1-ligand complexes (Fig. 4A). These E337-F341 and S346-E361 regions are interconnected and form part of a long loop region away from the catalytic tunnel (Supplementary information, Fig. S3), revealing that loops not only near to the catalytic tunnel undergo structural modifications to accommodate ligands into the catalytic site.

In the HDAC6 complexes, the fluctuations of catalytic domain 2 (G482-G800) were less than that observed (~4 Å) in a previous study of apo-HDAC6 domain 2. However, higher fluctuations did exist in the D497-V503 region (<2.8 Å) and M554-C572 region (<3.2 Å), which is minor portion of apo-HDAC6 domain 2 (<4 Å).^37^. There are two regions, D747-V754 and T807-L817 (Fig. 4B), that display the highest fluctuations (<4.7 Å and <4.8 Å, respectively), of which D747-V754 belongs to a loop region adjacent to the catalytic site, and T807-L817 is also part of a loop, but this loop is further from the catalytic site; therefore, the same phenomenon that was described for HDAC1 was also observed for HDAC6 domain 2 (Fig. 3B) (Supplementary information, Fig. S4).

In the case of the HDAC8-ligand complexes (Fig. 3C), the structural fluctuations were higher from M1 to Q12, which is part of a missing loop in crystal structures since it has substantial motility and high conformational flexibility (higher than 5 Å) that was reproduced by our MD simulations. This behavior was also found for this region in apo-HDAC8 in a previous work^32^. On the other hand, the residues belonging to loop 2 (E85-D102), loop 3 (H142-Y154), loop 5 (K204-G212), loop 7 (G265-C275) and loop 9 (K325-R361) displayed higher fluctuations in comparison to the other regions of the protein (Fig. 4C). YSL-112 showed the highest RMSF for loop 2 (>4 Å), and the other ligands (YSL-99, YSL-106, YSL-109, YSL-112, YSL-116, YSL-121 and YSL-125) showed values in the range of 2.5 Å to 3.4 Å. In loop 3, this value ranged from 0.6 Å to 2.83 Å, and higher values were observed for YSL-125, YSL-121 and YSL-106 (1.3–2.83 Å); the other ligands showed lower values (<1.3 Å). In loop 5, compounds YSL-99, YSL-109, YSL-112, YSL-116 and YSL-121 showed RMSF values from 3.6 Å to 5.4 Å, and YSL-106 and YSL-125 displayed values from 1.6 Å to 2.2 Å. In loop 7, only FH27, YSL-106, YSL1–09, YSL-116, YSL-121 and YS-L125 showed higher fluctuations (0.90–3.57 Å), with the highest fluctuation observed for YSL-106 (>1.5 Å). In contrast, in loop 9, all the complexes showed fluctuations higher than 1.0 Å in this region and all were higher than 1.5 Å except for YSL-106. Thus, these regions (L2, L3, L5 and L7) represent a zone of higher fluctuation in holo-HDAC8, and all except L9 are adjacent to the catalytic site; of which L9 is far from the catalytic site but is affected by the ligand coupling process. L2, L5, L7 and L9 showed higher fluctuations in Apo-HDAC8 but L3 did not, which, in this work, only some ligands were able to produce increased fluctuations in this region^32^. Hence, the residues that belong to loops 2 and 5 participated in the accommodation of ligands into the catalytic tunnel, which is in line with previous reports^29,32^.

Further, MD simulations of the carboxylic acid derivatives (the target compounds with carboxylic acid instead of hydroxamic acid) in complex with HDAC1, HDAC6 and HDAC8 were also evaluated to test whether the hydroxamic or carboxylic groups are the zinc binding group (ZBG) that guide the binding of the hydroxamic acids synthetized in this work. However, it was found that most of the carboxylic acids were not able of reaching the Zn 2+ atom through the carboxylic groups, in spite carboxylic acid is a demonstrated ZBG^53^, instead they were inserted by the butyl chain into the catalytic tunnel locating away from Zn 2+ (>4.5 Å), being anchored at the entrance of the catalytic tunnel by the aromatic alkyl portion (Supplementary information, Fig. S5). So, based on these findings we hypothesized that hydroxamic group do not govern the recognition of some of these molecules, and the aromatic portion might be the responsible for the binding.

### Free energy calculations

**Figure 5:**
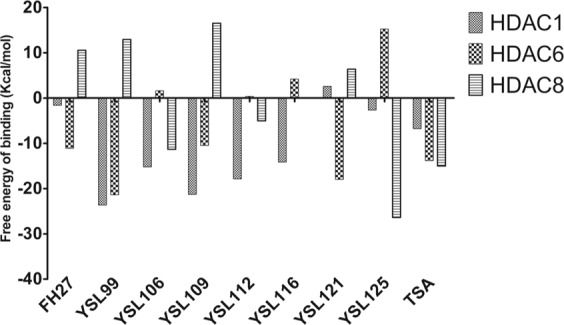
Free energy of binding of the HDAC-ligand complexes calculated by the MMGBSA method. Figure built with GraphPad software.

Furthermore, the free energies of binding (**FEBs)** among the ligands and HDACs were estimated using the MMGBSA approach. In Fig. 5, the free energies of the HDAC-ligand complexes are shown. FH27 and YSL-121 showed higher affinity for HDAC6 in comparison to that observed for HDAC1 and HDAC8, while YSL-99, YSL-106, YSL-109, YSL-112 and YSL-116 showed a greater affinity for HDAC1 in comparison to HDAC6 and HDAC8, and only YSL-125 showed favorable affinity for HDAC8. Interestingly, the compounds with a higher affinity for HDAC1 were those with better cytotoxic activity against the cancer cell lines evaluated (Table 2).

Table 3 shows the FEBs, the molecular mechanics components, and polar (**ΔEelec** + **ΔGGB)** and nonpolar (**ΔEvdw** + **ΔGSA)** solvation energy terms. In HDAC1 complexes, the nonpolar contributions (ΔEvdw + ΔGSA) dominate in all HDAC1-ligand complexes, except for YS-L121, in which the non-favorable polar (ΔEelec + ΔGGB) contributions predominate, rendering unfavorable complex interactions. Similar behavior was observed for the complexes FH27-HDAC1 and YSL-125-HDAC1, where nonpolar contributions screened this effect yielding FEB values of −1.61 ad −2.65 kcal/mol, respectively. In the YSL-99, YSL-106, YSL-109 and YSL-116 complexes with HDAC1, favorable contributions from the terms ΔEvdw, ΔESA, ΔEelec were detected; however, the largest unfavorable ΔGGB and stronger polar solvation energy contributions were also observed, which was screened by the other terms. However, for YSL-112-HDAC1, a fine balance between the polar and nonpolar contributions was observed, giving a favorable FEB, but in general, hydrophobic interactions guided the binding. The affinity of the inhibitors for HDAC1 was as follows: YSL-99> YSL-109> YSL-112> YSL-106> YSL-116> TSA > YSL-125 > FH27 > YSL-121.

**Table 3:** Free energy of binding of the compounds with antiproliferative effects against HDAC1, HDAC6 and HDAC8 obtained using the MMGBSA method^a^.

HDAC1	FEB^b^	ΔEvdw^c^	ΔEelec^d^	ΔGGB^e^	ΔGSA^f^	Polar^g^	Nonpolar^h^
FH27	−1.6153	−48.572	11.960	41.2212	−6.224	53.1812	−54.796
YSL-99	−23.632	−37.568	−12.969	32.1939	−5.2883	19.2249	−42.8563
YSL-106	−15.21	−53.225	−3.1995	47.353	−6.1385	44.1535	−59.3635
YSL-109	−21.29	−55.998	−10.796	52.5679	−7.0636	41.7719	−63.0616
YSL-112	−17.862	−58.685	7.8076	40.0951	−7.0799	47.9027	−65.7649
YSL-116	−14.147	−48.161	−1.5793	41.8915	−6.2982	40.3122	−54.4592
YSL-121	2.5817	−51.013	26.555	32.9591	−5.9194	59.5141	−56.9324
YSL-125	−2.6567	−49.37	35.3306	17.7303	−6.3474	53.0609	−55.7174
TSA	−6.7754	−43.166	17.2393	24.9624	−5.8115	42.2017	−48.9775
**HDAC6**	**FEB**^**b**^	**ΔEvdw**^**c**^	**ΔEelec**^**d**^	**ΔGGB**^**e**^	**ΔGSA**^**f**^	**Polar**^**g**^	**Nonpolar**^**h**^
FH27	−11.114	−27.296	2.3508	17.7179	−3.8874	20.0687	−31.1834
YSL-99	−21.391	−36.127	−34.073	53.8762	−5.0668	19.8032	−41.1938
YSL-106	−2.1265	−39.457	−3.0172	49.3126	−5.2188	46.2954	−44.6758
YSL-109	−10.488	−41.373	−8.3282	44.7596	−5.5466	36.4314	−46.9196
YSL-112	0.3457	−37.519	−7.9263	50.4008	−4.6101	42.4745	−42.1291
YSL-116	4.1838	−34.8674	−20.228	65.0665	−5.7877	44.8385	−40.6551
YSL-121	−17.977	−31.547	−63.657	81.8689	−4.6418	18.2119	−36.1888
YSL-125	15.2455	−45.498	32.7453	34.1884	−6.1907	66.9337	−51.6887
TSA	−13.825	−28.234	−32.349	50.5371	−3.7793	18.1881	−32.0133
**HDAC8**	**FEB**^**b**^	**ΔEvdw**^**c**^	**ΔEelec**^**d**^	**ΔGGB**^**e**^	**ΔGSA**^**f**^	**Polar**^**g**^	**Nonpolar**^**h**^
FH27	10.5606	−35.149	10.5555	39.8472	−4.6935	50.4027	−39.8425
YSL-99	12.9324	−28.784	−0.3995	46.3877	−4.2718	45.9882	−33.0558
YSL-106	−11.346	−34.388	−33.614	61.6079	−4.9523	27.9939	−39.3403
YSL-109	16.5194	−37.752	19.7418	39.5541	−5.0239	59.2959	−42.7759
YSL-112	−5.0453	−44.672	−4.3563	50.0299	−6.0464	45.6736	−50.7184
YSL-116	0.0635	−42.224	15.1292	32.5402	−5.3817	47.6694	−47.6057
YSL-121	6.3736	−35.485	−13.08	59.2932	−4.3547	46.2132	−39.8397
YSL-125	−26.383	−40.522	−80.572	99.3852	−4.674	18.8132	−45.196
TSA	−15.012	−25.169	−39.216	53.3842	−4.0107	14.1682	−29.1797

^a^All energies are in kcal/mol, ^b^FEB = Free energy of binding, ^c^ΔEvdw = Contribution to the FEB from the van der Waals energy, ^d^ΔEelec = Contribution to the FEB from the electrostatic energy, ^e^ΔGGB = Contribution to the FEB from the polar solvation energy, ^f^ΔGSA = Contribution to the FEB from the solvent accessible surface energy

In the HDAC6 complexes, nonpolar contributions also dominated the FEB values, despite the higher unfavorable contribution from ΔGGB, which was screened by the nonpolar contribution ΔGelec. For YSL-125, nonpolar contributions predominated, rendering an unfavorable FEB for the complex, and with FH27, a balance between the contributions was observed, giving a favorable FEB. The affinity of the inhibitors for HDAC6 was as follows: YSL-99> YSL-121> TSA > FH27 > YSL-109> YSL-112> YSL-106> YSL-116> YSL-125.

In HDAC8 complexes, more variability was observed since fewer compounds showed favorable FEB values for this isoform (only TSA, YSL-106, YSL-112 and YSL-125), in which cases the nonpolar contributions dominated the FEB even though the ΔGGB values were higher as screened by the favorable nonpolar and ΔGelec contributions. In the other complexes, the polar contributions were higher than the nonpolar contributions, yielding an unfavorable FEB. The affinity of the inhibitors for HDAC8 was as follows: YSL-125 > TSA > YSL-106> YSL-112> YSL-116> YSL-121 > FH27 > YSL-99. Overall, the favorable interactions established between the ligand and the protein are mainly mediated by hydrophobic contacts.

### HDAC activity

**Figure 6:**
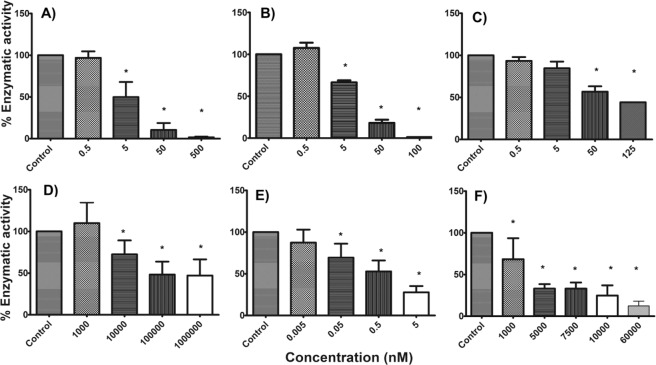
Evaluation of the enzymatic activity of TSA against (**A**) HDAC1, (**B**) HDAC6 and (**C**) HDAC8 and YSL-109 against (**D**) HDAC1, (**E**) HDAC6 and (**F**) HDAC8 obtained from two distinct experiments in duplicate. *P < 0.05. Figure built with GraphPad software.

The *in silico* studies (binding pose) and antiproliferative assays guided us to select the best HDAC inhibitory activity of YSL-109 as evaluated against HDAC1, HDAC6 and HDAC8. YSL-109 inhibited HDAC6 in a highly selective manner (0.537 nM) compared with HDAC8 and HDAC1, and showed 4000-fold selectivity (Table 4). Thus, YSL-109 is a highly selective HDAC6 inhibitor that is more potent than TSA (Fig. 6) or the other inhibitors that have been reported [7].

**Table 4:** IC_50_ values of YSL−109 against HDACs.

	IC_50_ (nM)		
	HDAC1	HDAC6	HDAC8
**YSL-109**	259439.68	0.537	2242.72
**TSA**	7.61	9.72	98.83

## Conclusion

A set of hydroxamic acid derivatives were designed, submitted to a virtual screening protocol evaluating the ADME/Tox properties in combination with MD simulations and molecular docking studies for HDAC1, HDAC6 and HDAC8, and validated with *in vitro* assays on pure enzymes. Then, the most promising compounds were assayed in a panel of cancer cell lines, which identified that YSL-109 showed promising activity against hepatocellular carcinoma (HepG2 cell line, IC_50_ = 3.39 µM), breast cancer (MCF-7 cell line, IC_50_ = 3.41 µM; HCC1954 cell line, IC_50_ = 3.41 µM) and neuroblastoma (SH-SY5Y cell line, IC_50_ = 6.42 µM). In addition, MD simulation studies were not able to reproduce the experimental selectivity. Through this screening, we obtained a highly selective HDAC6 inhibitor with an IC_50_ of 0.537 nM.

## Experimental section

### Computational methodology

#### Molecular modeling

The crystal structure of HDAC1 was obtained from the Protein Data Bank (PDB ID: 4BKX). Water, sulfate and acetate molecules were stripped from the system. Structural potassium ions were left due to their importance in the catalytic activity as suggested by their presence and conserved position among HDAC isoforms^33^. The *in silico* studies revealed that these ions play a structural role since their absence masks the motility of the loops adjacent to the catalytic sites^52^. For HDAC6, catalytic domain 2 (DD2-HDAC6) was retrieved from a previous work^37^. The structure of HDAC8 was taken from the PDB (PDB entry 3F07), and the missing loop region (M1-Q12) was modeled using Modeller^54^ as described in a previous work^32^. Between isoforms, the amino acid sequence identity and similarity for HDAC1 and DD2-HDAC6 are 24.6% and 40.7%, for HDAC1 and HDAC8 are 40.1% and 61.5%, and for HDAC8 and DD2-HDAC6 are 24.9% and 40.9%, respectively^55^.

#### Molecular docking

The compounds were submitted for molecular docking using Autodock4Zn, an improved force field of AutoDock for the docking of zinc metalloproteins^51^. Ligands were prepared with AutoDock Tools 1.5.7^56^. Focused docking was performed, and the grid box was centered on Zn^2+^ with dimensions of 44 × 30 × 30 Å to 48 × 30 × 30 Å and a grid space of 0.375 Å^3^. For scoring sampling, a Lamarckian-Genetic Algorithm with a randomized initial population of 100 individuals and a maximum number of energy evaluations of 1 × 10^7^ with 200 runs were performed. A cluster analysis was performed. The cluster in which hydroxamic acid was coordinated with the Zn^2+^ atom was used as the starting conformation for performing the ligand-protein MD simulations.

#### Molecular dynamics simulations

MD simulations of the ligand-receptor proteins were 10 ns long and carried out with the AMBER 12 package^57^. Ligand parameters were generated with the antechamber module, based on the generalized AMBER force field (GAFF)^58^ and the AM1-BCC atomic charges were calculated with the antechamber module. To explore the ligand-HDAC6 and ligand-HDAC8 complexes, the force field ff99SB^59^ was used, and for the ligand-HDAC1 complex, the force field ff12SB^60^ was used due to a previous study performed in our group in which the role of potassium ions in the structure of HDAC1 was suggested^52^. For carboxylic acid derivatives, the forcefield ff14SB was used. The systems were neutralized with Na^+^ ions, centered into a rectangular 12 Å box and solvated with a water TIP3P model^61^. The tleap module was used to prepare the protein and minimized with a sander module. The MD simulations were run with pmemd.cuda executable^57^ using graphical units processors^62,63^. Zn^2+^ was replaced by a tetrahedron-shaped zinc divalent cation conformed by 4 cationic dummy atoms around the central zinc^64,65^. Energy minimization was carried out through 1000 steps of steepest descent followed by 1000 steps of conjugate gradients and equilibrated through 100 picoseconds (ps) of heating and 100 ps of density equilibration with weak restraints on the complex followed by 300 ps of constant pressure equilibration at 300 K. The SHAKE algorithm^66^ was applied to the hydrogen atoms with a step time of 2 femtoseconds and a Langevin thermostat for temperature control. 10 ns and 25 ns of MD simulations for hydroxamic acid and carboxylic acid, respectively, were carried out without position restraints under periodic boundary conditions using the NPT ensemble at 300 K. The electrostatic term was described through the particle mesh Ewald method^67^, and for van der Waals interactions, a 10.0 Å cutoff was used. In the MD simulations, the SHAKE algorithm was used, and the time step was set to 2.0 fs. The temperature and pressure were maintained with the weak coupling algorithm^68^ with coupling constants τT and τp of 1.0 and 0.2 ps, respectively (300 K, 1 atm). Coordinates were saved every 1 ps. To analyze the trajectories, the ptraj module of AMBER 12^57^ was used. Translation and rotational movements were removed from the protein before the calculation of the root mean square deviation (RMSD). Root means square fluctuations (RMSFs) and the radius of gyration (Rg) were used to determine when the trajectories reached equilibrium.

#### Energetic analysis

Once the MD simulations reached equilibrium, the binding free energy was calculated using the MMGBSA method and the single trajectory approach^69^, employing MMPbSA.py script^70^, in a salt concentration of 0.1 M and a Born implicit solvent model of 2 (igb = 2)^71^. Water molecules and metal ions were stripped from the system prior to performing the calculation, which was performed at intervals of 10 ps. With the MMGBSA method, the free energy of binding (ΔG_*bind*_) of the complex in solution was estimated to be^72^ as follows:

1 $${\Delta {\rm{G}}}_{{\rm{bind}}}={\Delta {\rm{E}}}_{{\rm{MM}}}+{\Delta {\rm{G}}}_{{\rm{solv}}}\mbox{--}{\rm{T}}\Delta {\rm{S}}$$

2 $$\Delta {{E}}_{{MM}}=\Delta {{E}}_{{internal}}+\Delta {{E}}_{{electrostatic}}+\Delta {{E}}_{{vdw}}$$

3 $$\Delta {{G}}_{{sol}}=\Delta {{G}}_{{GB}}+\Delta {{G}}_{{SA}}$$

where ΔE_MM_ is the change in gas phase MM energy and includes ΔE_*internal*_ (bond, torsional angle and dihedral energies), ΔE_*electrostatic*_ and van der Waals energies ΔE_*vdw*_. Moreover, ΔG_solv_ is the sum of the electrostatic solvation energy (polar contribution, ΔE_*GB*_) and nonelectrostatic solvation component (nonpolar contributions, ΔG_*SA*_). The polar contribution was calculated using the GB model, while the nonpolar energy was estimated by the solvent accessible surface (SASA). The conformational entropy change -TΔS in the present study was not calculated; therefore, we present the effective binding free energy^69^.

### Chemistry

All compounds were routinely checked by TLC and ^1^H NMR. TLC was performed on aluminum plates coated with silica gel (TLC Plate Silica Gel 60 F_254_), and the spots were visualized with UV light, FeCl_3_ solution, iodine or vanillin, depending on the characteristics of each compound. ^1^H NMR was performed using a 300 MHz Varian Inova spectrometer, and ^13^C NMR was performed using a Varian Direct Drive (75 MHz) spectrometer. The high-resolution electrospray ionization time-of-flight mass spectrometry (HRMS-ESI-TOF) technique was used to obtain a mass spectrum of high resolution, which was performed using a time of flight analyzer (LCT Premier, Waters) on an *Agilent 6545* Q-TOF *LC*/*MS*. The chemical purity was determined by HPLC employing an Agilent 1200 Infinity Series system.

Spectra of low resolution were obtained using a liquid chromatography-mass spectrometry technique with an ESI and a single quadrupole mass detector (Agilent 6110). Liquid chromatography (LC) was performed through automatic injection into the HPLC (binary pump) with a C-18 reversed-phase column (150 × 4.6 mm, Zorvax). The mass analyzer detected in positive ion mode. The solvents used for LC/MS were buffered with 0.1% formic acid. The chromatographic retention time was converted to minutes and seconds in the format 60.60 min.

In the HPLC, two methods were used to evaluate the purity of the compound synthesized:

Method A: Chromatographic parameters: the flow rate was set to 0.8 mL/min, initially isocratic for 2 min (acetonitrile:water 40:60; A), final isocratic at 0 min (acetonitrile; B), gradient from A to B over 10 min.

Method B: Chromatographic parameters: the flow rate was set to 0.8 mL/min, initially isocratic for 2 min (acetonitrile:water 20:80; A), a final isocratic at 0 min (acetonitrile; B), gradient from A to B of 10 min.

The general method of synthesis is described below, but the specific synthetic method is reported together with an example of compound characterization. All compound characterizations can be found in the Supplementary information.

#### Synthesis of the compounds

Solid phase synthesis was carried out on 2-chlorotrityl-*N*-Fmoc-hydroxylamine polymer-bound resin (170 mg, 0.12 mmol, *Iris Biotech GmbH*). The resin was conditioned for 24 h with dimethylformamide (DMF). Deprotection of the hydroxylamine was performed with a solution of 20% piperidine in DMF (3 × 3 mL for 3 min each time) [**Sol. A**]. Then, the resin was washed with DMF (3 × 3 mL). Furthermore, the coupling reaction was carried out with the corresponding *N*-Fmoc-α amino acid (**1**, 0.48 mmol, 4 equivalents, *GI Biochem, Shangai*). As the coupling reagent, **HATU** (1-[bis(dimethylamino)methylene]-1*H*-1,2,3-triazolo[4,5-*b*]pyridinium 3-oxide hexafluorophosphate) or **DIC** (*N*,*N*′-diisopropylcarbodiimide) was used in the presence of the additive **HOAt** (1-hydroxy-7-azabenzotriazole) and **DIPEA** (diisopropylethylamine); an excess of 4 equivalents of each reagent was used regarding the charge of the resin (0.48 mmol of each compound; *Gl Biochem Shangai, Iris Biotech GmbH, Sigma-Aldrich*, respectively) [**Sol. B**]. **Sol. B** was allowed to react at room temperature with 1 min of preactivation time, and the reaction was carried out for 1 h at room temperature (Scheme 2). After this time, the resin was washed three times with three milliliters (3 × 3 mL) of DMF. Deprotection of the Fmoc-α-amino acid (**3**) was performed with **Sol. A**. Finally, the carboxylic acid (**5**) was coupled using an excess of 4 equivalents and **Sol. B** (Scheme 2), following the acylation reaction conditions mentioned above.

The final compound (**7**) was obtained after resin cleavage using 4 mL of 5% trifluoroacetic acid solution (TFA) in DCM/triethylsilane (TES) [**Sol. C**] for 30 min (Scheme 2). Then, the product was recovered by filtration, and the resin was washed with 5 mL of **Sol. C** and DCM (5 × 3 mL). The resulting solution was carefully evaporated to dryness in a rotary evaporator. The solid obtained was purified by recrystallization with diethyl ether (previously cooled) or using flash chromatography.

General Synthetic Procedure for synthesis of compound N-(2-(hydroxyamino)-2-oxoethyl)benzamide **(GH38)**. The procedure was followed starting from 0.12 mmol of resin, HATU (187 mg, 0.49 mmol), HOAt (67 mg, 0.49 mmol), DIPEA (86 µL, 0.49 mmol), Fmoc-glycine (146 mg, 0.49 mmol) and benzoic acid (60 mg, 0.49 mmol). The compound was purified through a flash chromatography using as mobile phase AcOEt/MeOH/MeCN/H_2_O (70/2.5/1.25/1.25). A white solid was obtained (0.015 g, 57%). ^**1**^**H NMR** (300 MHz, DMSO-*d*6) δ 10.59 (s, 1H, OH_acid_), 8.80 (s, 1H, NH_acid_), 8.72 (s, 1H, NH_amide_), 7.90 (d, *J* = 7.5 Hz, 2H, H-2, H-6), 7.57–741 (m, 3H, H-3, H-4, H-5), 3.81 (d, *J* = 5.5 Hz, 2H, H-1′). ^**13**^**C NMR** (75 MHz, DMSO-*d6*) δ 166.5 (CO_acid_), 166.0 (CO_amide_), 134.0 (C-1), 131.3 (C-4), 128.2 (C-3, C-5), 127.4 (C-2, C-6), 40.6 (C-1′). **HRMS (ESI):** Calculated for [C_9_H_10_N_2_O_3_Na]^+^ [M + Na]^+^ 217.0589, found 217.0592. **HPLC** (Method B, λ: 230 nm): t_R_ 2.51, HPLC purity = 100%.

General Synthetic Procedure for synthesis of compound N-(2-(hydroxyamino)-2-oxoethyl)-4-methyl benzamide ***(GH18)***. The procedure was followed starting from 0.12 mmol of resin, HATU (179 mg, 0.49 mmol), HOAt (64 mg, 0.47 mmol), DIPEA (82 µL, 0.47 mmol), Fmoc-glycine (140 mg, 0.47 mmol) and 4-methylbenzoic acid (65 mg, 0.47 mmol). The compound was purified through a flash chromatography using as mobile phase AcOEt/MeOH/MeCN/H_2_O (70/2.5/1.25/1.25). A white solid was obtained (0.01 g, 40%). ^**1**^**H NMR** (300 MHz, DMSO-*d*6) δ 8.77 (sa, 1H, NH_acid_), 8.60 (sa, 1H, NH_amide_), 7.77 (d, *J* = 9.0 Hz, 2H, H-2, H-6), 7.27 (d, *J* = 9.0 Hz, 2H, H-3, H-5), 3.77 (d, *J* = 6.0 Hz, 2H, H-1′), 2.35 (s, 3H, H-7). ^**13**^**C NMR** (75 MHz, DMSO-*d*6) δ 166.3 (CO_acid_), 166.0 (CO_amide_), 141.1 (C-4), 131.2 (C-1), 128.7 (C-3, C-5), 127.3 (C-2, C-6), 40.4 (C-1′), 20.9 (C-7). **HRMS (ESI)** calculated for [C_10_H_12_N_2_O_3_]^+^ [M + Na]^+^ 231.0746; found 231.0753. **HPLC** (Method B, λ: 240 nm): t_R_ 3.35, HPLC purity = 100%.

General Synthetic Procedure for synthesis of compound 4-butyl-N-(2-(hydroxyamino)-2-oxoethyl)benzamide ***(GH27)****.* The procedure was followed starting from 0.12 mmol of resin, HATU (184 mg, 0.48 mmol), HOAt (66 mg, 0.48 mmol), DIPEA (84 µL, 0.48 mmol), Fmoc-glycine (144 mg, 0.48 mmol) and 4-butylbenzoic acid (86 mg, 0.48 mmol). The compound was purified through a flash chromatography using as mobile phase AcOEt/MeOH/MeCN/H_2_O (70/2.5/1.25/1.25). A white solid was obtained (0.008 g, 26%). ^**1**^**H NMR** (300 MHz, DMSO-*d*6): δ 10.57 (sa, 1H, OH_acid_), 8.78 (sa, 1H, NH_acid_), 8.62 (sa, 1H, NH_amide_), 7.80 (d, *J* = 9.0 Hz, 2H, H-2, H-6), 7.29 (d, *J* = 9.0 Hz, 2H, H-3, H-5), 3.79 (d, *J* = 6.0 Hz, 2H, H-1′), 2.64 (t, *J* = 7.5 Hz, 2H, H-7), 1.58 (quintet, *J* = 7.5 Hz, 2H, H-8), 1.30 (m, *J* = 7.5 Hz, 2H, H-9), 0.91 (t, *J* = 7.5 Hz, 3H, H-10). ^**13**^**C NMR** (75 MHz, DMSO-*d*6): δ 166.5 (CO_acid_), 166.2 (CO_amide_), 146.1 (C-4), 131.7 (C-1), 128.3 (C-2, C-6), 127.5 (C-3, C-5), 40.7 (C-1′), 34.8 (C-7), 33.0 (C-8), 21.9 (C-9), 13.9 (C-10). **HRMS (ESI)**: calculated for [C_13_H_18_N_2_O_3_Na]^+^ [M + Na]^+^ 273.1215; found 273.1221. **HPLC** (Method A, λ: 230 nm): t_R_ 3.20, HPLC purity = 100%.

General Synthetic Procedure for synthesis of compound (S)-N-(1-(hydroxyamino)-1-oxo-3-phenylpropan-2-yl)benzamide **(FH38)**. The procedure was followed starting from 0.12 mmol of resin, HATU (184 mg, 0.48 mmol), HOAt (66 mg, 0.48 mmol), DIPEA (84 µL, 0.48 mmol), Fmoc-L-Phenylalanine (187 mg, 0.48 mmol) and benzoic acid (59 mg, 0.48 mmol). The compound was purified through recrystallization from DCM. A white solid was obtained (0.008 g, 23%). ^**1**^**H NMR** (300 MHz, DMSO-*d*6): δ 10.76 (sa, 1H, OH_acid_), 8.87 (sa, 1H, NH_acid_), 8.59 (d, *J* = 6.0 Hz, 1H, NH_amide_), 7.79 (d, *J* = 7.1 Hz, 2H, H-2, H-6), 7.56–7.08 (m, 8H, H-3, H-4, H-5, H-5′, H-6′, H-7′, H-8′, H-9′), 4.60 (dd, *J* = 8.1, 6.0 Hz, 1H, H-2′), 3.02 (d, *J* = 6.0 Hz, 2H, H-3′). **HRMS (ESI)**: calculated for [C_16_H_16_N_2_O_3_Na]^+^ [M + Na]^+^ 307.1059; found 307.1051. **HPLC** (Method A, λ: 214 nm): t_R_ 2.51, HPLC purity = 100%.

##### General Synthetic Procedure for synthesis of compound **(**S)-N-(1-(hydroxyamino)-1-oxo-3-phenylpropan-2-yl)-4-methylbenzamide **(FH18)**.

The procedure was followed starting from 0.12 mmol of resin, HATU (184 mg, 0.48 mmol), HOAt (66 mg, 0.48 mmol), DIPEA (84 µL, 0.48 mmol), Fmoc-L-Phenylalanine (187 mg, 0.48 mmol) and 4-methylbenzoic acid (66 mg, 0.48 mmol). The compound was purified through recrystallization from DCM. A beige solid was obtained (0.016 g, 48%). ^**1**^**H NMR** (300 MHz, DMSO-*d*6): δ 10.75 (sa, 1H, OH_acid_), 8.86 (sa, 1H, NH_acid_), 8.49 (d, *J* = 9.0 Hz, 1H, NH_amide_), 7.71 (d, *J* = 8.0 Hz, 2H, H-2, H-6), 7.37–7.09 (m, 7H, H-3, H-5, H-5′, H-6′, H-7′, H-8′, H-9′), 4.59 (dd, *J* = 15.0, 9.0 Hz, 1H, H-2′), 3.01 (d, *J* = 9.0 Hz, 2H, H-3′), 2.33 (s, 3H, H-7). ^**13**^**C NMR** (75 MHz, DMSO-*d*6): δ 168.2 (CO_acid_), 166.0 (CO_amide_), 141.1 (C-4), 138.3 (C-4′), 131.2 (C-1), 129.2 (C-5′, C-9′), 128.7 (C-6′, C-8′), 128.1 (C-3, C-5), 127.5 (C-2, C-6), 126.3 (C-7′), 52.7 (C-2′), 37.4 (C-3′), 21.0 (C-7). **HRMS (ESI)**: calculated for [C_17_H_19_N_2_O_3_]^+^ [M + H]^+^ 299.1396; found 299.1398. **HPLC** (Method A, λ: 230 nm): t_R_ 9.29, HPLC purity = 100%

##### General Synthetic Procedure for synthesis of compound (S)−4-butyl-N-(1-(hydroxyamino)-1-oxo-3-phenylpropan-2-yl)benzamide **(FH27)**.

The procedure was followed starting from 0.12 mmol of resin, HATU (189 mg, 0.50 mmol), HOAt (68 mg, 0.50 mmol), DIPEA (87 µL, 0.50 mmol), Fmoc-L-Phenylalanine (192 mg, 0.50 mmol) and 4-butylbenzoic acid (88 mg, 0.50 mmol). The compound was purified through recrystallization from DCM. A white solid was obtained (0.028 g, 65%). ^**1**^**H NMR** (300 MHz, DMSO-*d*6): δ 10.75 (sa, 1H, OH_acid_), 8.86 (sa, 1H, NH_acid_), 8.49 (d, *J* = 9.0 Hz, 1H, NH_amide_), 7.72 (d, *J* = 8.0 Hz, 2H, H-2, H-6), 7.42–7.08 (m, 7H, H-3, H-5, H-5′, H-6′, H-7′, H-8′, H-9′), 4.59 (d, *J* = 6.0 Hz, 1H, H-2′), 3.01 (d, *J* = 9.0 Hz, 2H, H-3′), 2.61 (t, *J* = 7.5 Hz, 2H, H-7), 1.56 (m, 2H, H-8), 1.29 (m, 2H, H-9), 0.89 (t, *J* = 7.5 Hz, 3H, H-10). ^**13**^**C NMR** (75 MHz, DMSO-*d*6): δ 168.6 (CO_acid_), 166.5 (CO_amide_), 146.3 (C-4), 138.7 (C-4′), 131.9 (C-1), 129.6 (C-5′, C-9′), 128.5 (C-6′, C-8′), 128.4 (C-2, C-6), 127.9 (C-3, C-5), 126.7 (C-7′), 53.1 (C-2′), 37.9 (C-3′), 35.1 (C-7), 33.3 (C-8), 22.1 (C-9), 14.2 (C-10). **HRMS (ESI)**: calculated for [C_20_H_25_N_2_O_3_]^+^ [M + H]^+^ 341.1865; found 341.1866. **HPLC** (Method A, λ: 240 nm): t_R_ 9.16. HPLC purity = 100%.

##### General Synthetic Procedure for synthesis of compound (S)-N-(1-(hydroxyamino)-1-oxo-3-phenylpropan-2-yl)-2-propylpentanamide **(FH37)**.

The procedure was followed starting from 0.12 mmol of resin, HATU (189 mg, 0.50 mmol), HOAt (68 mg, 0.50 mmol), DIPEA (87 µL, 0.50 mmol), Fmoc-L-Phenylalanine (192 mg, 0.50 mmol) and valproic acid (50 µL, 0.50 mmol). The compound was purified through recrystallization from DCM. A white solid was obtained (0.013 g, 36%). ^**1**^**H NMR** (300 MHz, DMSO-*d*6): δ 10.62 (s, 1H, OH_acid_), 8.84 (s, 1H, NH_acid_), 8.03 (d, *J* = 9.0 Hz, 1H, NH_amide_), 7.33–7.10 (m, 5H, H-5′, H-6′, H-7′, H-8′, H-9′), 4.46 (m, 1H, H-2′), 2.93–2.68 (m, 2H, H-3′), 2.15 (m, 1H, H-1), 1.40–0.92 (m, 6H, H-2(2×), H-3), 0.90–0.60 (m, 8H, H-2, H-3(2×)). ^**13**^**C NMR** (75 MHz, DMSO-*d*6): δ 174.4 (CO_acid_), 168.2 (CO_amide_), 137.7 (C-4′), 129.0 (C-5′, C-9′), 127.8 (C-6′, C-8′), 126.0 (C-7′), 51.2 (C-2′), 44.7 (C-1), 37.9 (C-3′), 34.9 and 34.5 (C-2), 20.0 and 19.6 (C-3), 14.0 and 13.8 (C-4). **HRMS (ESI)**: calculated for [C_17_H_27_N_2_O_3_]^+^ [M + H]^+^ 307.2022; found 307.2026. **HPLC** (Method A, λ: 214 nm): t_R_ 6.37. HPLC purity = 100%

##### General Synthetic Procedure for synthesis of compound **(**S)-4-butyl-N-(1-(hydroxyamino)-3-(4-hydroxyphenyl)-1-oxopropan-2-yl)benzamide **(YSL99)**.

The procedure was followed starting from 0.14 mmol of resin, HATU (216 mg, 0.57 mmol), HOAt (77 mg, 0.57 mmol), DIPEA (99 µL, 0.57 mmol), Fmoc-L-Tyr(*t*Bu)-OH (261 mg, 0.57 mmol) and 4-butylbenzoic acid (101 mg, 0.57 mmol), and additional step was required once the product was cleavage from the resin in order to remove the *N*-Boc group which is removed with 10% trifluoroacetic acid (TFA)/DCM in presence of triethylsilane after 30 min of stirring at room temperature. The compound was purified through a flash chromatography using as mobile phase AcOEt/MeOH/MeCN/H_2_O (70/2.5/1.25/1.25). A beige solid was obtained (0.0043 g, 8.5%). ^**1**^**H NMR** (300 MHz, DMSO-*d*6): δ 10.69 (s, 1H, OH_acid_), 9.11 (s, 1H, OH_phenol_), 8.82 (s, 1H, NH_acid_), 8.40 (d, *J* = 6.0 Hz, 1H, NH_amide_), 7.72 (d, *J* = 8.1 Hz, 2H, H-2, H-6), 7.24 (d, *J* = 8.0 Hz, 2H, H-3, H-5), 7.09 (d, *J* = 8.4 Hz, 2H, H-5′, H-9′), 6.62 (d, *J* = 8.3 Hz, 2H, H-6′, H-8′), 4.50 (dd, *J* = 15.0, 6.0 Hz, 1H, H-2′), 2.89 (d, *J* = 6 Hz, 2H, H-3′), 2.61 (t, *J* = 7.5 Hz, 2H, H-7), 1.55 (quintet, *J* = 7.5 Hz, 2H, H-8), 1.37–1.27 (m, *J* = 7.5 Hz, 2H, H-9), 0.89 (t, *J* = 7.5 Hz, 3H, H-10). ^**13**^**C NMR** (75 MHz, DMSO-*d*6): δ 168.7 (CO_acid_), 166.5 (CO_amide_), 156.2 (C-7′), 146.3 (C-4), 132.0 (C-1), 130.5 (C-5′, C-9′), 128.7 (C-4′), 128.4 (C-2, C-6), 127.9 (C-3, C-5), 115.3 (C-6′, C-8′), 53.5 (C-2′), 37.1 (C-3′), 35.1 (C-7), 33.3 (C-8), 22.2 (C-9), 14.2 (C-10). **HRMS (ESI)**: calculated for [C_20_H_25_N_2_O_4_]^+^ [M + H]^+^ 357.1814; found 357.1814. **HPLC** (Method A, λ: 230 nm): t_R_ 5.29, HPLC purity = 100%.

General Synthetic Procedure for synthesis of compound (R)-4-butyl-N-(1-(hydroxyamino)-1-oxo-3-phenylpropan-2-yl)benzamide **(YSL-106)**. The procedure was followed starting from 0.14 mmol of resin, DIC (57 µ, 0.57 mmol), HOAt (78 mg, 0.57 mmol), Fmoc-D-Phenylalanine (222 mg, 0.57 mmol) and 4-butylbenzoic acid (102 mg, 0.57 mmol). The compound was purified through a recrystallization from DCM. A white solid was obtained (0.0248 g, 51%). ^**1**^**H NMR** (300 MHz, DMSO-*d*6): δ 10.76 (s, 1H, OH_acid_), 8.91 (sa, 1H, NH_acid_), 8.51 (d, *J* = 9.0 Hz, 1H, NH_amide_), 7.74 (d, *J* = 9.0 Hz, 2H, H-2, H-6), 7.43–7.09 (m, 7H, H-3, H-5, H-5′, H-6′, H-7′, H-8′, H-9′), 4.61 (dd, *J* = 13.5, 9 Hz, 1H, H-2′), 3.03 (d, *J* = 9.0 Hz, 2H, H-3′), 2.62 (t, *J* = 7.5 Hz, 2H, H-7), 1.57 (quintet, *J* = 7.5 Hz, 2H, H-8), 1.31 (sextet, *J* = 7.5 Hz, 2H, H-9), 0.90 (t, *J* = 7.5 Hz, 3H, H-10). ^**13**^**C NMR** (75 MHz, DMSO-*d*6): δ 171.3 (CO_acid_), 169.2 (CO_amide_), 149.0 (C-4), 141.4 (C-4′), 134.6 (C-1), 132.2 (C-5′, C-9′), 131.2 (C-6′, C-8′), 131.1 (C-2, C-6), 130.6 (C-3, C-5), 129.4 (C-7′), 55.8 (C-2′), 40.5 (C-3′), 37.7 (C-7), 36.0 (C-8), 24.8 (C-9), 16.9 (C-10). **HRMS (ESI)**: calculated for [C_20_H_25_N_2_O_3_]^+^ [M + H]^+^ 341.1865; found 341.1860. **HPLC** (Method A, λ: 240 nm): t_R_ 9.23, HPLC purity = 100%.

General Synthetic Procedure for synthesis of compound **(**S)-4-butyl-N-(1-(hydroxyamino)-3-(naphthalen-1-yl)-1-oxopropan-2-yl)benzamide **(YSL-109)**. The procedure was followed starting from 0.14 mmol of resin, DIC (57 µ, 0.57 mmol), HOAt (78 mg, 0.57 mmol), Fmoc-3-(1-naphtyl)-L-alanine (251 mg, 0.57 mmol) and 4-butylbenzoic acid (102 mg, 0.57 mmol). The compound was purified through a recrystallization from DCM. A white solid was obtained (0.0365 g, 65%). ^**1**^**H NMR** (300 MHz, DMSO-*d*6): δ 10.88 (s, 1H, OH_acid_), 8.90 (s, 1H, NH_acid_), 8.60 (d, *J* = 9.0 Hz, 1H, NH_amide_), 8.29 (d, *J* = 9.0 Hz, 1H, aromatic), 7.92 (d, *J* = 9 Hz, 1H, aromatic), 7.82–7.20 (m, 9H, aromatic), 4.80 (dd, *J* = 12.0, 6.0 Hz, 1H, H-2′), 3.60 (dd, *J* = 13.5, 4.5 Hz, 1H, H-3a’), 3.44 (dd, *J* = 12.0, 10.5 Hz, 1H, H-3b’), 2.62 (t, *J* = 7.5 Hz, 2H, H-7), 1.56 (quintet, *J* = 7.5 Hz, 2H, H-8), 1.30 (sextet, *J* = 7.5 Hz, 2H, H-9), 0.90 (t, *J* = 6.0 Hz, 3H, H-10). ^**13**^**C NMR** (75 MHz, DMSO-*d*6): δ 171.1 (CO_acid_), 169.2 (CO_amide_), 149.0 (C-4), 137.0 (C-4′), 136.5 (C-aromatic), 134.6 (C-aromatic), 134.5 (C-aromatic), 131.7 (C-aromatic), 131.1 (C-2, C-6), 130.7 (C-3, C-5), 130.6 (C-aromatic), 130.2 (C-aromatic), 129.3 (C-aromatic), 128.7 (C-aromatic), 128.4 (C-aromatic), 126.9 (C-aromatic), 54.9 (C-2′), 37.8 (C-3′), 37.7 (C-7), 36.0 (C-8), 24.8 (C-9), 16.9 (C-10). **HRMS (ESI)**: calculated for [C_24_H_27_N_2_O_3_]^+^ [M + H]^+^ 391.2022; found 391.2016. **HPLC** (Method A, λ: 214 nm): t_R_ 11.17. HPLC purity = 100%

General Synthetic Procedure for synthesis of compound (S)-4-butyl-N-(1-(hydroxyamino)-3-(naphthalen-2-yl)-1-oxopropan-2-yl)benzamide **(YSL-112)**. The procedure was followed starting from 0.14 mmol of resin, DIC (57 µ, 0.57 mmol), HOAt (78 mg, 0.57 mmol), Fmoc-3-(2-naphtyl)-L-alanine (251 mg, 0.57 mmol) and 4-butylbenzoic acid (102 mg, 0.57 mmol). The compound was purified through a recrystallization from DCM. A white solid was obtained (0.0390 g, 70%). ^**1**^**H NMR** (300 MHz, DMSO-*d*6): δ 10.79 (s, 1H, OH_acid_), 8.90 (sa, 1H, NH_acid_), 8.58 (d, *J* = 9.0 Hz, 1H, NH_amide_), 7.90–7.76 (m, 4H, H-naphtyl), 7.74 (d, J = 9.0 Hz, 2H, H-3, H-5), 7.60–7.40 (m, 3H, H- naphtyl), 7.25 (d, *J* = 9.0 Hz, 2H, H-3, H-5), 4.73 (dd, *J* = 15.0, 9.0 Hz, 1H, H-2′), 3.21 (d, *J* = 6.0 Hz, 2H, H-3′), 2.61 (t, *J* = 9.0 Hz, 2H, H-7), 1.55 (quintet, *J* = 7.5 Hz, 2H, H-8), 1.30 (sextet, *J* = 7.5 Hz, 2H, H-9), 0.89 (t, *J* = 7.5 Hz, 3H, H-10). ^**13**^**C NMR** (75 MHz, DMSO-*d*6): δ 171.2 (CO_acid_), 169.2 (CO_amide_), 149.0 (C-4), 139.1 (C-4′), 136.0 (C-1), 134.9 (C-naphthyl), 134.6 (C-naphthyl), 131.1 (C-2, C-6), 130.9 (C-3, C-5), 130.6 (C-naphthyl), 130.5 (C-naphthyl), 130.4 (C-naphthyl), 129.1 (C-naphthyl), 128.5 (C-naphthyl), 55.8 (C-2′), 40.7 (C-3′), 37.7 (C-7), 36.0 (C-8), 24.8 (C-9), 16.9 (C-10). **HRMS (ESI)**: calculated for [C_24_H_27_N_2_O_3_]^+^ [M + H]^+^ 391.2022; found 391.2030. **HPLC** (Method A, λ: 230 nm): t_R_ 11.10. HPLC purity = 100%.

General Synthetic Procedure for synthesis of compound (S)-4-butyl-N-(3-(4-fluorophenyl)-1-(hydroxyamino)-1-oxopropan-2-yl)benzamide **(YSL-116)**. The procedure was followed starting from 0.14 mmol of resin, DIC (57 µ, 0.57 mmol), HOAt (78 mg, 0.57 mmol), Fmoc-L-Phe(4-F)-OH (232 mg, 0.57 mmol) and 4-butylbenzoic acid (102 mg, 0.57 mmol). The compound was purified through a flash chromatography using as mobile phase AcOEt/MeOH/MeCN/H_2_O (70/2.5/1.25/1.25). A salmon color solid was obtained (0.102 g, 43%). ^**1**^**H NMR** (300 MHz, DMSO-*d*6): δ 10.76 (s, 1H, OH_acid_), 8.89 (s, 1H, NH_acid_), 8.52 (d, *J* = 8.6 Hz, 1H, NH_amide_), 7.74 (d, *J* = 8.0 Hz, 2H, H-2, H-6), 7.36 (dd, *J* = 8.0 Hz, ^4^*J*_H-F_ = 6.0 Hz, 2H, H-5′, H-9′), 7.26 (d, *J* = 7.9 Hz, 2H, H-3, H-5), 7.09 (dd, *J* = 8.8 Hz, ^3^*J*_H-F_ = 8.8 Hz, 2H, H-6′, H-8′), 4.58 (dd, *J* = 15.3, 7.9 Hz, 1H, H-2′), 3.02 (d, *J* = 7.2 Hz, 2H, H-3′), 2.63 (t, *J* = 7.6 Hz, 2H, H-7), 1.57 (quintet, *J* = 7.6 Hz, 2H, H-8), 1.31 (sextet, *J* = 7.2 Hz, 2H, H-9), 0.90 (t, *J* = 7.3 Hz, 3H, H-10). ^**13**^**C NMR** (75 MHz, DMSO-*d*6): δ 168.5 (CO_acid_), 166.6 (CO_amide_), 161.4 (C-7′, ^1^*J*_C-F_ = 144 Hz), 146.4 (C-4), 134.9 (C-1), 131.9 (C-4′), 131.4 (C-5′, C-9′, ^3^*J*_C-F_ = 5.3 Hz), 128.5 (C-2, C-6), 128.0 (C-3, C-5), 115.3 (C-6′, C-8′, ^2^*J*_C-F_ = 12.8 Hz), 53.2 (C-2′), 37.0 (C-3′), 35.1 (C-7), 33.4 (C-8), 22.2 (C-9), 14.2 (C-10). **HRMS (ESI)**: calculated for [C_20_H_24_N_2_O_3_F]^+^ [M + H]^+^ 359.1771; found 359.1773. **HPLC** (Method A, λ: 240 nm): t_R_ 9.48. HPLC purity = 100%.

General Synthetic Procedure for synthesis of compound (S)-4-butyl-N-(1-(hydroxyamino)-3-(4-iodophenyl)-1-oxopropan-2-yl)benzamide **(YSL-121)**. The procedure was followed starting from 0.14 mmol of resin, DIC (57 µ, 0.57 mmol), HOAt (78 mg, 0.57 mmol), Fmoc-L-Phe(4-I)-OH (294 mg, 0.57 mmol) and 4-butylbenzoic acid (102 mg, 0.57 mmol). The compound was purified through a recrystallization from DCM. A white solid was obtained (0.044 g, 66%). ^**1**^**H NMR** (300 MHz, DMSO-*d*6): δ 10.76 (s, 1H, OH_acid_), 8.88 (s, 1H, NH_acid_), 8.52 (d, *J* = 9.0 Hz, 1H, NH_amide_), 7.74 (d, *J* = 9.0 Hz, 2H), 7.63 (d, *J* = 9.0 Hz, 2H), 7.27 (d, *J* = 9.0 Hz, 2H), 7.15 (d, *J* = 9.0 Hz, 2H), 4.58 (dd, *J* = 15.0, 9.0 Hz, 1H, H-2′), 2.98 (d, *J* = 6.0 Hz, 2H, H-3′), 2.63 (t, *J* = 7.5 Hz, 2H, H-7), 1.57 (quintet, *J* = 7.5 Hz, 2H, H-8), 1.31 (sextet, *J* = 7.5 Hz, 2H, H-9), 0.91 (t, *J* = 7.5 Hz, 3H, H-10). ^**13**^**C NMR** (75 MHz, DMSO-*d*6): δ 171.0 (CO_acid_), 169.2 (CO_amide_), 149.1 (C-4), 141.2 (C-6′, C-8′), 139.9 (C-4′), 134.7 (C-1), 134.5 (C-5′, C-9′), 131.1 (C-2, C-6), 130.6 (C-3, C-5), 95.4 (C-7′), 55.6 (C-2′), 40.0 (C-3′), 37.7 (C-7), 36.0 (C-8), 24.8 (C-9), 16.9 (C-10). **HRMS (ESI)**: calculated for [C_20_H_24_N_2_O_3_I]^+^ [M + H]^+^ 467.0832; found 467.0838. **HPLC** (Method A, λ: 230 nm): t_R_ 11.28. HPLC purity = 100%.

General Synthetic Procedure for synthesis of compound **(**S)-4-butyl-N-(1-(hydroxyamino)-3-(4-nitrophenyl)-1-oxopropan-2-yl)benzamide **(YSL-125)**. The procedure was followed starting from 0.14 mmol of resin, DIC (57 µ, 0.57 mmol), HOAt (78 mg, 0.57 mmol), Fmoc-L-Phe(4-NO_2_)-OH (248 mg, 0.57 mmol) and 4-butylbenzoic acid (102 mg, 0.57 mmol). The compound was purified through a flash chromatography using as mobile phase AcOEt/MeOH/MeCN/H_2_O (70/2.5/1.25/1.25). A white solid was obtained (0.03 g, 60%). ^**1**^**H NMR** (300 MHz, DMSO-*d*6): δ 8.61 (d, *J* = 9.0 Hz, 1H, NH_amide_), 8.15 (d, *J* = 9.0 Hz, 2H, H-6′, H-8′), 7.72 (d, *J* = 6.0 Hz, 2H, H-2, H-6), 7.60 (d, *J* = 9.0 Hz, 2H, H-5′, H-9′), 7.25 (d, *J* = 6.0 Hz, 2H, H-3, H-5), 4.67 (ddd, *J* = 9.0, 9.0, 6.0 Hz, 1H, H-2′), 3.18 (dd, *J* = 6.0, 3.0 Hz, 1H, H-3′), 2.61 (t, *J* = 9.0 Hz, 2H, H-7), 1.54 (m, *J* = 9.0 Hz, 2H, H-8), 1.29 (sextet, *J* = 9.0 Hz, 2H, H-9), 0.89 (t, *J* = 6.0 Hz, 3H, H-10).^**13**^**C NMR** (75 MHz, DMSO-d6): δ 170.7 (CO_acid_), 169.2 (CO_amide_), 149.8 (C-7′), 149.3 (C-4′), 149.1 (C-4), 134.4 (C-1), 133.6 (C-5′, C-9′), 131.2 (C-2, C-6), 130.6 (C-3, C-5), 126.3 (C-6′, C-8′), 55.3 (C-2′), 40.3 (C-3′), 37.7 (C-7), 36.0 (C-8), 24.8 (C-9), 16.9 (C-10). **HRMS (ESI)**: calculated for [C_20_H_24_N_3_O_5_]^+^ [M + H]^+^ 386.1716; found 386.1714. **HPLC** (Method A, λ: 254 nm): t_R_ 9.34. HPLC purity = 100%.

General Synthetic Procedure for synthesis of compound (S)-4-butyl-N-(2-(hydroxyamino)-2-oxo-1-phenylethyl) benzamide **(YSL-129)**. The procedure was followed starting from 0.14 mmol of resin, DIC (57 µ, 0.57 mmol), HOAt (78 mg, 0.57 mmol), Fmoc-Phg-OH (214 mg, 0.57 mmol) and 4-butylbenzoic acid (102 mg, 0.57 mmol). The compound was purified through a recrystallization from DCM. A white solid was obtained (0.004 g, 8.6%). ^**1**^**H NMR** (300 MHz, DMSO-*d*6): δ 11.02 (sa, 1H, OH_acid_), 8.98 (s, 1H, NH_acid_), 8.72 (d, *J* = 9.0 Hz, 1H, NH_amide_), 7.83 (d, *J* = 6.0 Hz, 2H, H-2, H-6), 7.50 (d, *J* = 6.0 Hz, 2H, H-3, H-5), 7.40–719 (m, 5H, H-5′, H-6′, H-7′, H-8′, H-9′), 5.60 (d, *J* = 6.0 Hz, 1H, H-1′), 2.62 (t, *J* = 7.5 Hz, 2H, H-7), 1.56 (quintet, *J* = 7.5 Hz, 2H, H-8), 1.30 (sextet, *J* = 7.5 Hz, 2H, H-9), 0.89 (t, *J* = 7.5 Hz, 3H, H-10). ^**13**^**C NMR** (75 MHz, DMSO-*d*6): δ 169.8 (CO_acid_), 169.2 (CO_amide_), 149.2 (C-4), 141.6 (C-3′), 134.4 (C-1), 131.3 (C-5′, C-7′), 131.2 (C-2, C-6), 130.9 (C-3, C-5), 130.7 (C-4′, C-8′), 130.5 (C-6′), 57.7 (C-2′), 37.7 (C-7), 36.0 (C-8), 24.8 (C-9), 16.9 (C-10). **HRMS (ESI)**: calculated for [C_19_H_23_N_2_O_3_]^+^ [M + H]^+^ 327.1709; found 327.1707. **HPLC** (Method A, λ: 240 nm): t_R_ 9.11. HPLC purity = 100%.

### Biological evaluation

#### Cytotoxicity test

A colorimetric assay using MTT was used to monitor cell survival and to determine the 50% inhibitory concentration (IC_50_)^44^. All compounds were evaluated in triplicate using six points, 1/3 dilutions, with the highest concentration of 50 µM. The triplicate measurements were placed in three different 96-plates, and 1% DMSO was used as a negative control.

The first sets of 8 compounds (GH38, GH18, GH27, FH38, FH18, FH27 and FH37) were evaluated in different cancer cell lines: HepG2, MCF-7, SH-SY5Y, MIA PaCa-2, and FH27. Furthermore, the second series of eight compounds were evaluated using seven cell lines: SH-SY5Y which is a neuroblastoma cell line; HepG2, a hepatocellular carcinoma cell line; MIA PaCa-2, a human pancreatic carcinoma cell line; MCF-7, a breast cancer cell line; HCC1954, a basal-Her2+ breast carcinoma cell line; RCC4-VA, a renal carcinoma cell line with a VHL mutation; and RCC4-VHL, a renal carcinoma cell without the VHL mutation (wild type). The cells used were seeded in 96-well plates and left for 24 h (5% CO_2_ and 90% humidity, 50000 cells/well) and incubated for 3 days to study the cytotoxicity.

#### Enzymatic inhibition assay

HDAC1 (BML-AK511), HDAC6 (BML-AK516) and HDAC8 (BML-AK518) activity was determined using a commercial assay kit from Enzo Life Sciences (HDAC fluorometric assay/drug discovery kit, Farmingdale, NY, USA). The assay was carried out according to the manufacturer’s instructions. Fluorescence intensity was measured at an excitation wavelength of 360 nm and an emission wavelength of 460 nm using a fluorometer (LS 55, Perkin Elmer, USA). Trichostatin A (TSA) was also evaluated as a positive control. IC_50_ values were determined using GraphPad Prism 5 software.

### Statistical analysis

ANOVA test was performed use to determine any significant difference between each compound treatment and the DMSO control Statistical test and EC_50_ and IC_50_ calculations were performed using GraphPad Prism.

## Supplementary Information

Supplementary Information.
