# Supplementary material for: Hydroxamic acid derivatives as HDAC1, HDAC6 and HDAC8 inhibitors with antiproliferative activity in cancer cell lines
Source: Sci Rep. 2020 Jun 26;10:10462. doi: 10.1038/s41598-020-67112-4 (PMC7320180; doi:10.1038/s41598-020-67112-4)
Supplement: Supplementary file 1 — Supplementary Information. [file 41598_2020_67112_MOESM1_ESM.docx]

***Supplementary Information***

Hydroxamic acid derivatives as HDAC1, HDAC6 and HDAC8 inhibitors with antiproliferative activity in cancer cell lines

Yudibeth Sixto-López^1^, José Antonio [Gómez-Vidal](http://www.ncbi.nlm.nih.gov/pubmed?term=G%C3%B3mez-Vidal%20JA%5BAuthor%5D&cauthor=true&cauthor_uid=18194866)^2^, Nuria de Pedro^3^, Martiniano Bello^†1^, Martha Cecilia Rosales-Hernández^4^, José Correa-Basurto*^1^

^1^ Laboratorio de Diseño y Desarrollo de Nuevos Fármacos e Innovación Biotecnológica (Laboratory for the Design and Development of New Drugs and Biotechnological Innovation)-SEPI, Escuela Superior de Medicina, Instituto Politécnico Nacional, Mexico City, Mexico, 11340.

^2^ Departamento de Química Farmacéutica y Orgánica, Facultad de Farmacia, Universidad de Granada, Granada, Spain, 18071.

^3^ Fundación MEDINA, Centro de Excelencia en Investigación de Medicamentos Innovadores en Andalucía, Granada, Spain, 18016.

^4^ Laboratorio de Biofísica y Biocatálisis, Sección de Estudios de Posgrado e Investigación, Escuela Superior de Medicina, Instituto Politécnico Nacional, Ciudad de México, México.

*[corrjose@gmail.com](mailto:corrjose@gmail.com) or jcorreab@ipn.mx (J. Correa-Basurto)

| **Contents** | **Page No.** |
| --- | --- |
| ***In silico* studies** | **3** |
| ^1^H-NMR and ^13^C-NMR chemical shifts of all the synthesized compounds | **14** |
| ^1^H-NMR and ^13^C-NMR spectra | **21** |

***In silico* studies**

**Table S1**. Results from docking studies using Autodock4Zn forcefield of FH27 and second series of compounds into HDAC1, HDAC6 and HDAC8.

|  | **BFE (kJ/mol)** | **Number of cluster** | **Interactions** | |
| --- | --- | --- | --- | --- |
|  |  |  | **Hydrogen bonds** | **Hydrophobic interactions** |
| **HDAC1** | | | | |
| FH27 | -40.63 | 1 |  | M30, G138, L139, H140, H141, G149, F150, H178, F205, D176, D264, G300, G301, Y303, |
| YSL-99 | -38.87 | 2 | G138, H140, H141, | M30, G138, L139, H140, H141, G149, F150, D176, H178, Y204, F205, D264, L271, G300, G301, Y303, |
| YSL-106 | -26.86 | 5 | H140, N260 | M30, R34, D99, G138, L139, H140, H141, G149, F150, C151, H178, F205, Q260, L271, G300, G301,Y303, |
| YSL-109 | -11.92 | 4 | H140, H141 | M30, L139, H140, H141,G149, F150, C151, D176, H178, Y204, F205, L271, g300, G301, Y303, |
| YSL-112 | -35.81 | 1 | H140, H141 | R34, A136, G137, G138, L139, G149, F150, C151, H174, H178, F205, D264, L271, G300, Y301, Y303, |
| YSL-116 | -37.57 | 1 | H140, H141 | M30, G138, L139, G149, F150, C151, D176, H178, L271, Y204, G300, G301, Y303 |
| YSL-121 | -26.82 | 2 | G149, Y303 | M30, R34, A136, L139, H141, C151, H178, F205, D264, L271, G300, G301, |
| YSL-125 | -30.50 | 2 |  | M30, R34, G138, L138, H140, H141, G149, F150, C151, D176, H178, D164, Y204, F205, L271, G300, G301, Y303, |
| YSL-129 | -24.73 | 3 | D176, H141, G149 | M30, L139, H140, G149, C151, D176, H178, F205, D264, L271, G300, G301, Y303 |
| TSA | -29.79 | 2 |  | M30, R34, L139, H140, H141, G149, F150, D176, H178, F205, G300, G301, Y303, |
| **HDAC6** | | | | |
| FH27 | 146.82 | 5 |  | E502, R506, S568, P608, H610, H611, F620, C621, F680, D649, D742, E779, G780, G781, Y782, |
| YSL-99 | 73.72 | 3 |  | P608, H610, H611,g619, F620, C621, D649, H651, F680, D742, L749, E779, G780, Y782, Zn |
| YSL-106 | 84.26 | 3 | L779 | E502, R506, S568, P608, G609, H610, H611, F620, C621, F680, D649, H651, D742, L749, E779, G780, Y782, Zn |
| YSL-109 | 260.70 | 5 |  | E502, R506, P608, G619,F620, C621, H610, H611, D649, V650, H651, L749,G750, E779, G780, Y782, Zn |
| YSL-112 | 295.01 | 5 | H611, D649 | S568, H610, H611, G619, F620, V650, H651, D742, L749, G780, G781, Y782 |
| YSL-116 | 76.77 | 3 | H611, D649 | E502, T506, P608, H610, G619, F620, C621, D649, H651, F679, F680, L749, E779, G780, Y782 |
| YSL-121 | 118.87 | 2 | D747 | S568, P608, H610, H611, G619, F620, C621, V650, H651, F680, D742, L749, G750, E779, G780, Y782 |
| YSL-125 | 105.31 | 4 | D649 | E502, R506, P608, H610,H611, G619, F620, C621, D649, H651, F679, L749, E779, G780, G781, Y782 |
| YSL-129 | 38.03 | 2 | H610 | E502, R506, P608, H610, H611, G619, F620, H651, F680, D649, L749, E779, G780, Y787 |
| TSA | -6.11 | 7 |  | S568, H611, G619, F620, H651, F679, F680, D742, L749, Y782 |
| **HDAC8** | | | | |
| FH27 | -30.84 | 1 |  | I34, R37, G140, W141, H142, H143, G151, C153, H180, F207, F208, Q263, D267, M274, G303, G304, G305, Y306 |
| YSL-99 | -30.63 | 1 |  | I34, R37, W141, H142, H143, G151, C153, H180, F207, Q263, M274, G304, G305, Y306 |
| YSL-106 | -26.40 | 1 |  | I34, R37, G140, W141, H142, H143, G151, F152, H180, F208,M274, G303, G304, Y306 |
| YSL-109 | 88.19 | 6 | D178, H180 | I34, R37, W141, H142, H143, G151, F152, C153, F208, Q263, G303, G304, G305, Y306 |
| YSL-112 | 3.43 | 1 | G140, H143 | I34, R37, W141, H142, G151, F152, C153, H180, F207, F208, Q263, D267, M274, G304, G305, Y306 |
| YSL-116 | -31.21 | 1 | G140 | I34, R37, W141, H142, H143, G151, F152, H180, F207, F208, Q263, M274, G304, G305, Y306 |
| YSL-121 | -7.28 | 1 | H143 | L33, I34, W141, H142, G151, F152, C153, D178, H180, F208, Q263, G265, G303, G304, Y306 |
| YSL-125 | -11.71 | 1 | D267 | I34, R37, W141,H143, G151, F152, C153, H180, F208, D267, M274, G303, G304, Y306 |
| YSL-129 | -32.802 | 1 | H142 | I34, R37, W141, H142, H143, G151, F152, D178, H180, F207, F208, M274, G303, G304, Y306 |
| TSA | -6.36 | 5 |  | I34, R37, D101, W141, H142, H143, G151, F152, H180, D267, G303, G304, G305, Y306 |

**Table S2**. Root mean square deviation (RMSD) calculated from 10 ns long Molecular dynamic (MD) simulation of the complex compound-HDAC1, -HDAC6 and -HDAC8.

|  | **HDAC1** | | | **HDAC6** | | | **HDAC8** | | |
| --- | --- | --- | --- | --- | --- | --- | --- | --- | --- |
|  | RMSD (Å) | Rg (Å) | Time to reach stability (ns) | RMSD (Å) | Rg (Å) | Time to reach stability (ns) | RMSD (Å) | Rg (Å) | Time to reach stability (ns) |
| FH-27 | 1.279 ± 0.059 | 20.214 ± 0.039 | 2.2 | 2.229 ± 0.138 | 19.894 ± 0.082 | 5.6 | 2.457 ± 0.092 | 20.352 ± 0.093 | 4.7 |
| YSL-99 | 1.815 ± 0.113 | 20.303 ± 0.040 | 6.2 | 2.459 ± 0.168 | 20.0494 ± 0.066 | 3.4 | 3.112 ± 0.267 | 20.814 ± 0.068 | 3.3 |
| YSL-106 | 1.764 ± 0.148 | 20.196 ± 0.047 | 3.7 | 2.073 ± 0.134 | 19.946 ± 0.057 | 5.5 | 2.798 ±0.234 | 20.722 ± 0.151 | 4.3 |
| YSL-109 | 1.277 ± 0.111 | 20.194 ± 0.037 | 2.7 | 1.618 ± 0.088 | 19.649 ± 0.084 | 5 | 3.579 ± 0.388 | 21.117± 0.146 | 3.2 |
| YSL-112 | 1.247 ± 0.052 | 20.239 ± 0.038 | 4.6 | 1.429 ± 0.086 | 19.506 ± 0.050 | 3.1 | 3.297 ± 0.424 | 20.964 ± 0.103 | 4.7 |
| YSL-116 | 1.496 ± 0.089 | 20.255 ± 0.083 | 4.6 | 1.645 ± 0.091 | 19.730 ± 0.049 | 6 | 3.103 ± 0.599 | 20.906± 0.204 | 2.9 |
| YSL-121 | 1.878 ± 0.243 | 20.388 ± 0.055 | 5.1 | 1.541 ± 0.087 | 19.660 ± 0.078 | 3.7 | 5.384 ± 0.222 | 20.943 ± 0.058 | 6 |
| YSL-125 | 1.283 ± 0.083 | 20.277 ± 0.039 | 2.7 | 1.694 ± 0.094 | 19.772 ± 0.053 | 4.2 | 2.899 ± 0.480 | 20.761 ± 0.156 | 0.8 |
| TSA | 1.279 ± 0.099 | 20.277 ± 0.052 | 5.3 | 1.652 ± 0.089 | 19.636 ± 0.054 | 6.8 | 2.672 ± 0.324 | 20.546 ±0.077 | 4.2 |


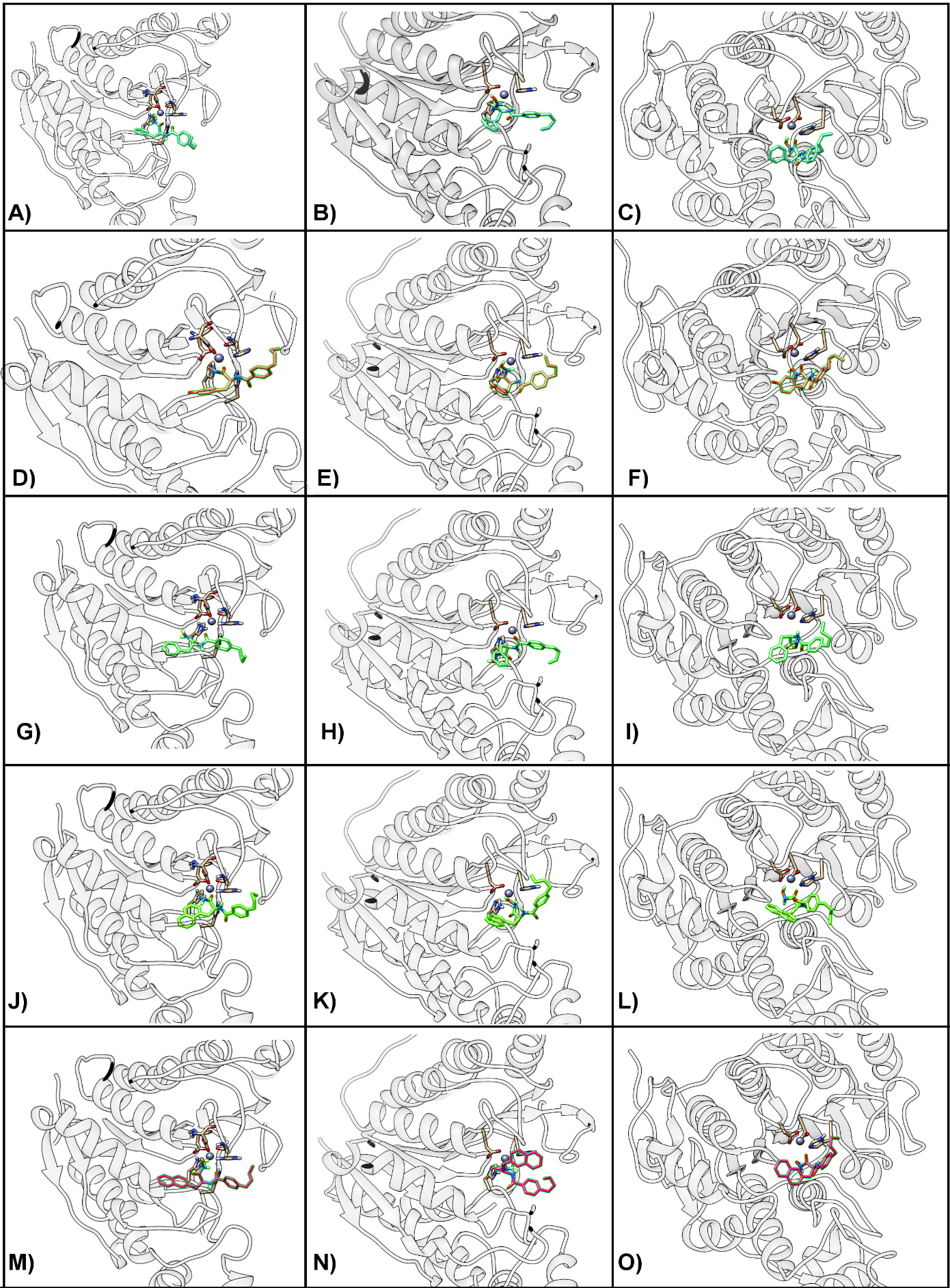


**Figure S1.** Binding mode of compounds studied obtained by molecular docking. A) FH27 with HDAC1, B) FH27 with HDAC6, C) FH27 with HDAC8, D) YSL99 with HDAC1, E) YSL99 with HDAC6, F) YSL99 with HDAC8, G) YSL106 with HDAC1, H) YSL106 with HDAC6, I) YSL106 with HDAC8, J) YSL109 with HDAC1, K) YSL109 with HDAC6, L) YSL109 with HDAC8, M) YSL112 with HDAC1, N) YSL112 with HDAC6, O) YSL112 with HDAC8. Protein’s backbone is depicted as ribbon and in withe color, zinc is represented as gray sphere, and asparagine and histidine residues with those who Zn atom is coordinated are represented as brown stick; meanwhile ligands are depicted as sticks colored in different colors (Ligands are colored as follows: FH27 in blues sky, YSL99 in pink, YSL106 in green pistachio, YSL109 in green yellow, and YSL112 in deep pink).


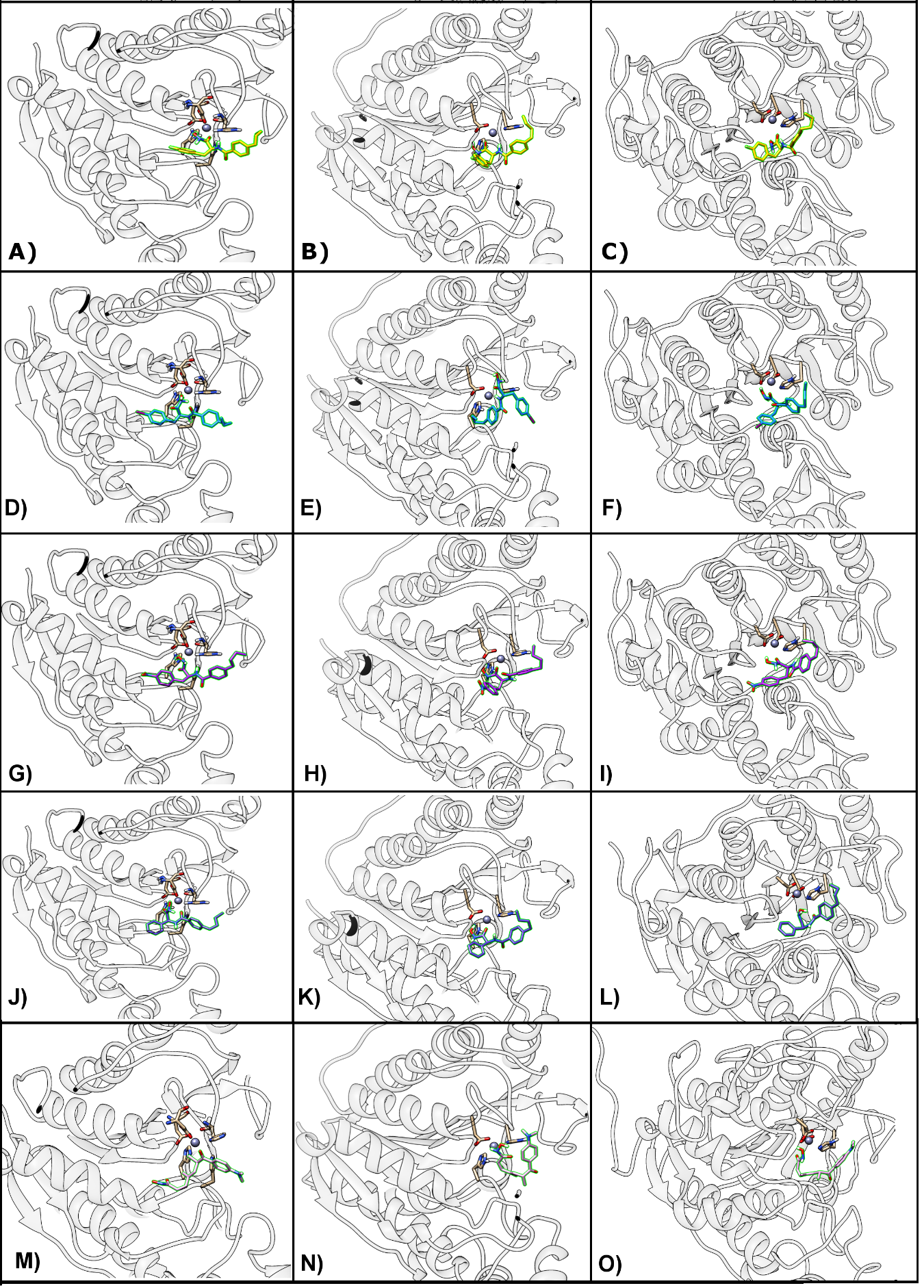


**Figure S2.** Binding mode of compounds studied obtained by molecular docking. A) YSL116 with HDAC1, B) YSL116 with HDAC6, C) YSL116 with HDAC8, D) YSL21 with HDAC1, E) YSL21 with HDAC6, F) YSL21 with HDAC8, G) YSL125 with HDAC1, H) YSL125 with HDAC6, I) YSL125 with HDAC8, J) YSL129 with HDAC1, K) YSL129 with HDAC6, L) YSL129 with HDAC8, M) Trichostatin A with HDAC1, N) Trichostatin A with HDAC6, O) Trichostatin A with HDAC8. Protein’s backbone is depicted as ribbon and in withe color, zinc is represented as gray sphere, and asparagine and histidine residues with those who Zn atom is coordinated are represented as brown stick; meanwhile ligands are depicted as sticks colored in different colors (Ligands are colored as follows: YSL116 in yellow, YSL121 in blue, YSL125 in purple, YSL129 in marine blue and Trichostatin A in lavender color).


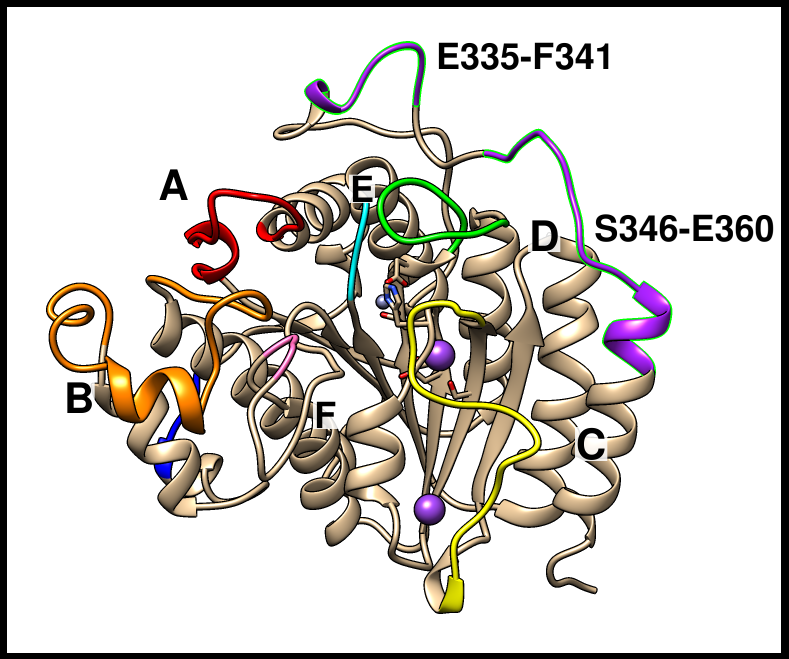


**Figure S3**. HDAC1 structure where regions with higher fluctuations are highlighted, it was taken and modified with author authorization[^1^](#_ENREF_1) (data to be published), in this figure are highlighted the region E335-F341 and S346-E30 that also suffer structural modifications in order to accommodate ligands into the catalytic tunnel. Zinc is depicted as gray spheres and potassium ions are depicted as purple spheres, region A is red color, B is orange color, C yellow color, D is green color, E is cyan color F is pink color and in purple color are depicted region E335-F341 and S346-E360.


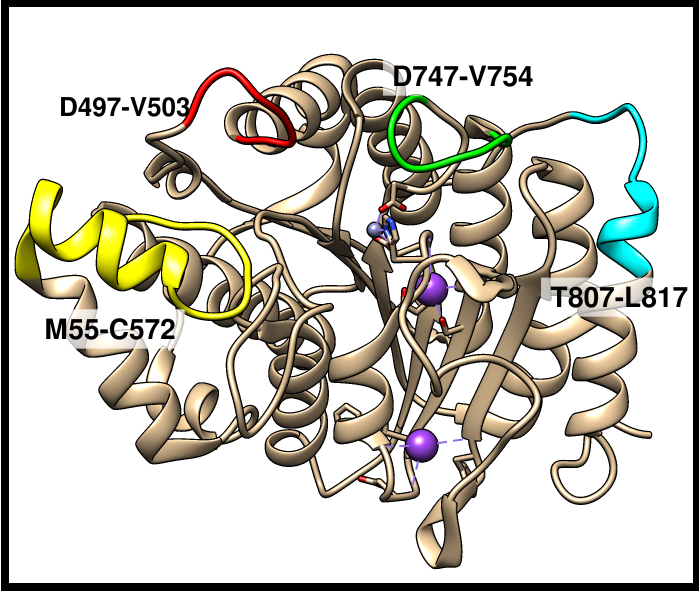


**Figure S4**. HDAC6 regions where regions with higher fluctuations are depicted. Zinc is depicted as gray spheres and potassium ions are depicted as purple spheres, D497-V503 are red color, M554-C57 are yellow color, D747-L754 are green color, T807-L817are cyan color.


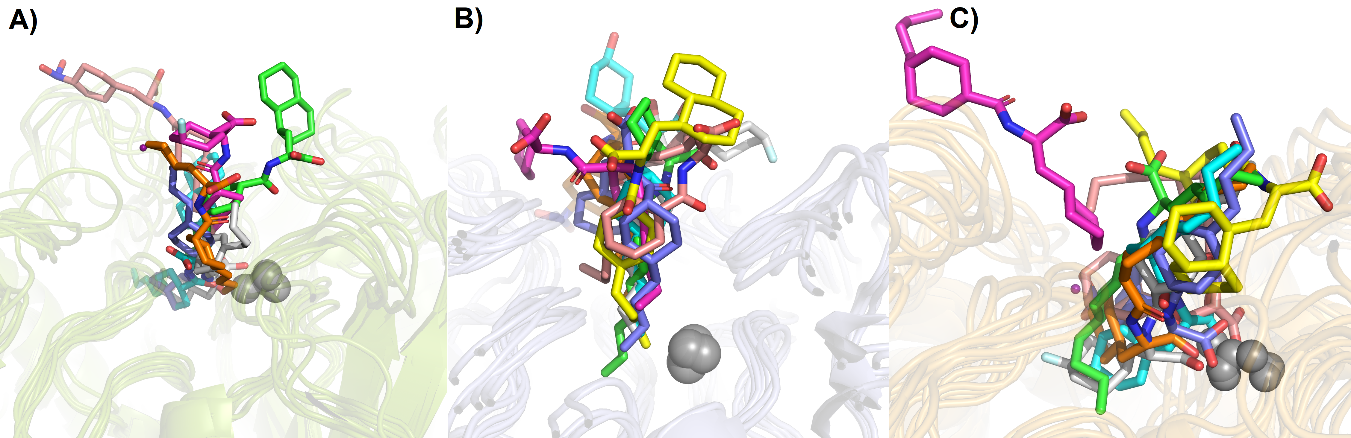


**Figure S5**. Most populated conformation of A) HDAC1, B) HDAC6 and C) HDAC8 in complex with carboxylic acid derivatives of FH27, YSL99, YSL106, YSL109, YSL112, YSL116, YSL121 and YSL125. Zinc are depicted as gray spheres,

**^1^H-NMR and ^13^C-NMR chemical shifts and HRMS of all the synthesized compounds**

N-(2-(hydroxyamino)-2-oxoethyl)benzamide **(GH38).**

White solid was obtained (0.015 g, 57%). **^1^H NMR** (300 MHz, DMSO-*d*6) δ 10.59 (s, 1H, OH_acid_), 8.80 (s, 1H, NH_acid_), 8.72 (s, 1H, NH_amide_), 7.90 (d, *J* = 7.5 Hz, 2H, H-2, H-6), 7.57-741 (m, 3H, H-3, H-4, H-5), 3.81 (d, *J* = 5.5 Hz, 2H, H-1’). **^13^C NMR** (75 MHz, DMSO-*d6*) δ 166.5 (CO_acid_), 166.0 (CO_amide_), 134.0 (C-1), 131.3 (C-4), 128.2 (C-3, C-5), 127.4 (C-2, C-6), 40.6 (C-1’). **HRMS (ESI):** Calculated for [C_9_H_10_N_2_O_3_Na]^+^ [M+Na]^+^ 217.0589, found 217.0592. **HPLC** (Method B, λ: 230 nm): t_R_ 2.51

N-(2-(hydroxyamino)-2-oxoethyl)-4-methyl benzamide **(GH18)**.

White solid was obtained (0.01 g, 40%). **^1^H NMR** (300 MHz, DMSO-*d*6) δ 8.77 (sa, 1H, NH_acid_), 8.60 (sa, 1H, NH_amide_), 7.77 (d, *J* = 9.0 Hz, 2H, H-2, H-6), 7.27 (d, *J* = 9.0 Hz, 2H, H-3, H-5), 3.77 (d, *J* = 6.0 Hz, 2H, H-1’), 2.35 (s, 3H, H-7). **^13^C NMR** (75 MHz, DMSO-*d*6) δ 166.3 (CO_acid_), 166.0 (CO_amide_), 141.1 (C-4), 131.2 (C-1), 128.7 (C-3, C-5), 127.3 (C-2, C-6), 40.4 (C-1’), 20.9 (C-7). **HRMS (ESI)** calculated for [C_10_H_12_N_2_O_3_]^+^ [M+Na]^+^ 231.0746; found 231.0753. **HPLC** (Method B, λ: 240 nm): t_R_ 3.35

4-butyl-N-(2-(hydroxyamino)-2-oxoethyl)benzamide **(GH27).**

White solid was obtained (0.008 g, 26%). **^1^H NMR** (300 MHz, DMSO-*d*6): δ 10.57 (sa, 1H, OH_acid_), 8.78 (sa, 1H, NH_acid_), 8.62 (sa, 1H, NH_amide_), 7.80 (d, *J* = 9.0 Hz, 2H, H-2, H-6), 7.29 (d, *J* = 9.0 Hz, 2H, H-3, H-5), 3.79 (d, *J* = 6.0 Hz, 2H, H-1’), 2.64 (t, *J* = 7.5 Hz, 2H, H-7), 1.58 (quintet, *J* = 7.5 Hz, 2H, H-8), 1.30 (m, *J* = 7.5 Hz, 2H, H-9), 0.91 (t, *J* = 7.5 Hz, 3H, H-10). **^13^C NMR** (75 MHz, DMSO-*d*6): δ 166.5 (CO_acid_), 166.2 (CO_amide_), 146.1 (C-4), 131.7 (C-1), 128.3 (C-2, C-6), 127.5 (C-3, C-5), 40.7 (C-1’), 34.8 (C-7), 33.0 (C-8), 21.9 (C-9), 13.9 (C-10). **HRMS (ESI)**: calculated for [C_13_H_18_N_2_O_3_Na]^+^ [M+Na]^+^ 273.1215; found 273.1221. **HPLC** (Method A, λ: 230 nm): t_R_ 3.20

(S)-N-(1-(hydroxyamino)-1-oxo-3-phenylpropan-2-yl)benzamide **(FH38)**.

White solid was obtained (0.008 g, 23%). **^1^H NMR** (300 MHz, DMSO-*d*6): δ 10.76 (sa, 1H, OH_acid_), 8.87 (sa, 1H, NH_acid_), 8.59 (d, *J* = 6.0 Hz, 1H, NH_amide_), 7.79 (d, *J* = 7.1 Hz, 2H, H-2, H-6), 7.56 – 7.08 (m, 8H, H-3, H-4, H-5, H-5’, H-6’,H-7’, H-8’, H-9’), 4.60 (dd, *J* = 8.1, 6.0 Hz, 1H, H-2’), 3.02 (d, *J* = 6.0 Hz, 2H, H-3’). **HRMS (ESI)**: calculated for [C_16_H_16_N_2_O_3_Na]^+^ [M+Na]^+^ 307.1059; found 307.1051. **HPLC** (Method A, λ: 214 nm): t_R_ 2.51

**(**S)-N-(1-(hydroxyamino)-1-oxo-3-phenylpropan-2-yl)-4-methylbenzamide **(FH18)**.

Beige solid was obtained (0.016 g, 48%). **^1^H NMR** (300 MHz, DMSO-*d*6): δ 10.75 (sa, 1H, OH_acid_), 8.86 (sa, 1H, NH_acid_), 8.49 (d, *J* = 9.0 Hz, 1H, NH_amide_), 7.71 (d, *J* = 8.0 Hz, 2H, H-2, H-6), 7.37 – 7.09 (m, 7H, H-3, H-5, H-5’, H-6’, H-7’, H-8’, H-9’), 4.59 (dd, *J* = 15.0, 9.0 Hz, 1H, H-2’), 3.01 (d, *J* = 9.0 Hz, 2H, H-3’), 2.33 (s, 3H, H-7). **^13^C NMR** (75 MHz, DMSO-*d*6): δ 168.2 (CO_acid_), 166.0 (CO_amide_), 141.1 (C-4), 138.3 (C-4’), 131.2 (C-1), 129.2 (C-5’, C-9’), 128.7 (C-6’, C-8’), 128.1 (C-3, C-5), 127.5 (C-2, C-6), 126.3 (C-7’), 52.7 (C-2’), 37.4 (C-3’), 21.0 (C-7). **HRMS (ESI)**: calculated for [C_17_H_19_N_2_O_3_]^+^ [M+H]^+^ 299.1396; found 299.1398. **HPLC** (Method A, λ: 230 nm): t_R_ 9.29

(S)-4-butyl-N-(1-(hydroxyamino)-1-oxo-3-phenylpropan-2-yl)benzamide **(FH-27)**

White solid was obtained (0.028 g, 65%). **^1^H NMR** (300 MHz, DMSO-*d*6): δ 10.75 (sa, 1H, OH_acid_), 8.86 (sa, 1H, NH_acid_), 8.49 (d, *J* = 9.0 Hz, 1H, NH_amide_), 7.72 (d, *J* = 8.0 Hz, 2H, H-2, H-6), 7.42 – 7.08 (m, 7H, H-3, H-5, H-5’, H-6’, H-7’, H-8’, H-9’), 4.59 (d, *J* = 6.0 Hz, 1H, H-2’), 3.01 (d, *J* = 9.0 Hz, 2H, H-3’), 2.61 (t, *J* = 7.5 Hz, 2H, H-7), 1.56 (m, 2H, H-8), 1.29 (m, 2H, H-9), 0.89 (t, *J* = 7.5 Hz, 3H, H-10). **^13^C NMR** (75 MHz, DMSO-*d*6): δ 168.6 (CO_acid_), 166.5 (CO_amide_), 146.3 (C-4), 138.7 (C-4’), 131.9 (C-1), 129.6 (C-5’, C-9’), 128.5 (C-6’, C-8’), 128.4 (C-2, C-6), 127.9 (C-3, C-5), 126.7 (C-7’), 53.1 (C-2’), 37.9 (C-3’), 35.1 (C-7), 33.3 (C-8), 22.1 (C-9), 14.2 (C-10). **HRMS (ESI)**: calculated for [C_20_H_25_N_2_O_3_]^+^ [M+H]^+^ 341.1865; found 341.1866. **HPLC** (Method A, λ: 240 nm): t_R_ 9.16.

(S)-N-(1-(hydroxyamino)-1-oxo-3-phenylpropan-2-yl)-2-propylpentanamide **(FH37)**

White solid was obtained (0.013 g, 36%). **^1^H NMR** (300 MHz, DMSO-*d*6): δ 10.62 (s, 1H, OH_acid_), 8.84 (s, 1H, NH_acid_), 8.03 (d, *J* = 9.0 Hz, 1H, NH_amide_), 7.33 – 7.10 (m, 5H, H-5’, H-6’, H-7’, H-8’, H-9’), 4.46 (m, 1H, H-2’), 2.93 – 2.68 (m, 2H, H-3’), 2.15 (m, 1H, H-1), 1.40 – 0.92 (m, 6H, H-2(2x), H-3), 0.90 - 0.60 (m, 8H, H-2, H-3(2x)). **^13^C NMR** (75 MHz, DMSO-*d*6): δ 174.4 (CO_acid_), 168.2 (CO_amide_), 137.7 (C-4’), 129.0 (C-5’, C-9’), 127.8 (C-6’, C-8’), 126.0 (C-7’), 51.2 (C-2’), 44.7 (C-1), 37.9 (C-3’), 34.9 and 34.5 (C-2), 20.0 and 19.6 (C-3), 14.0 and 13.8 (C-4). **HRMS (ESI)**: calculated for [C_17_H_27_N_2_O_3_]^+^ [M+H]^+^ 307.2022; found 307.2026.. **HPLC** (Method A, λ: 214 nm): t_R_ 6.37.

**(**S)-4-butyl-N-(1-(hydroxyamino)-3-(4-hydroxyphenyl)-1-oxopropan-2-yl)benzamide **(YSL99)**

Beige solid was obtained (0.0043 g, 8.5%). **^1^H NMR** (300 MHz, DMSO-*d*6): δ 10.69 (s, 1H, OH_acid_), 9.11 (s, 1H, OH_phenol_), 8.82 (s, 1H, NH_acid_), 8.40 (d, *J* = 6.0 Hz, 1H, NH_amide_), 7.72 (d, *J* = 8.1 Hz, 2H, H-2, H-6), 7.24 (d, *J* = 8.0 Hz, 2H, H-3, H-5), 7.09 (d, *J* = 8.4 Hz, 2H, H-5’, H-9’), 6.62 (d, *J* = 8.3 Hz, 2H, H-6’, H-8’), 4.50 (dd, *J* = 15.0, 6.0 Hz, 1H, H-2’), 2.89 (d, *J* = 6 Hz, 2H, H-3’), 2.61 (t, *J* = 7.5 Hz, 2H, H-7), 1.55 (quintet, *J* = 7.5 Hz, 2H, H-8), 1.37 – 1.27 (m, *J* = 7.5 Hz, 2H, H-9), 0.89 (t, *J* = 7.5 Hz, 3H, H-10). **^13^C NMR** (75 MHz, DMSO-*d*6): δ 168.7 (CO_acid_), 166.5 (CO_amide_), 156.2 (C-7’), 146.3 (C-4), 132.0 (C-1), 130.5 (C-5’, C-9’), 128.7 (C-4’), 128.4 (C-2, C-6), 127.9 (C-3, C-5), 115.3 (C-6’, C-8’), 53.5 (C-2’), 37.1 (C-3’), 35.1 (C-7), 33.3 (C-8), 22.2 (C-9), 14.2 (C-10). **HRMS (ESI)**: calculated for [C_20_H_25_N_2_O_4_]^+^ [M+H]^+^ 357.1814; found 357.1814. **HPLC** (Method A, λ: 230 nm): t_R_ 5.29

(R)-4-butyl-N-(1-(hydroxyamino)-1-oxo-3-phenylpropan-2-yl)benzamide (**YSL-106**).

White solid was obtained (0.0248 g, 51%). **^1^H NMR** (300 MHz, DMSO-*d*6): δ 10.76 (s, 1H, OH_acid_), 8.91 (sa, 1H, NH_acid_), 8.51 (d, *J* = 9.0 Hz, 1H, NH_amide_), 7.74 (d, *J* = 9.0 Hz, 2H, H-2, H-6), 7.43 – 7.09 (m, 7H, H-3, H-5, H-5’, H-6’, H-7’, H-8’, H-9’), 4.61 (dd, *J* = 13.5, 9 Hz, 1H, H-2’), 3.03 (d, *J* = 9.0 Hz, 2H, H-3’), 2.62 (t, *J* = 7.5 Hz, 2H, H-7), 1.57 (quintet, *J* = 7.5 Hz, 2H, H-8), 1.31 (sextet, *J* = 7.5 Hz, 2H, H-9), 0.90 (t, *J* = 7.5 Hz, 3H, H-10). **^13^C NMR** (75 MHz, DMSO-*d*6): δ 171.3 (CO_acid_), 169.2 (CO_amide_), 149.0 (C-4), 141.4 (C-4’), 134.6 (C-1), 132.2 (C-5’, C-9’), 131.2 (C-6’, C-8’), 131.1 (C-2, C-6), 130.6 (C-3, C-5), 129.4 (C-7’), 55.8 (C-2’), 40.5 (C-3’), 37.7 (C-7), 36.0 (C-8), 24.8 (C-9), 16.9 (C-10). **HRMS (ESI)**: calculated for [C_20_H_25_N_2_O_3_]^+^ [M+H]^+^ 341.1865; found 341.1860. **HPLC** (Method A, λ: 240 nm): t_R_ 9.23.

**(**S)-4-butyl-N-(1-(hydroxyamino)-3-(naphthalen-1-yl)-1-oxopropan-2-yl)benzamide **(YSL-109)**.

White solid was obtained (0.0365 g, 65%). **^1^H NMR** (300 MHz, DMSO-*d*6): δ 10.88 (s, 1H, OH_acid_), 8.90 (s, 1H, NH_acid_), 8.60 (d, *J* = 9.0 Hz, 1H, NH_amide_), 8.29 (d, *J* = 9.0 Hz, 1H, aromatic), 7.92 (d, *J* = 9 Hz, 1H, aromatic), 7.82 – 7.20 (m, 9H, aromatic), 4.80 (dd, *J* = 12.0, 6.0 Hz, 1H, H-2’), 3.60 (dd, *J* = 13.5, 4.5 Hz, 1H, H-3a’), 3.44 (dd, *J* = 12.0, 10.5 Hz, 1H, H-3b’), 2.62 (t, *J* = 7.5 Hz, 2H, H-7), 1.56 (quintet, *J* = 7.5 Hz, 2H, H-8), 1.30 (sextet, *J* = 7.5 Hz, 2H, H-9), 0.90 (t, *J* = 6.0 Hz, 3H, H-10). **^13^C NMR** (75 MHz, DMSO-*d*6): δ 171.1 (CO_acid_), 169.2 (CO_amide_), 149.0 (C-4), 137.0 (C-4’), 136.5 (C-aromatic), 134.6 (C-aromatic), 134.5 (C-aromatic), 131.7 (C-aromatic), 131.1 (C-2, C-6), 130.7 (C-3, C-5), 130.6 (C-aromatic), 130.2 (C-aromatic), 129.3 (C-aromatic), 128.7 (C-aromatic), 128.4 (C-aromatic), 126.9 (C-aromatic), 54.9 (C-2’), 37.8 (C-3’), 37.7 (C-7), 36.0 (C-8), 24.8 (C-9), 16.9 (C-10). **HRMS (ESI)**: calculated for [C_24_H_27_N_2_O_3_]^+^ [M+H]^+^ 391.2022; found 391.2016. **HPLC** (Method A, λ: 214 nm): t_R_ 11.17.

(S)-4-butyl-N-(1-(hydroxyamino)-3-(naphthalen-2-yl)-1-oxopropan-2-yl)benzamide **(YSL-112).**

White solid was obtained (0.0390 g, 70%). **^1^H NMR** (300 MHz, DMSO-*d*6): δ 10.79 (s, 1H, OH_acid_), 8.90 (sa, 1H, NH_acid_), 8.58 (d, *J* = 9.0 Hz, 1H, NH_amide_), 7.90 – 7.76 (m, 4H, H-naphtyl), 7.74 (d, J = 9.0 Hz, 2H, H-3, H-5), 7.60 – 7.40 (m, 3H, H- naphtyl), 7.25 (d, *J* = 9.0 Hz, 2H, H-3, H-5), 4.73 (dd, *J* = 15.0, 9.0 Hz, 1H, H-2’), 3.21 (d, *J* = 6.0 Hz, 2H, H-3’), 2.61 (t, *J* = 9.0 Hz, 2H, H-7), 1.55 (quintet, *J* = 7.5 Hz, 2H, H-8), 1.30 (sextet, *J* = 7.5 Hz, 2H, H-9), 0.89 (t, *J* = 7.5 Hz, 3H, H-10). **^13^C NMR** (75 MHz, DMSO-*d*6): δ 171.2 (CO_acid_), 169.2 (CO_amide_), 149.0 (C-4), 139.1 (C-4’), 136.0 (C-1), 134.9 (C-naphthyl), 134.6 (C-naphthyl), 131.1 (C-2, C-6), 130.9 (C-3, C-5), 130.6 (C-naphthyl), 130.5 (C-naphthyl), 130.4 (C-naphthyl), 129.1 (C-naphthyl), 128.5 (C-naphthyl), 55.8 (C-2’), 40.7 (C-3’), 37.7 (C-7), 36.0 (C-8), 24.8 (C-9), 16.9 (C-10). **HRMS (ESI)**: calculated for [C_24_H_27_N_2_O_3_]^+^ [M+H]^+^ 391.2022; found 391.2030. **HPLC** (Method A, λ: 230 nm): t_R_ 11.10.

**(**S)-4-butyl-N-(3-(4-fluorophenyl)-1-(hydroxyamino)-1-oxopropan-2-yl)benzamide **(YSL-116).**

Salmon color solid was obtained (0.102 g, 43%). **^1^H NMR** (300 MHz, DMSO-*d*6): δ 10.76 (s, 1H, OH_acid_), 8.89 (s, 1H, NH_acid_), 8.52 (d, *J* = 8.6 Hz, 1H, NH_amide_), 7.74 (d, *J* = 8.0 Hz, 2H, H-2, H-6), 7.36 (dd, *J* = 8.0 Hz, ^4^*J*_H-F_ = 6.0 Hz, 2H, H-5’, H-9’), 7.26 (d, *J* = 7.9 Hz, 2H, H-3, H-5), 7.09 (dd, *J* = 8.8 Hz, ^3^*J*_H-F_ = 8.8 Hz, 2H, H-6’, H-8’), 4.58 (dd, *J* = 15.3, 7.9 Hz, 1H, H-2’), 3.02 (d, *J* = 7.2 Hz, 2H, H-3’), 2.63 (t, *J* = 7.6 Hz, 2H, H-7), 1.57 (quintet, *J* = 7.6 Hz, 2H, H-8), 1.31 (sextet, *J* = 7.2 Hz, 2H, H-9), 0.90 (t, *J* = 7.3 Hz, 3H, H-10). **^13^C NMR** (75 MHz, DMSO-*d*6): δ 168.5 (CO_acid_), 166.6 (CO_amide_), 161.4 (C-7’, ^1^*J*_C-F_ = 144 Hz), 146.4 (C-4), 134.9 (C-1), 131.9 (C-4’), 131.4 (C-5’, C-9’, ^3^*J*_C-F_ = 5.3 Hz), 128.5 (C-2, C-6), 128.0 (C-3, C-5), 115.3 (C-6’, C-8’, ^2^*J*_C-F_ = 12.8 Hz), 53.2 (C-2’), 37.0 (C-3’), 35.1 (C-7), 33.4 (C-8), 22.2 (C-9), 14.2 (C-10). **HRMS (ESI)**: calculated for [C_20_H_24_N_2_O_3_F]^+^ [M+H]^+^ 359.1771; found 359.1773. **HPLC** (Method A, λ: 240 nm): t_R_ 9.48.

(S)-4-butyl-N-(1-(hydroxyamino)-3-(4-iodophenyl)-1-oxopropan-2-yl)benzamide **(YSL-121).**

White solid was obtained (0.044 g, 66%). **^1^H NMR** (300 MHz, DMSO-*d*6): δ 10.76 (s, 1H, OH_acid_), 8.88 (s, 1H, NH_acid_), 8.52 (d, *J* = 9.0 Hz, 1H, NH_amide_), 7.74 (d, *J* = 9.0 Hz, 2H), 7.63 (d, *J* = 9.0 Hz, 2H), 7.27 (d, *J* = 9.0 Hz, 2H), 7.15 (d, *J* = 9.0 Hz, 2H), 4.58 (dd, *J* = 15.0, 9.0 Hz, 1H, H-2’), 2.98 (d, *J* = 6.0 Hz, 2H, H-3’), 2.63 (t, *J* = 7.5 Hz, 2H, H-7), 1.57 (quintet, *J* = 7.5 Hz, 2H, H-8), 1.31 (sextet, *J* = 7.5 Hz, 2H, H-9), 0.91 (t, *J* = 7.5 Hz, 3H, H-10). **^13^C NMR** (75 MHz, DMSO-*d*6): δ 171.0 (CO_acid_), 169.2 (CO_amide_), 149.1 (C-4), 141.2 (C-6’, C-8’), 139.9 (C-4’), 134.7 (C-1), 134.5 (C-5’, C-9’), 131.1 (C-2, C-6), 130.6 (C-3, C-5), 95.4 (C-7’), 55.6 (C-2’), 40.0 (C-3’), 37.7 (C-7), 36.0 (C-8), 24.8 (C-9), 16.9 (C-10). **HRMS (ESI)**: calculated for [C_20_H_24_N_2_O_3_I]^+^ [M+H]^+^ 467.0832; found 467.0838. **HPLC** (Method A, λ: 230 nm): t_R_ 11.28.

**(**S)-4-butyl-N-(1-(hydroxyamino)-3-(4-nitrophenyl)-1-oxopropan-2-yl)benzamide **(YSL-125).**

White solid was obtained (0.03 g, 60%). **^1^H NMR** (300 MHz, DMSO-*d*6): δ 8.61 (d, *J* = 9.0 Hz, 1H, NH_amide_), 8.15 (d, *J* = 9.0 Hz, 2H, H-6’, H-8’), 7.72 (d, *J* = 6.0 Hz, 2H, H-2, H-6), 7.60 (d, *J* = 9.0 Hz, 2H, H-5’, H-9’), 7.25 (d, *J* = 6.0 Hz, 2H, H-3, H-5), 4.67 (ddd, *J* = 9.0, 9.0, 6.0 Hz, 1H, H-2’), 3.18 (dd, *J* = 6.0, 3.0 Hz, 1H, H-3’), 2.61 (t, *J* = 9.0 Hz, 2H, H-7), 1.54 (m, *J* = 9.0 Hz, 2H, H-8), 1.29 (sextet, *J* = 9.0 Hz, 2H, H-9), 0.89 (t, *J* = 6.0 Hz, 3H, H-10).**^13^C NMR** (75 MHz, DMSO-d6): δ 170.7 (CO_acid_), 169.2 (CO_amide_), 149.8 (C-7’), 149.3 (C-4’), 149.1 (C-4), 134.4 (C-1), 133.6 (C-5’, C-9’), 131.2 (C-2, C-6), 130.6 (C-3, C-5), 126.3 (C-6’, C-8’), 55.3 (C-2’), 40.3 (C-3’), 37.7 (C-7), 36.0 (C-8), 24.8 (C-9), 16.9 (C-10). **HRMS (ESI)**: calculated for [C_20_H_24_N_3_O_5_]^+^ [M+H]^+^ 386.1716; found 386.1714. **HPLC** (Method A, λ: 254 nm): t_R_ 9.34.

(S)-4-butyl-N-(2-(hydroxyamino)-2-oxo-1-phenylethyl)benzamide **(YSL-129).**

White solid was obtained (0.004 g, 8.6%). **^1^H NMR** (300 MHz, DMSO-*d*6): δ 11.02 (sa, 1H, OH_acid_), 8.98 (s, 1H, NH_acid_), 8.72 (d, *J* = 9.0 Hz, 1H, NH_amide_), 7.83 (d, *J* = 6.0 Hz, 2H, H-2, H-6), 7.50 (d, *J* = 6.0 Hz, 2H, H-3, H-5), 7.40-719 (m, 5H, H-5’, H-6’, H-7’, H-8’, H-9’), 5.60 (d, *J* = 6.0 Hz, 1H, H-1’), 2.62 (t, *J* = 7.5 Hz, 2H, H-7), 1.56 (quintet, *J* = 7.5 Hz, 2H, H-8), 1.30 (sextet, *J* = 7.5 Hz, 2H, H-9), 0.89 (t, *J* = 7.5 Hz, 3H, H-10). **^13^C NMR** (75 MHz, DMSO-*d*6): δ 169.8 (CO_acid_), 169.2 (CO_amide_), 149.2 (C-4), 141.6 (C-3’), 134.4 (C-1), 131.3 (C-5’, C-7’), 131.2 (C-2, C-6), 130.9 (C-3, C-5), 130.7 (C-4’, C-8’), 130.5 (C-6’), 57.7 (C-2’), 37.7 (C-7), 36.0 (C-8), 24.8 (C-9), 16.9 (C-10). **HRMS (ESI)**: calculated for [C_19_H_23_N_2_O_3_]^+^ [M+H]^+^ 327.1709; found 327.1707. **HPLC** (Method A, λ: 240 nm): t_R_ 9.11.

**^1^H-NMR and ^13^C-NMR spectra**

**GH38**

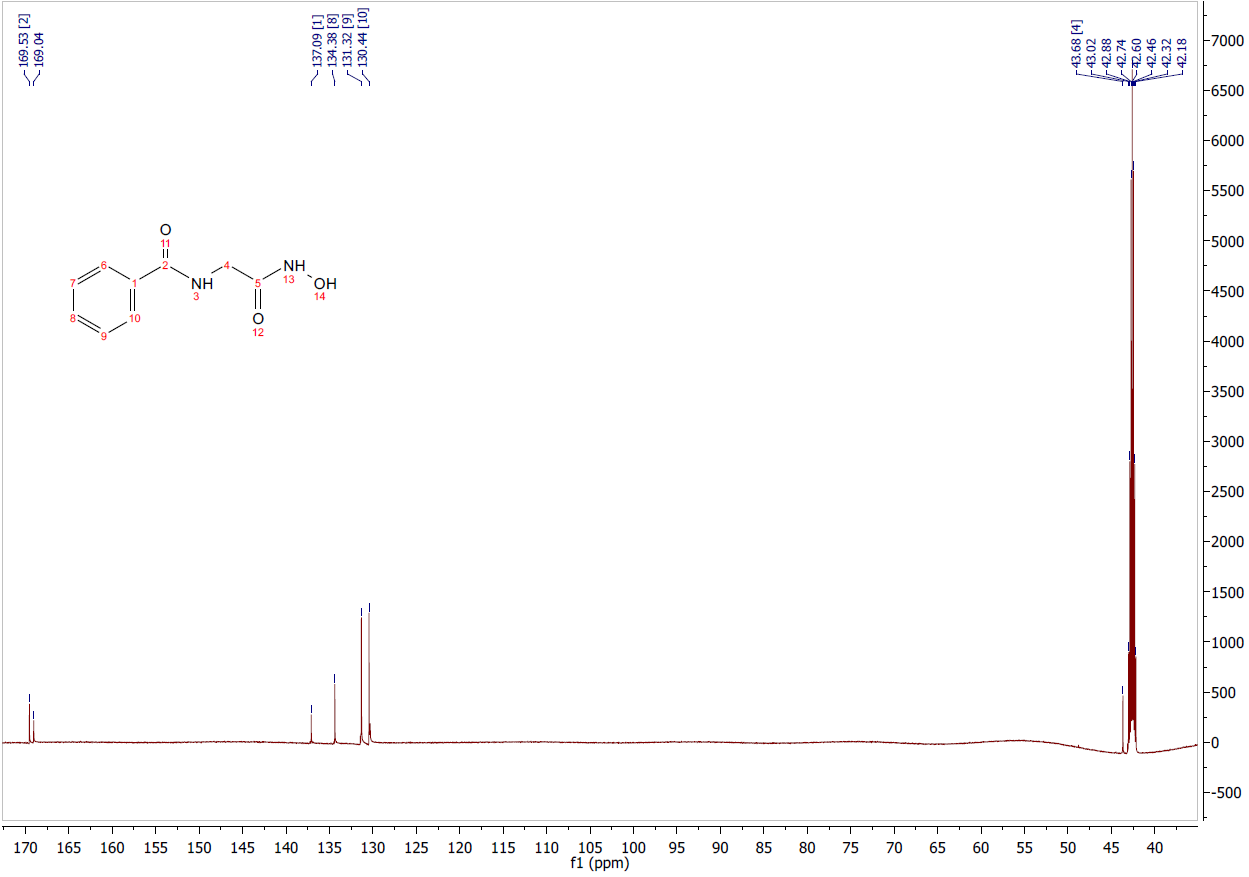


(^13^C-NMR, CDCl3, 300 MHz)


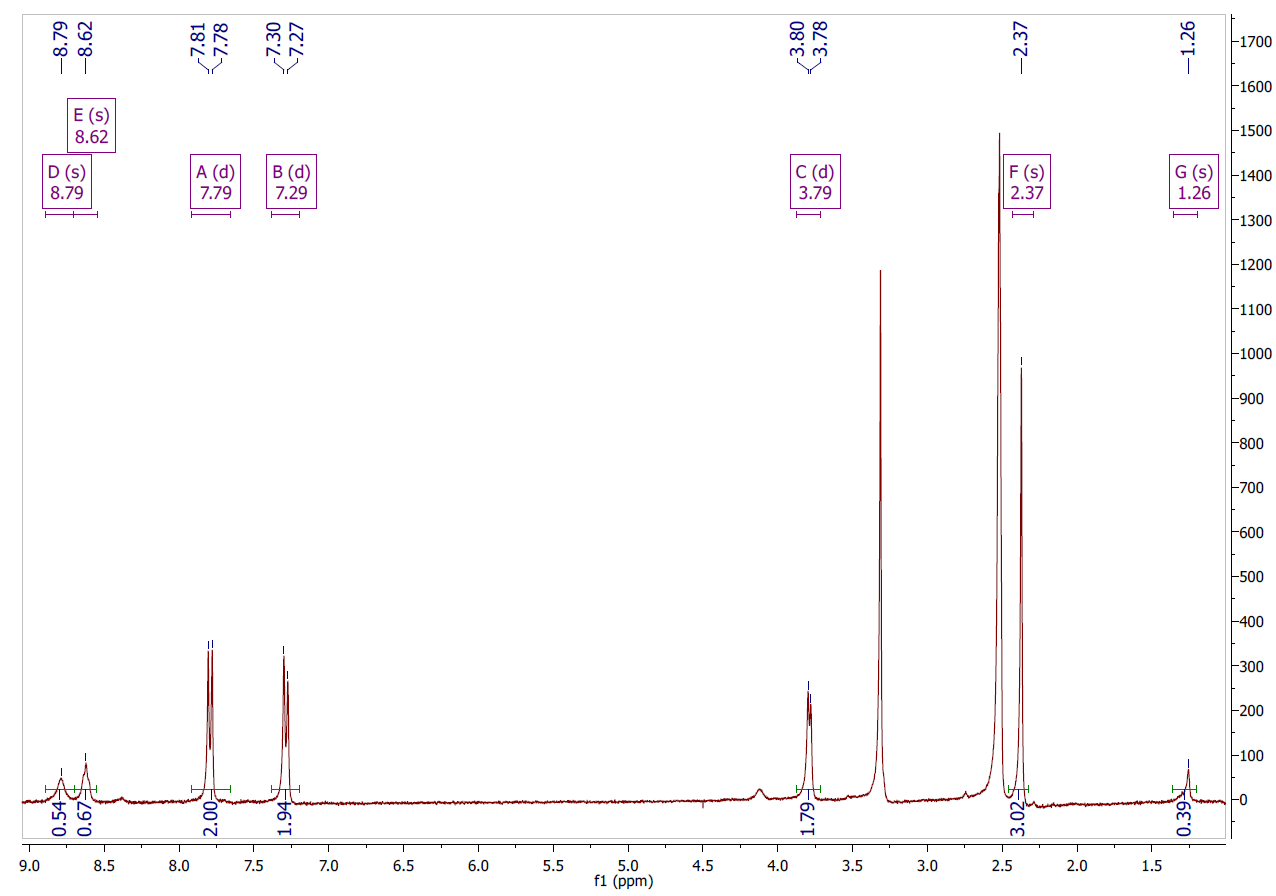


(^1^H-NMR, CDCl3, 300 MHz)

**GH18**

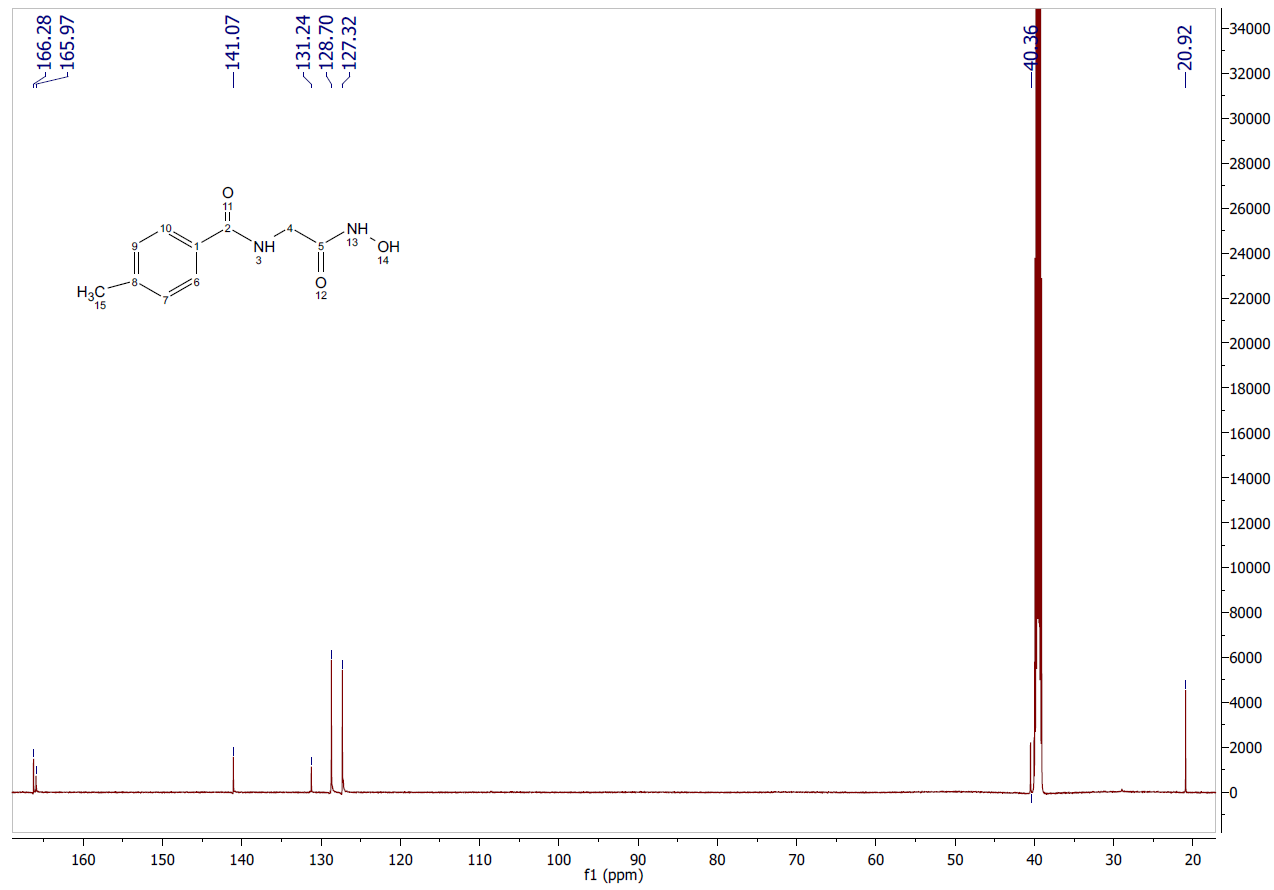


(^13^C-NMR, CDCl3, 300 MHz)


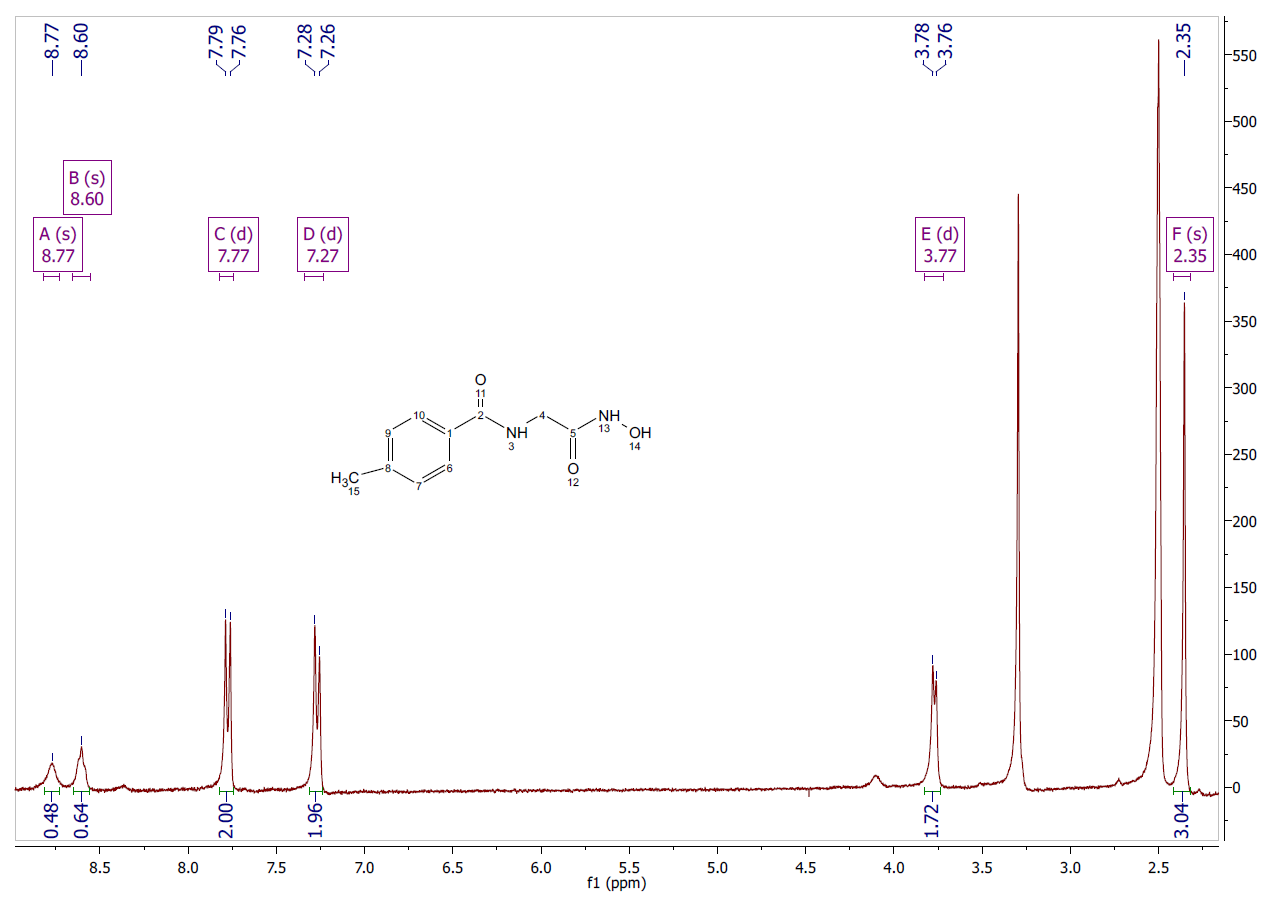


(^1^H-NMR, CDCl3, 300 MHz)

**GH27**

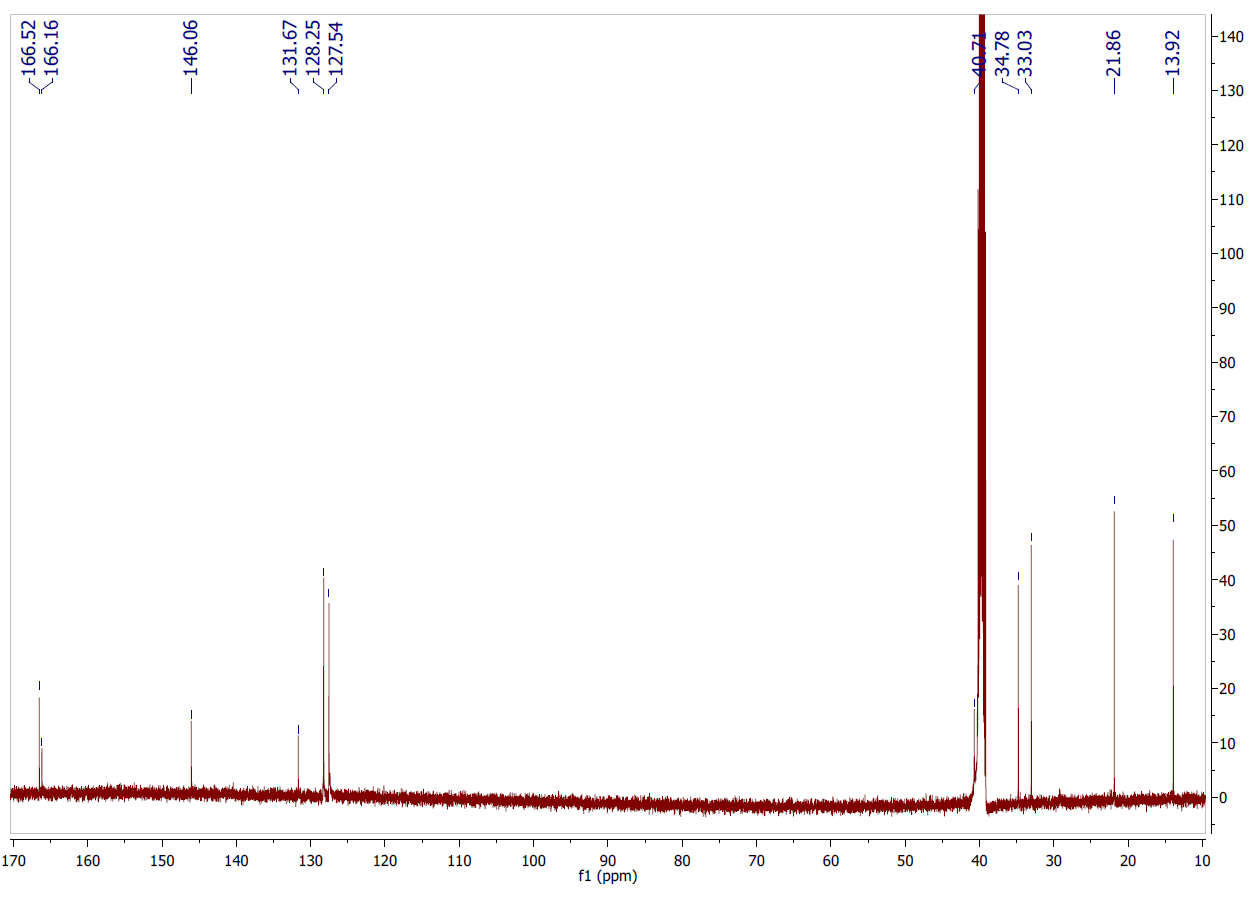


(^13^C-NMR, CDCl3, 300 MHz)


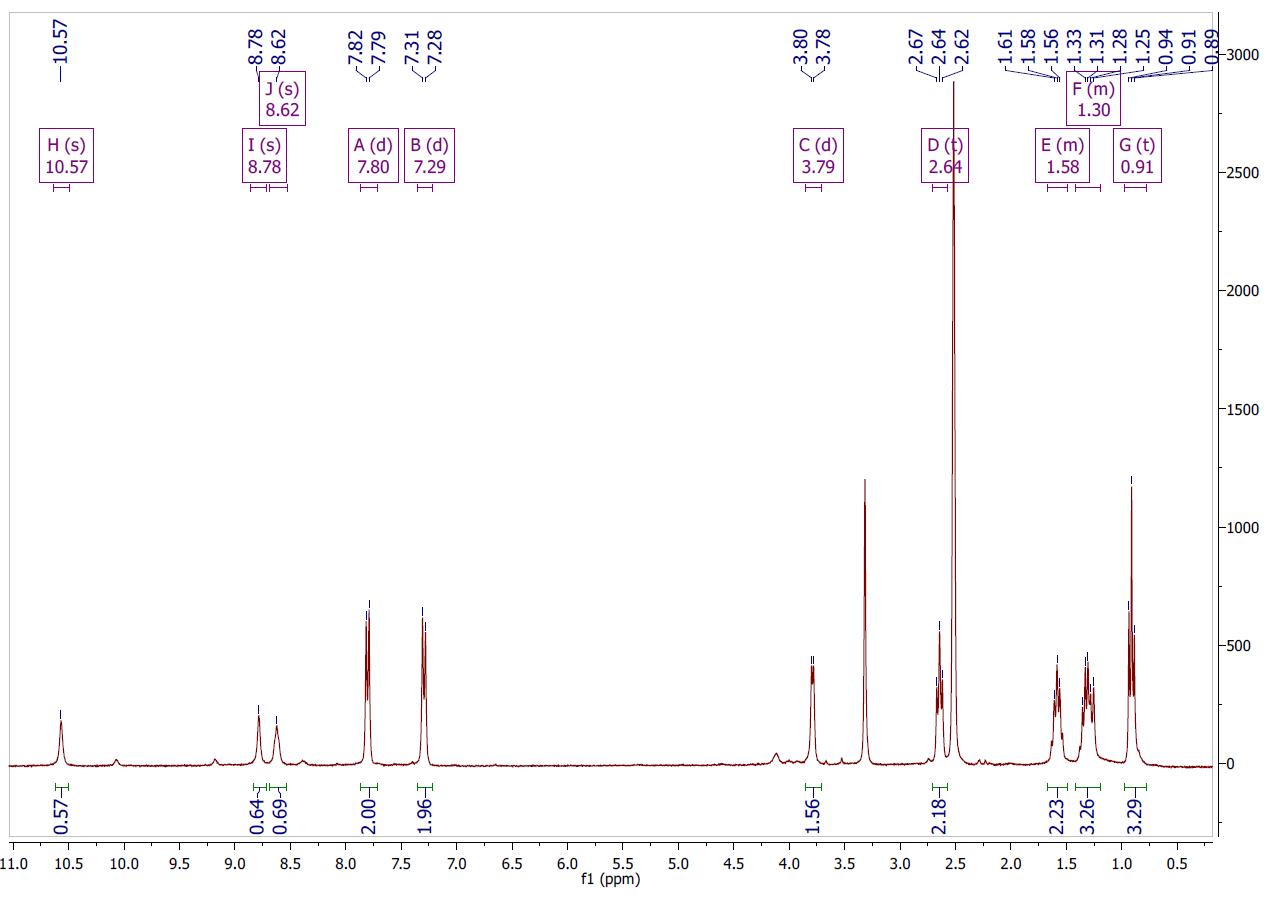


(^1^H-NMR, CDCl3, 300 MHz)

**FH38**

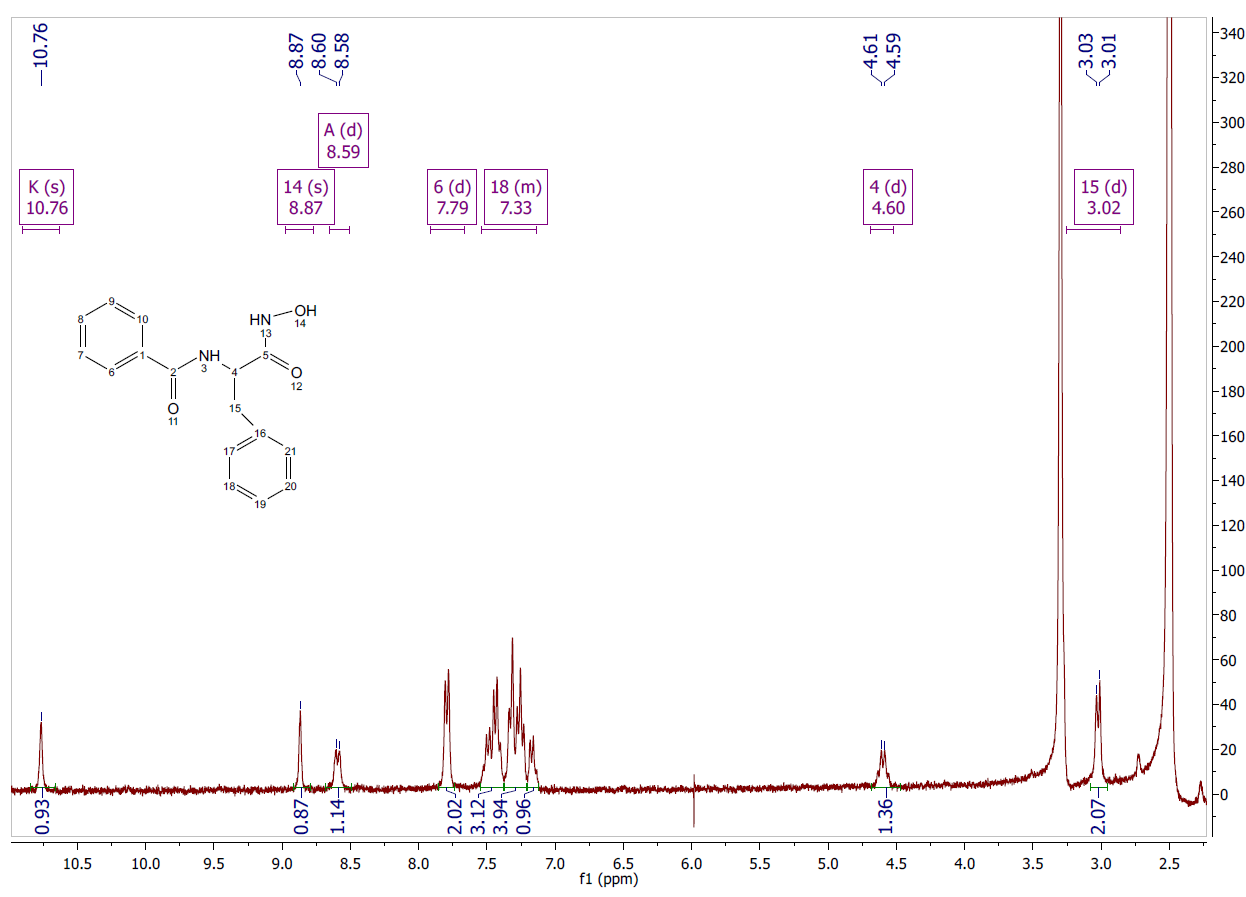


(^1^H-NMR, CDCl3, 300 MHz)

**FH18**

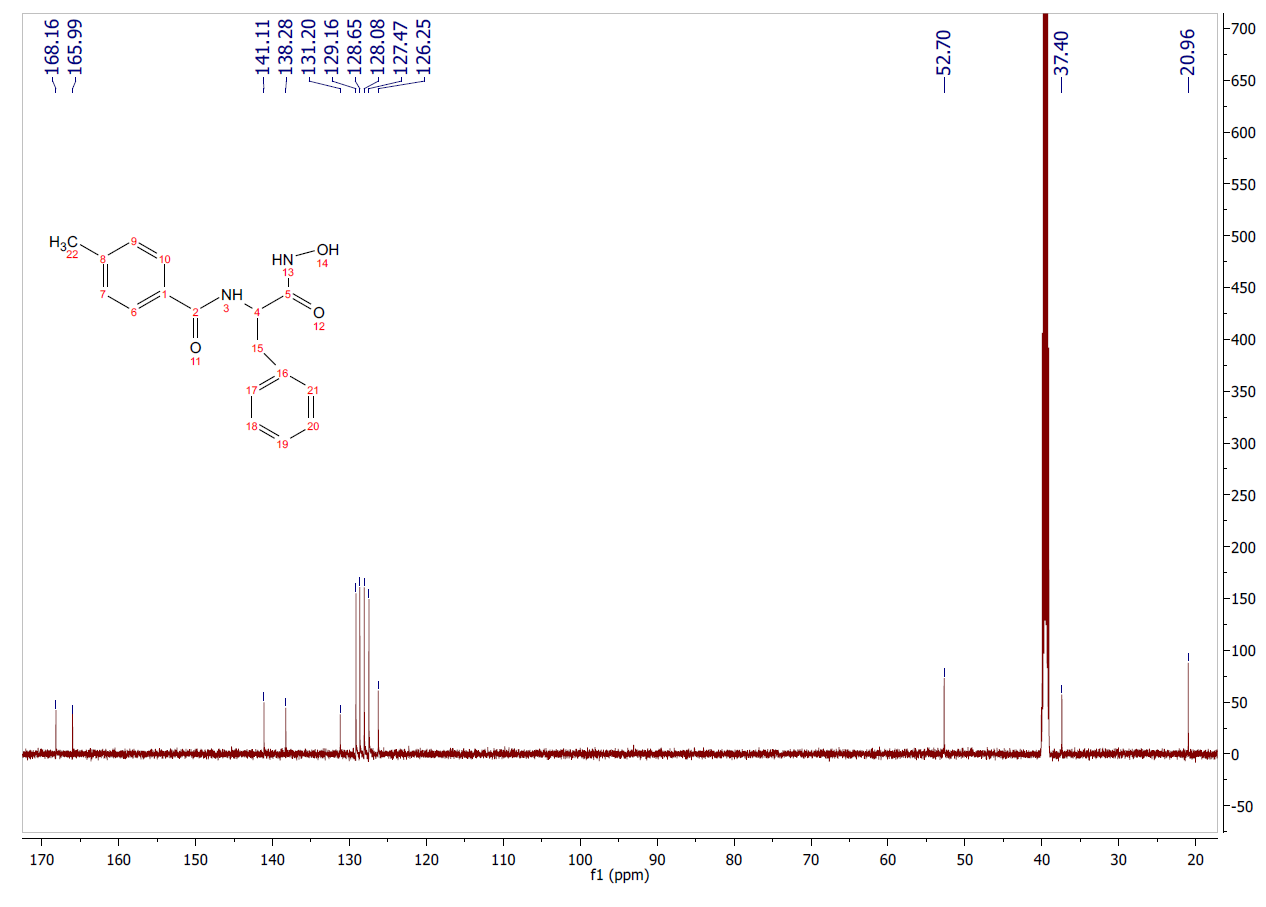


(^13^C-NMR, CDCl3, 300 MHz)


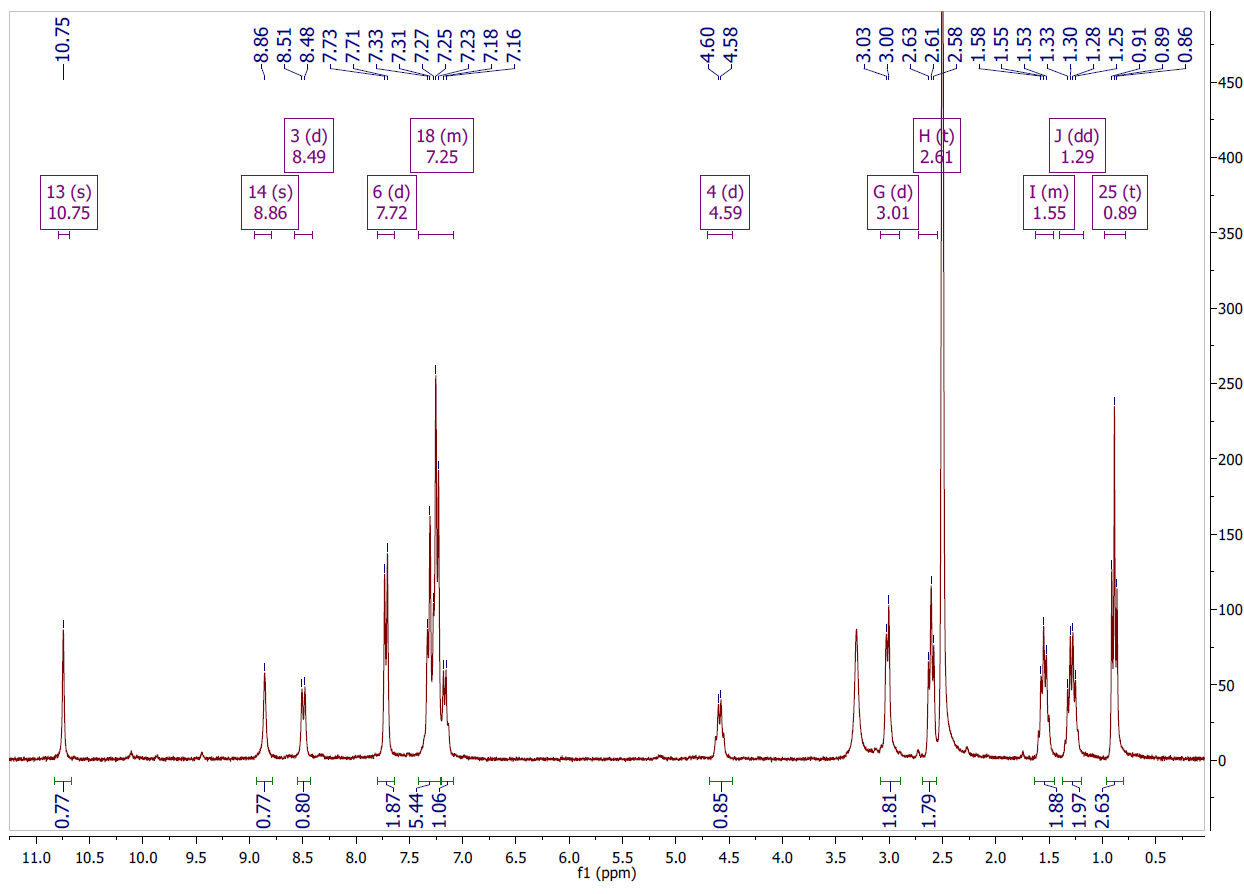


(^1^H-NMR, CDCl3, 300 MHz)

**FH27**

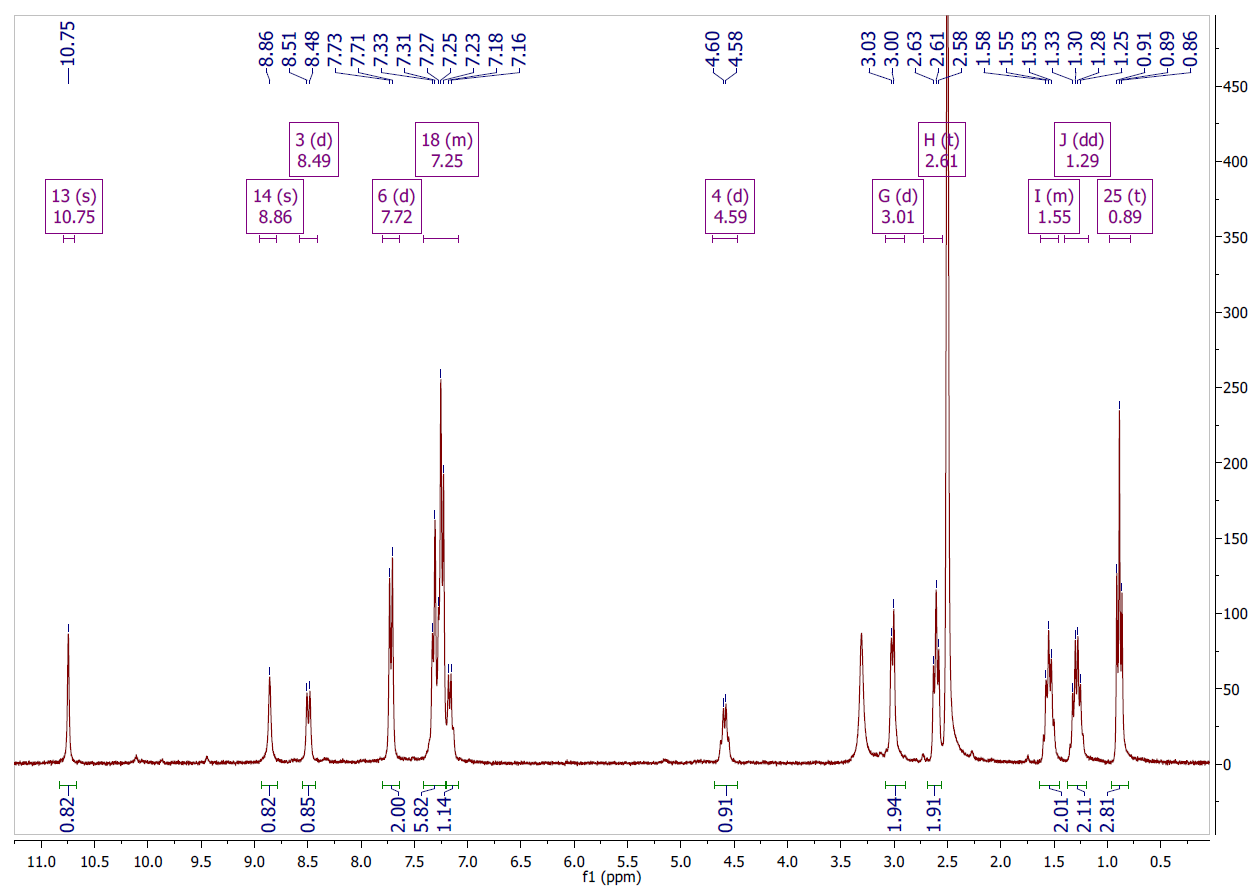


(^13^C-NMR, CDCl3, 300 MHz)


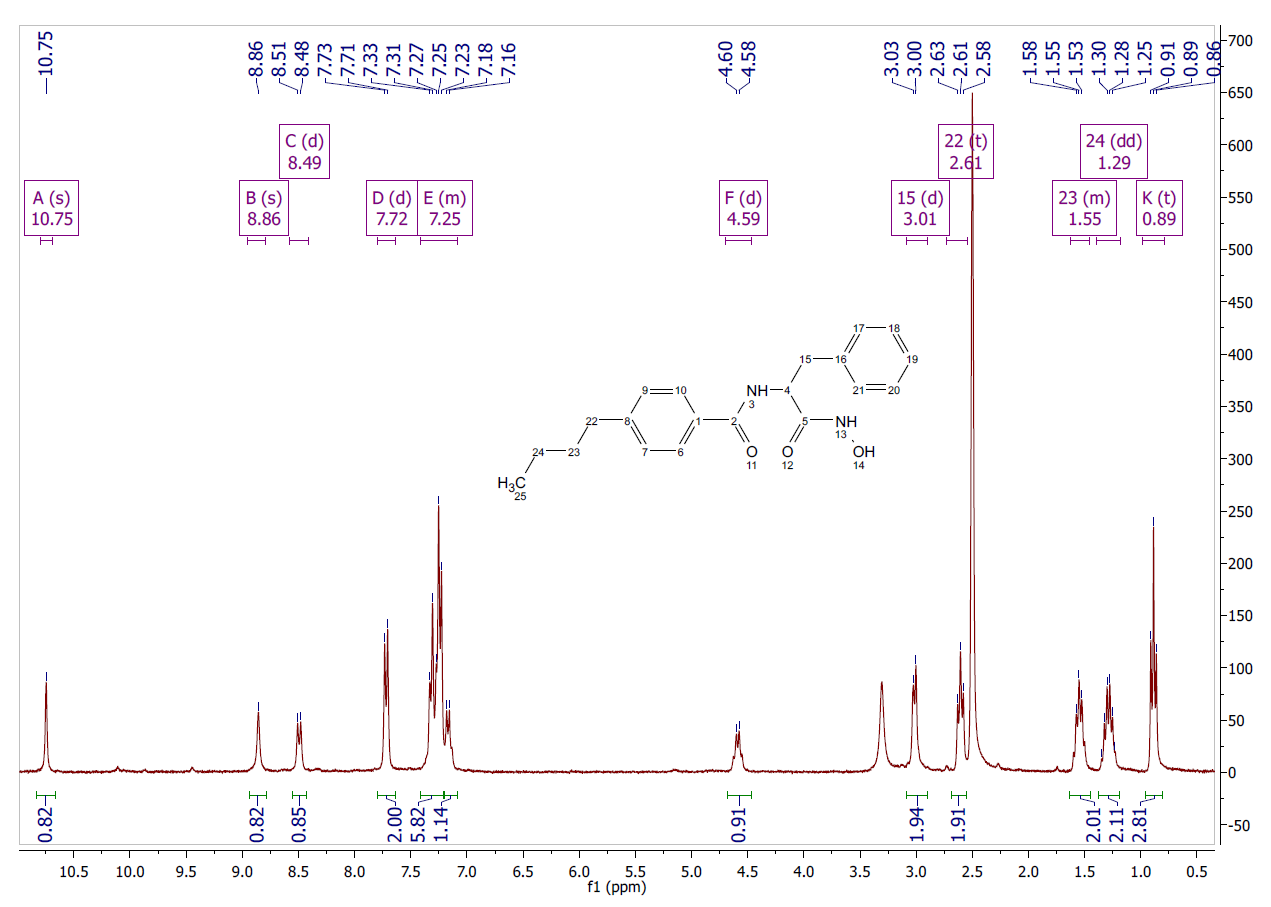


(^1^H-NMR, CDCl3, 300 MHz)

**FH37**

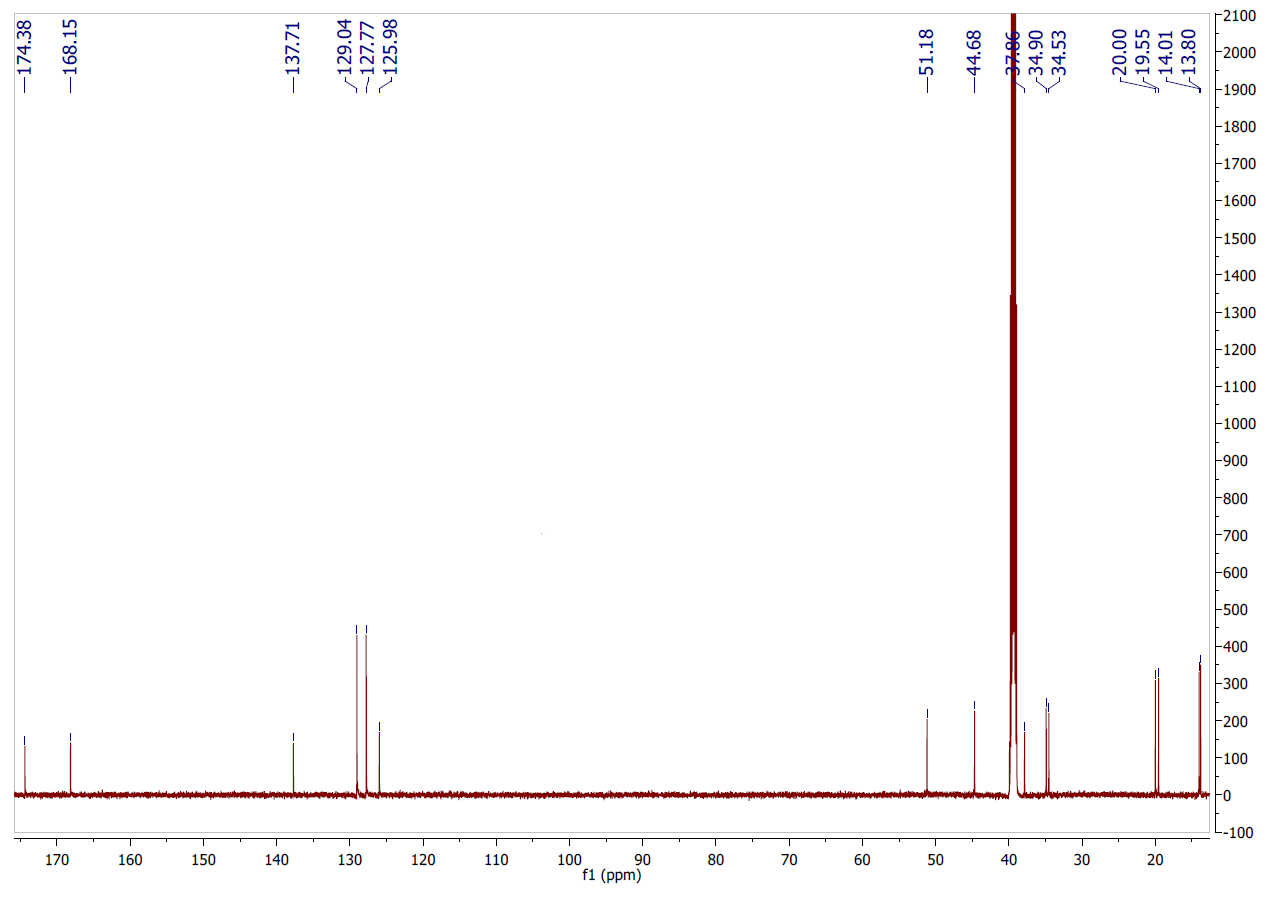


(^13^C-NMR, CDCl3, 300 MHz)


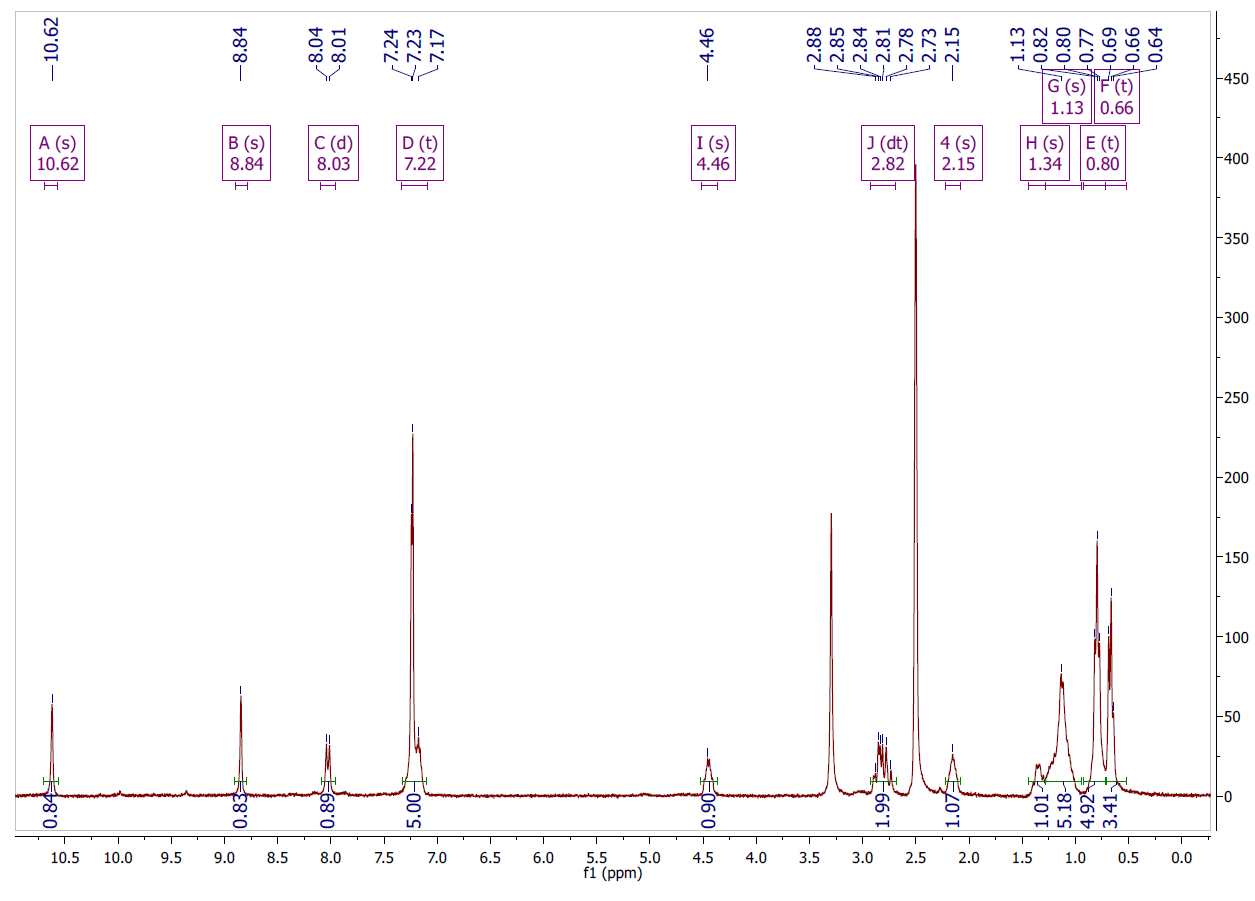


(^1^H-NMR, CDCl3, 300 MHz)

**YSL-99**

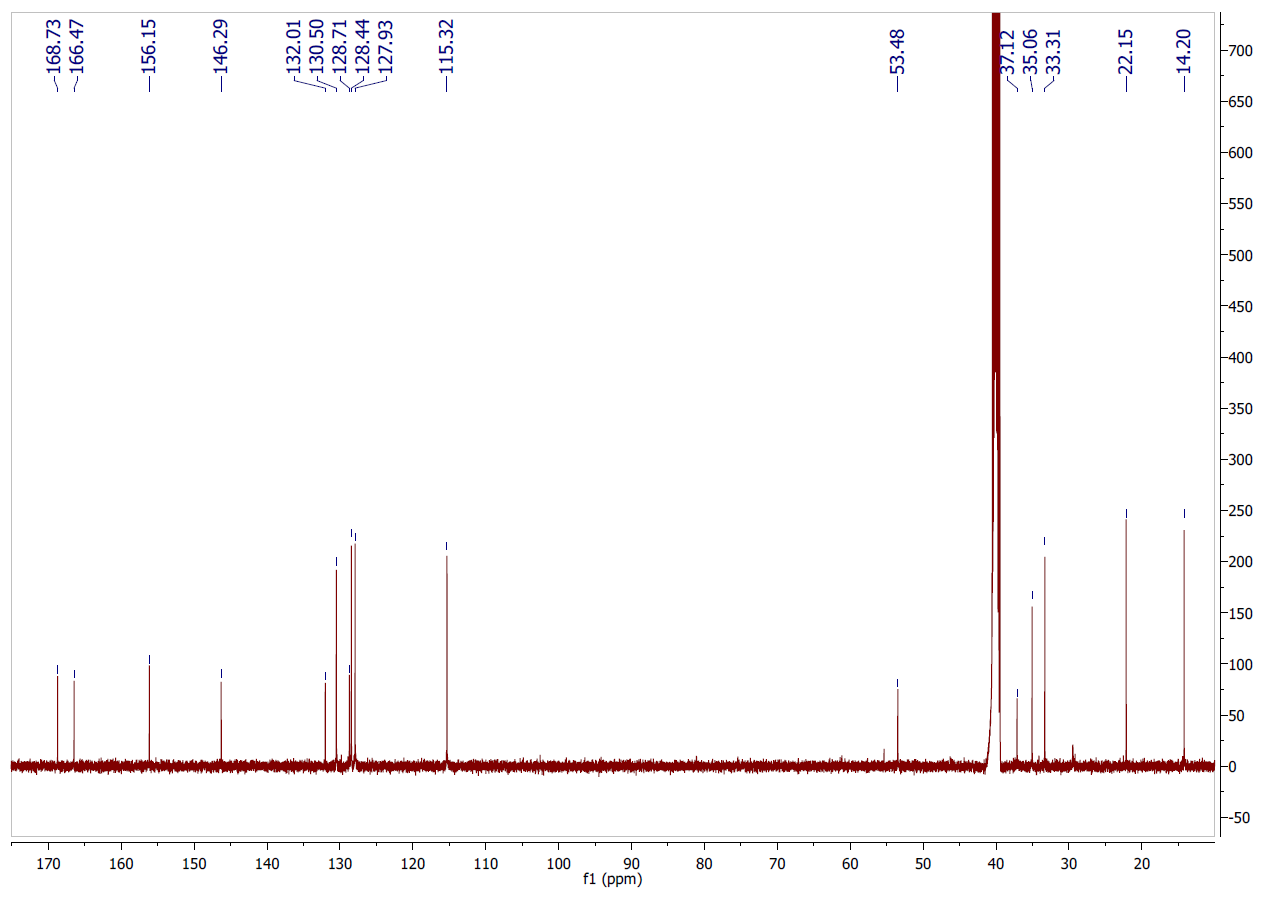


(^13^C-NMR, CDCl3, 300 MHz)


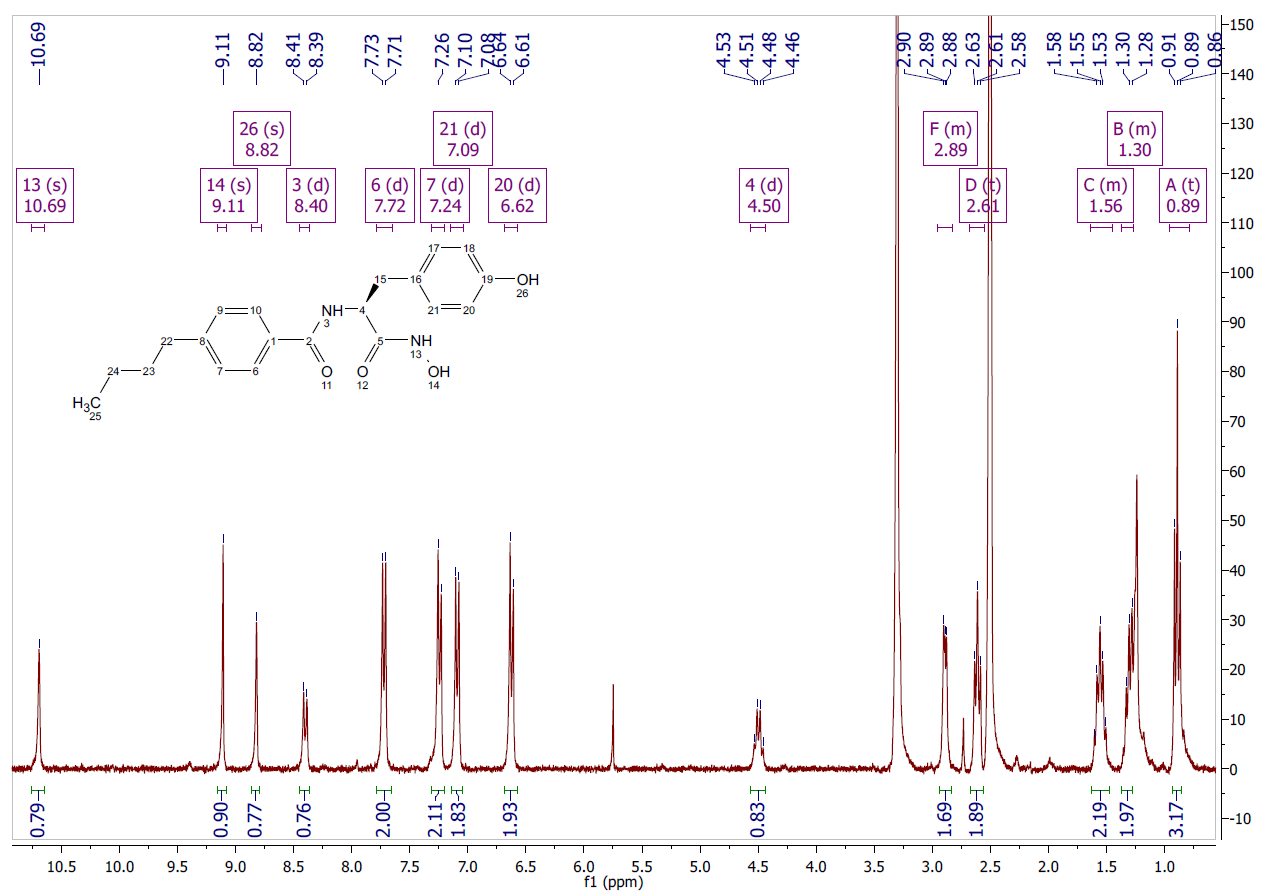


(^1^H-NMR, CDCl3, 300 MHz)

**YSL-106**

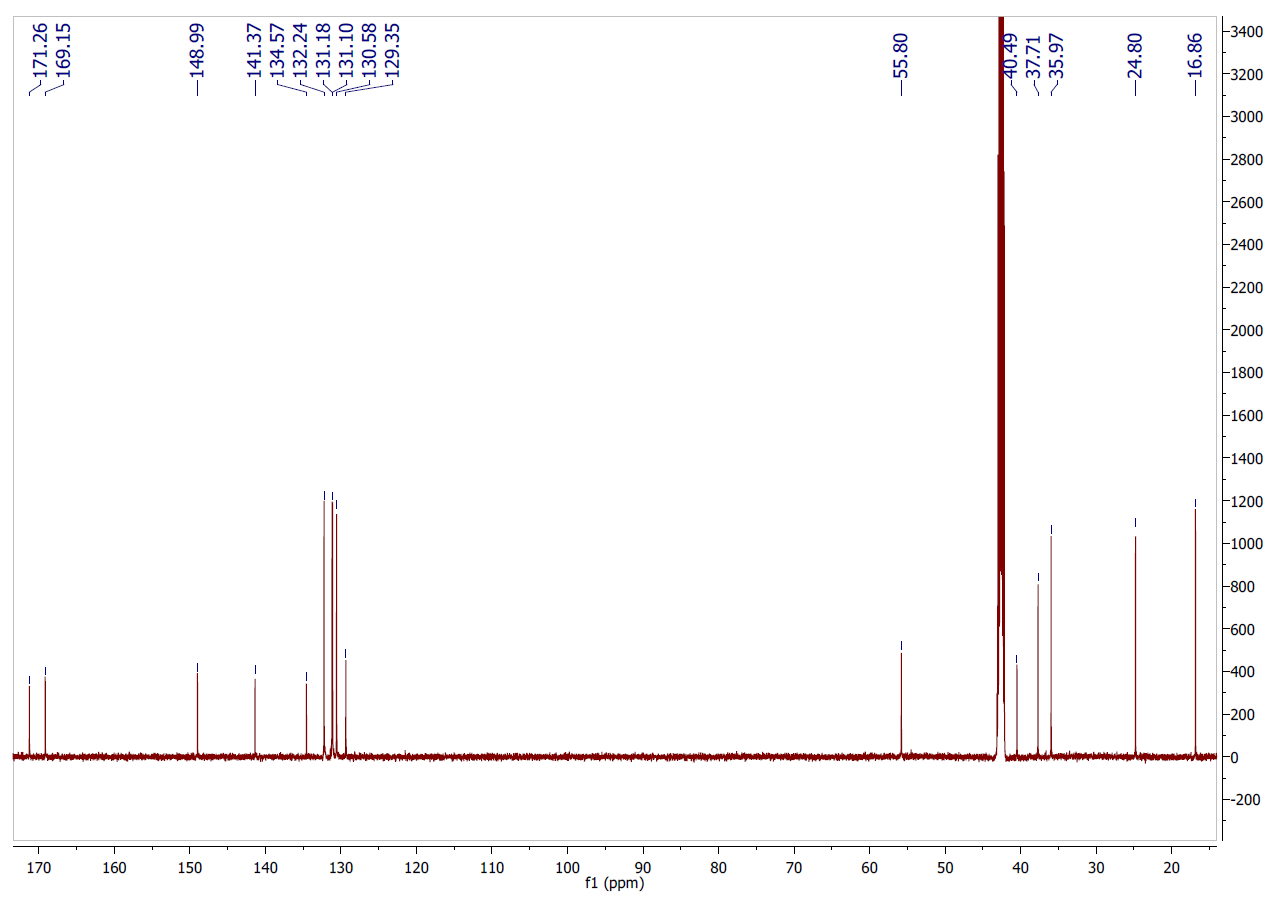


(^13^C-NMR, CDCl3, 300 MHz)


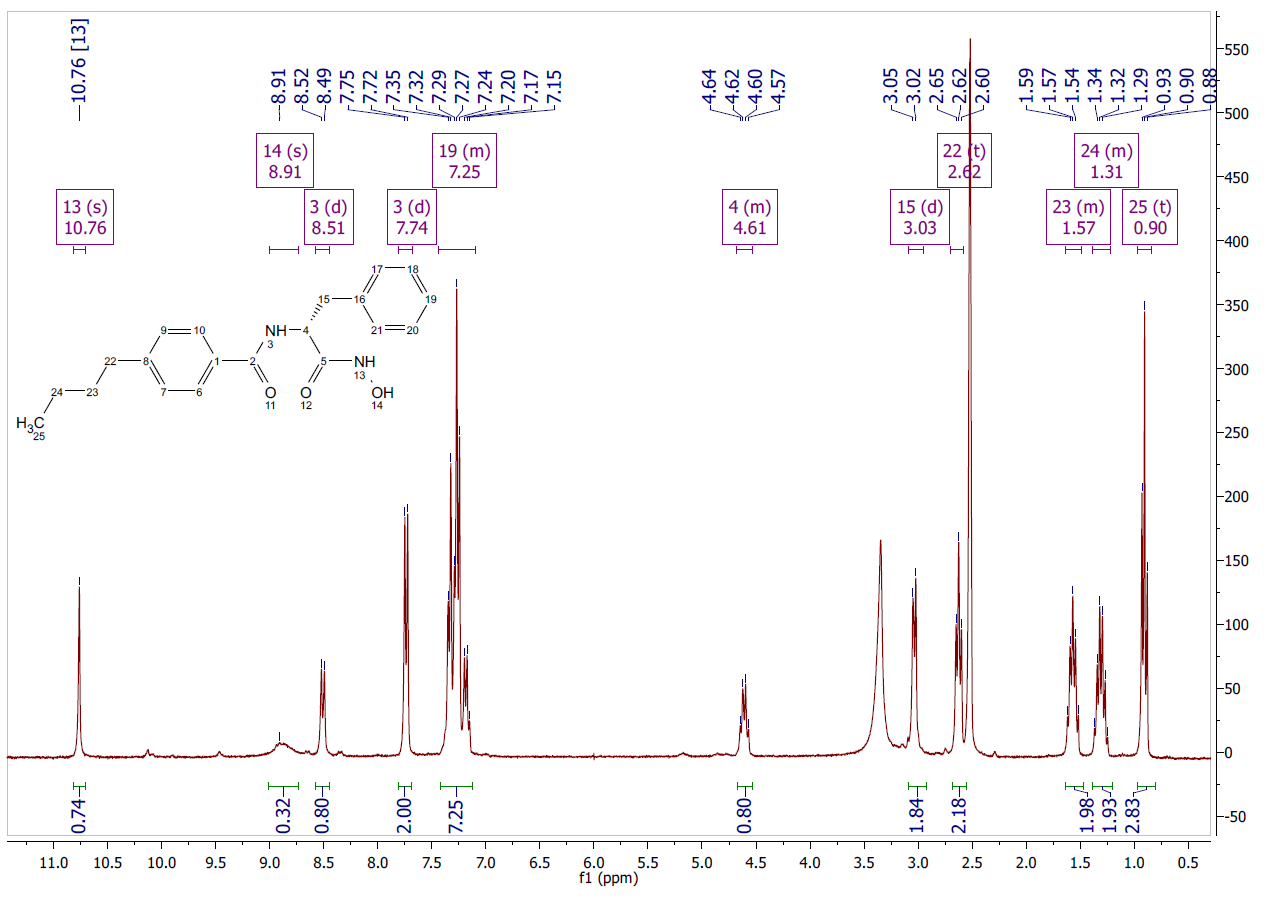


(^1^H-NMR, CDCl3, 300 MHz)

**YSL-109**

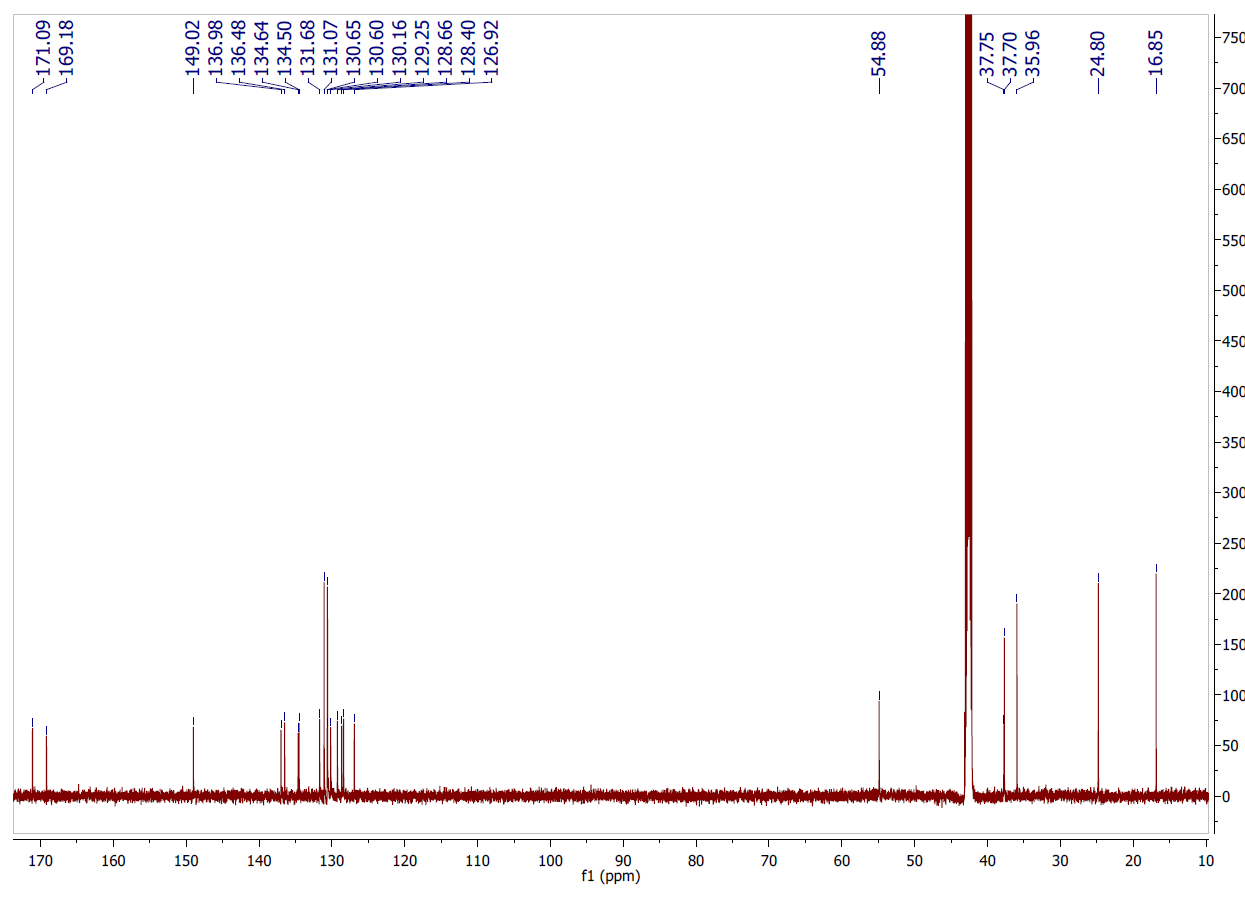


(^13^C-NMR, CDCl3, 300 MHz)


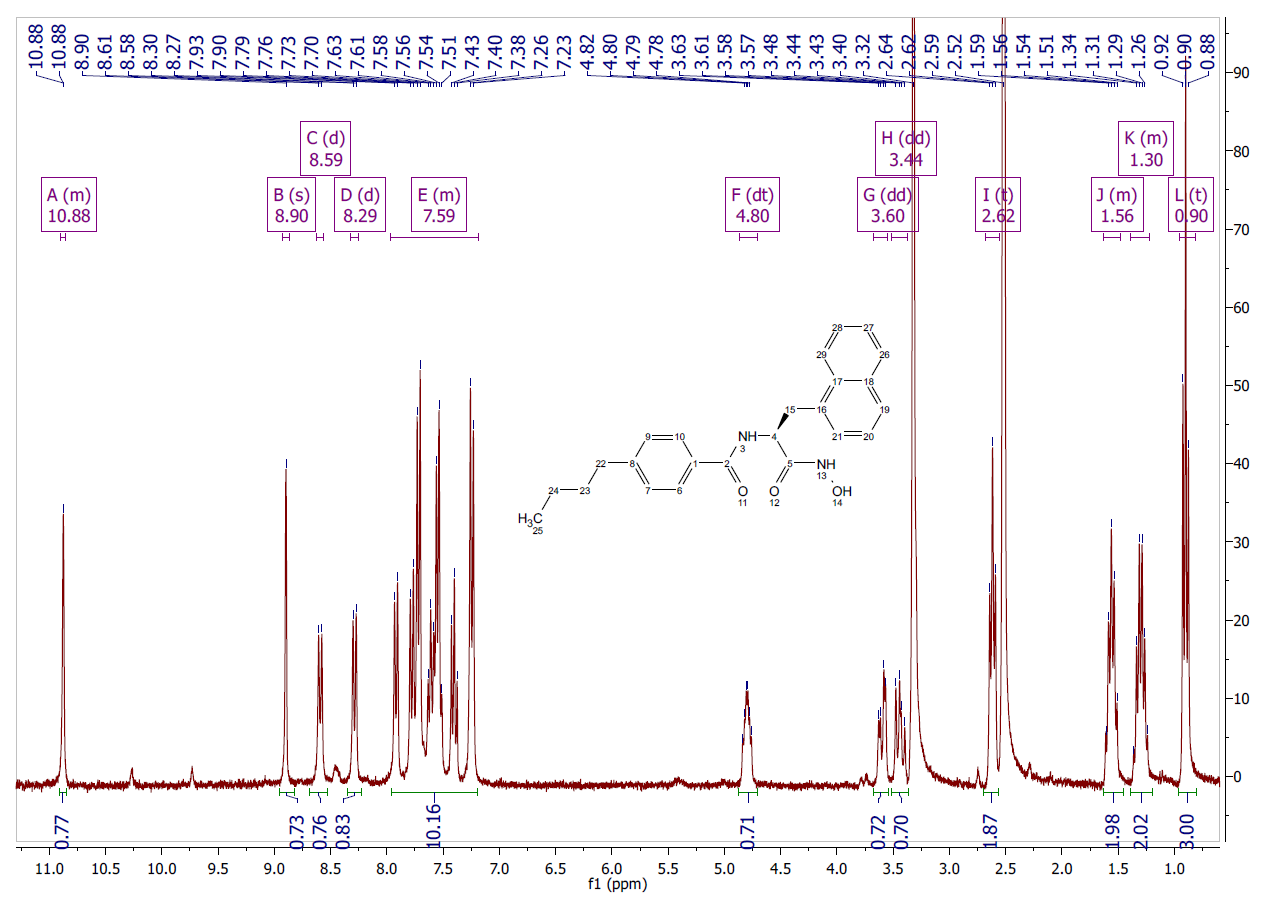


(^1^H-NMR, CDCl3, 300 MHz)

**YSL-112**

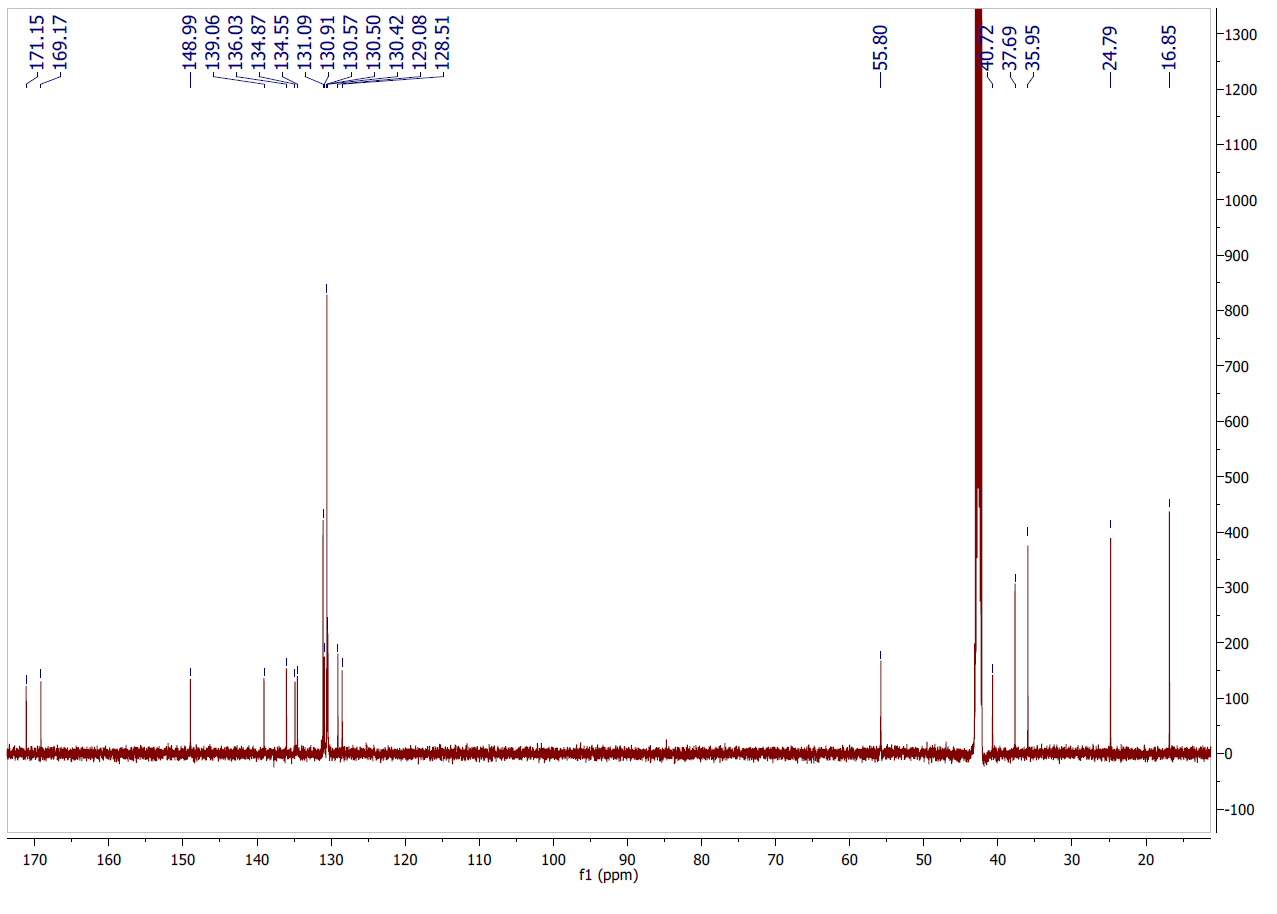


(^13^C-NMR, CDCl3, 300 MHz)


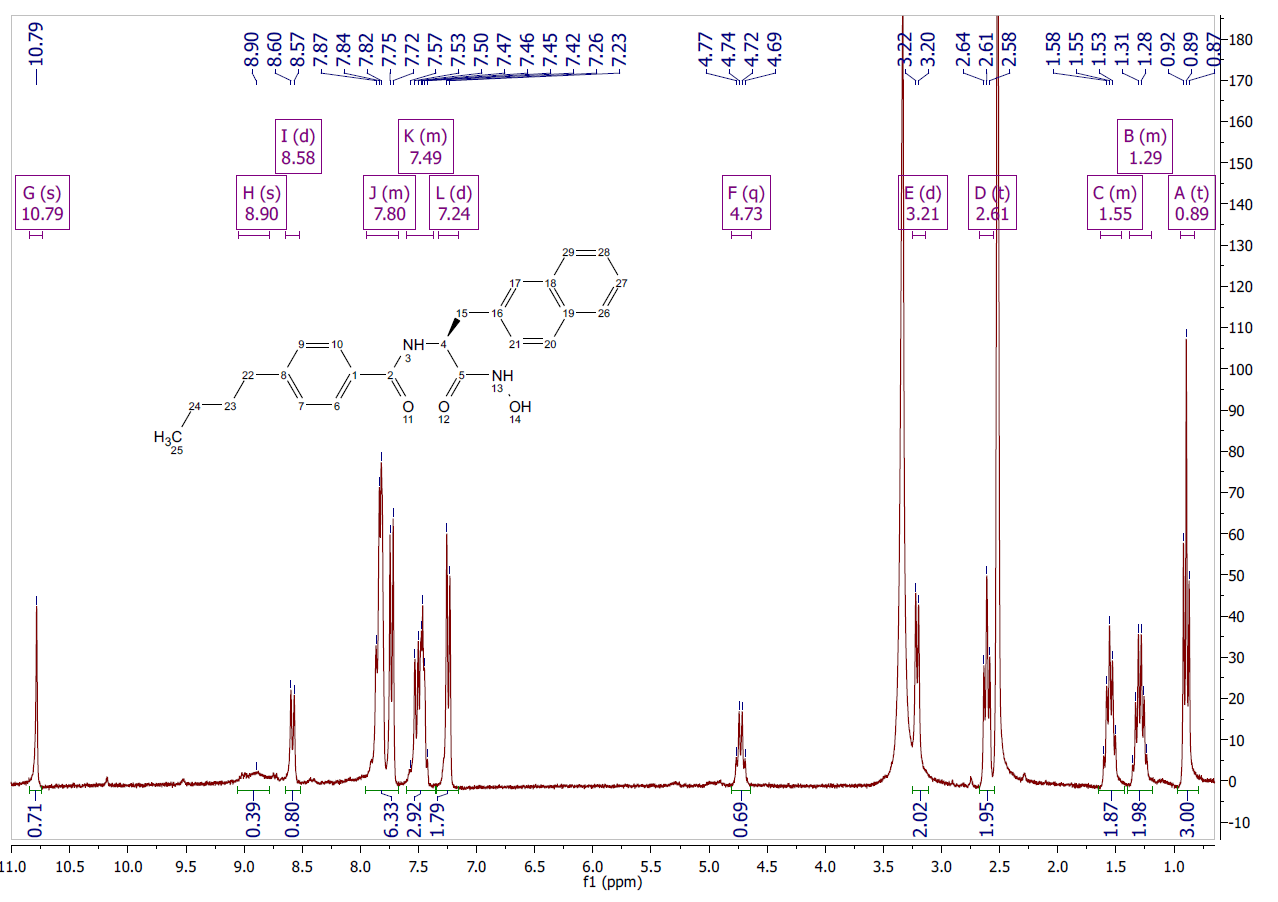


(^1^H-NMR, CDCl3, 300 MHz)

**YSL-116**

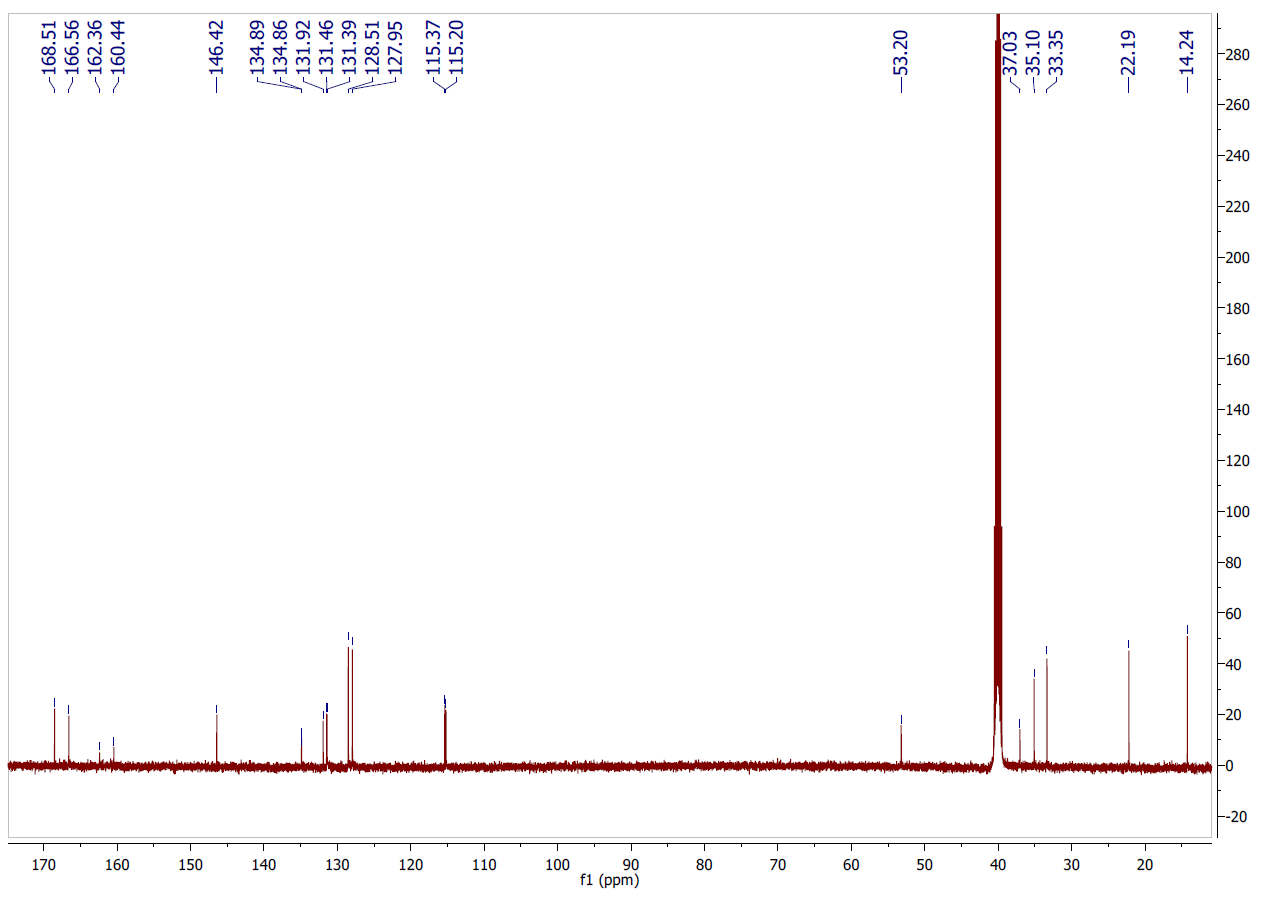


(^13^C-NMR, CDCl3, 300 MHz)


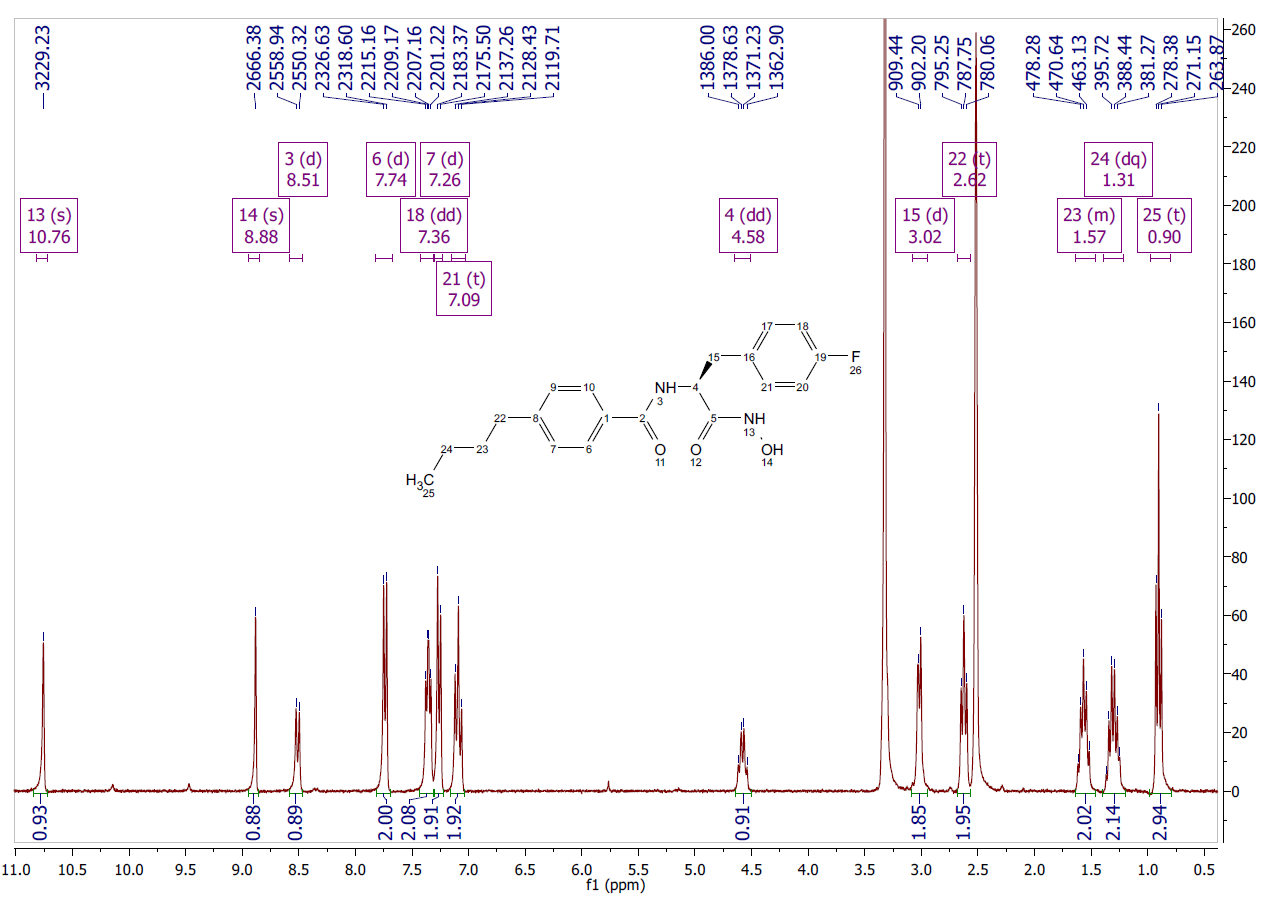


(^1^H-NMR, CDCl3, 300 MHz)

**YSL-121**

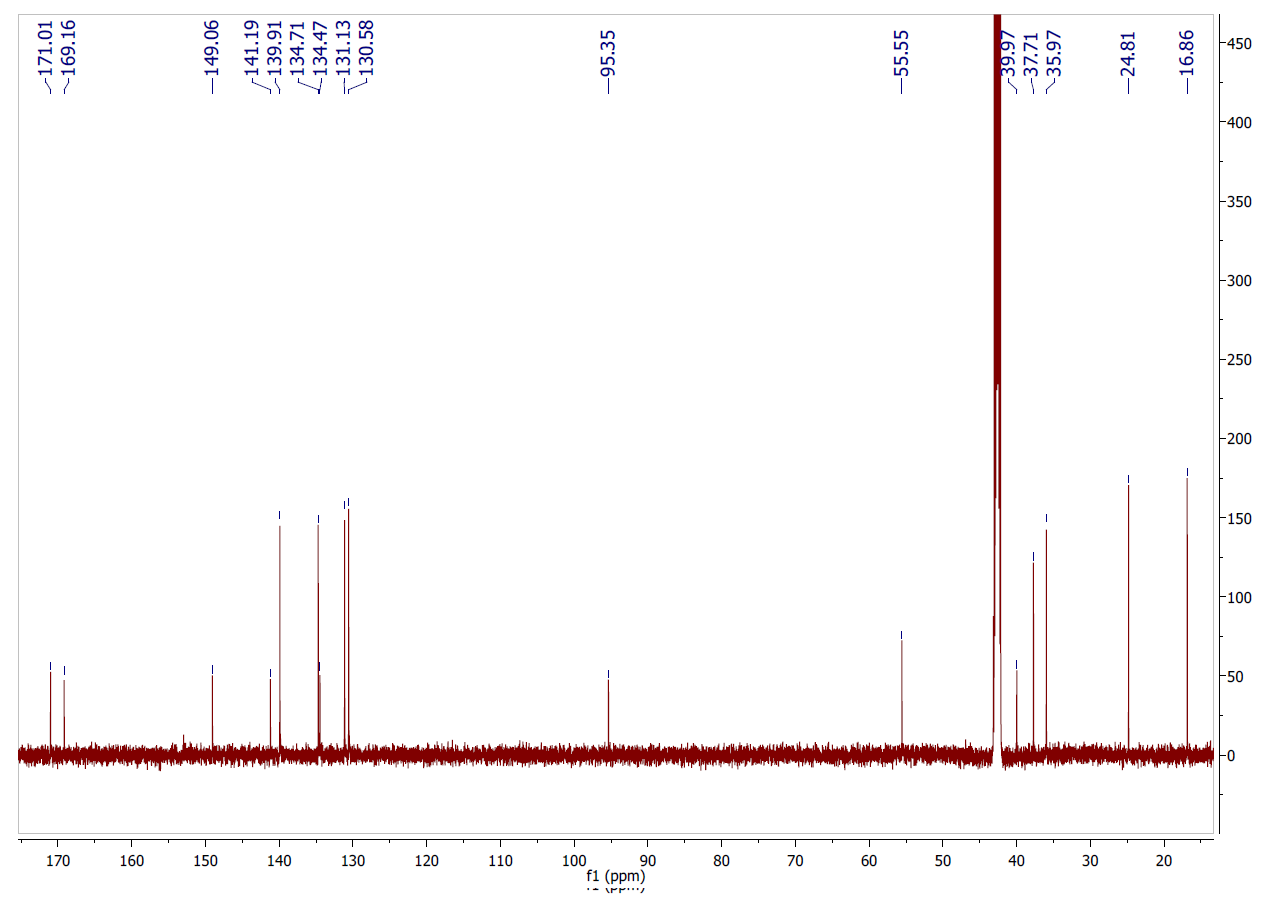


(^13^C-NMR, CDCl3, 300 MHz)


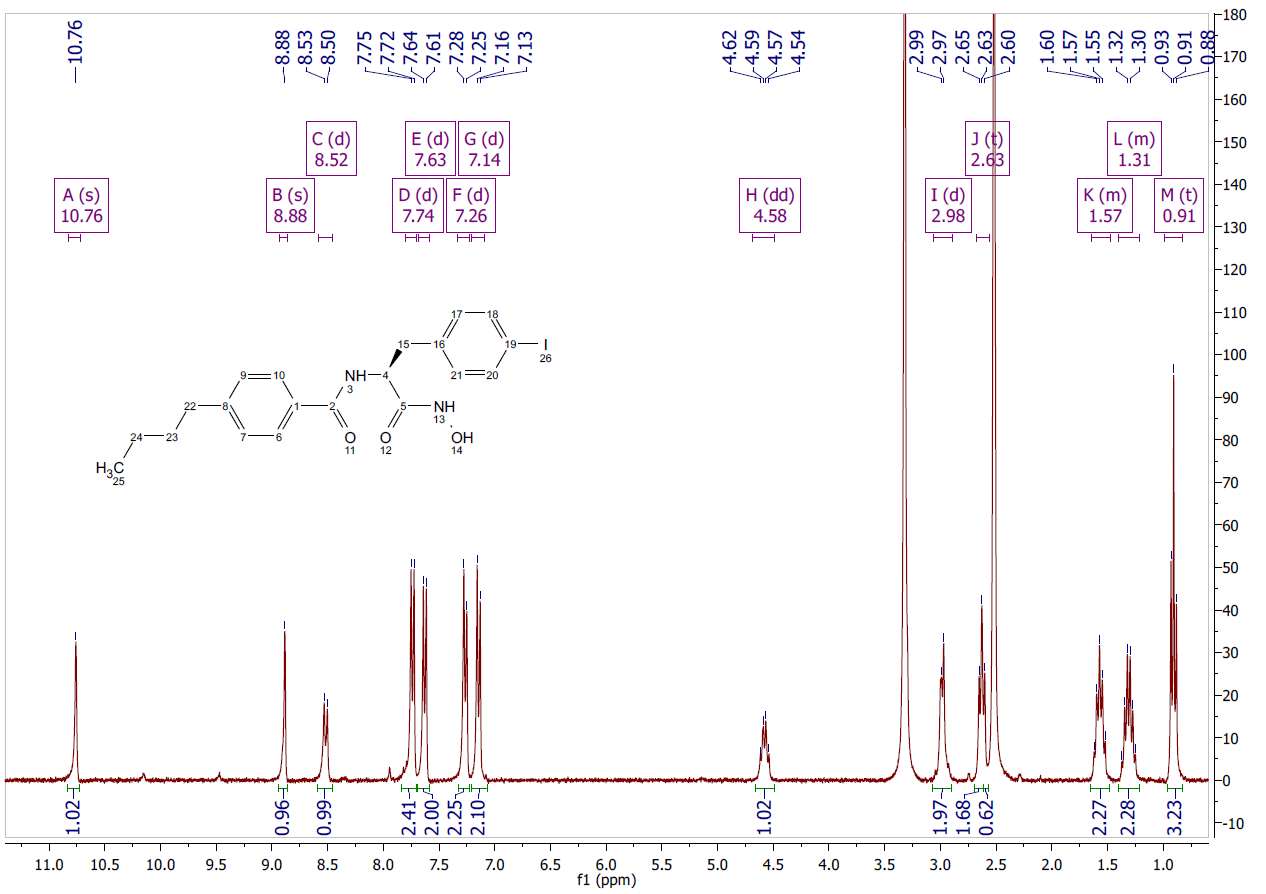


(^1^H-NMR, CDCl3, 300 MHz)

**YSL-125**

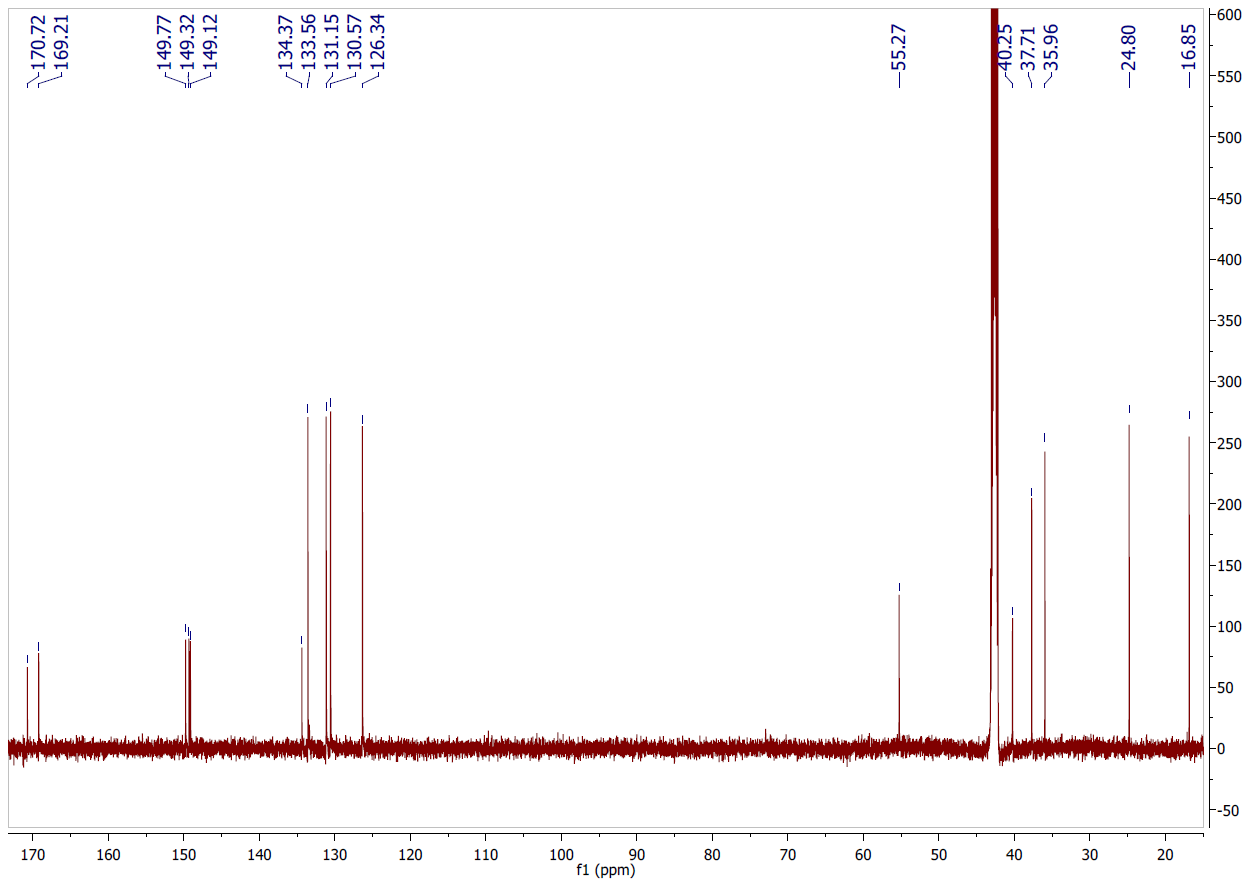


(^13^C-NMR, CDCl3, 300 MHz)


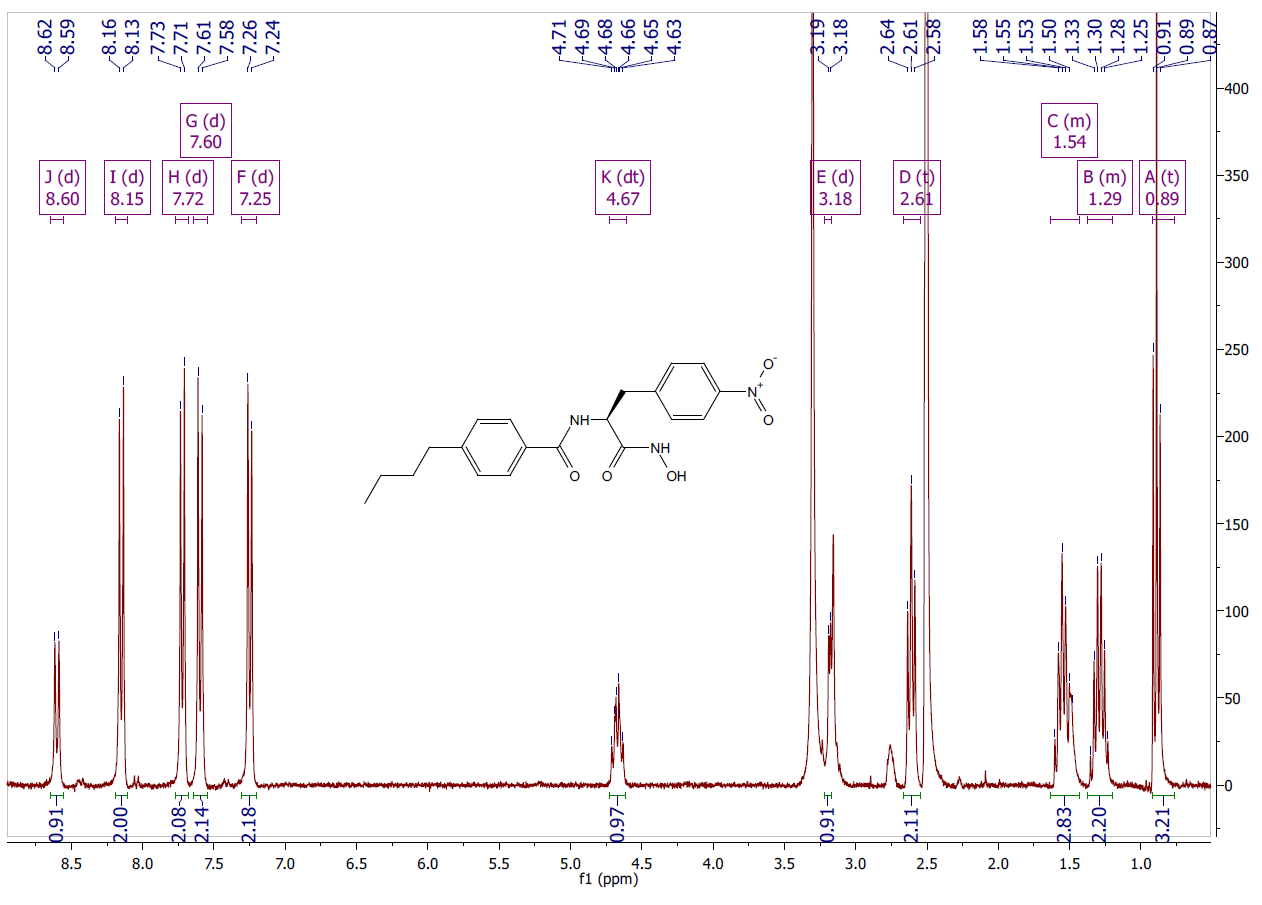


(^1^H-NMR, CDCl3, 300 MHz)

**YSL-129**

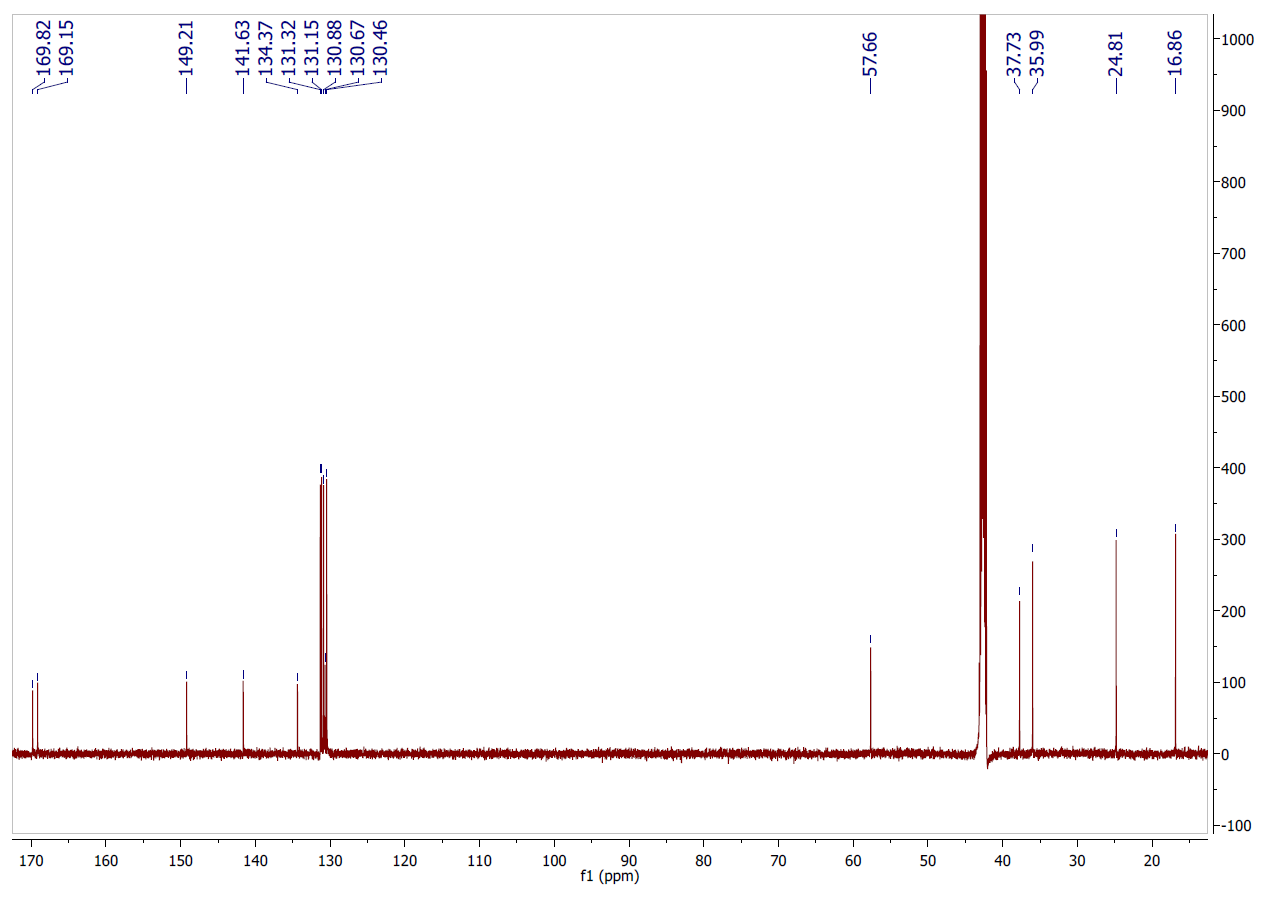


(^13^C-NMR, CDCl3, 300 MHz)


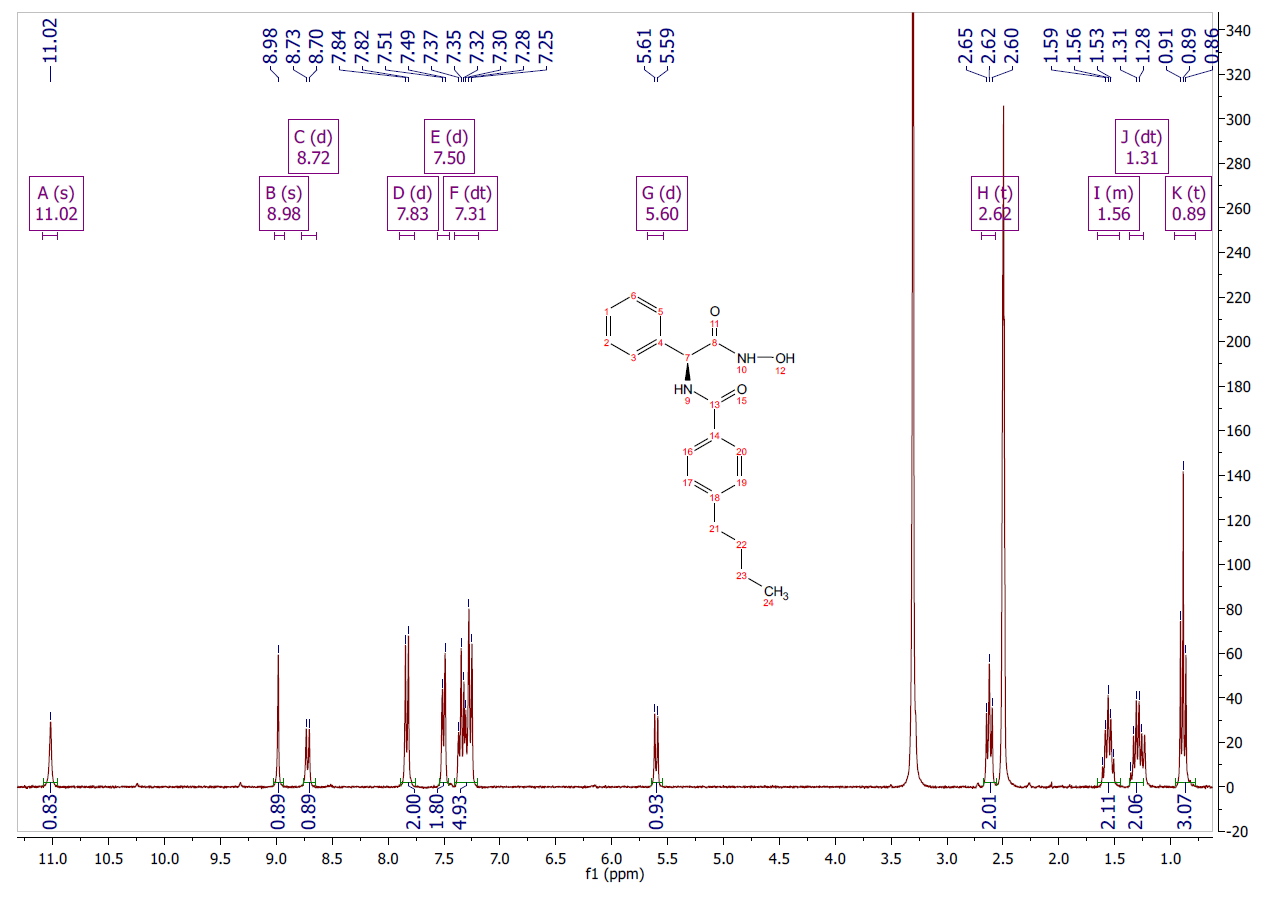


(^1^H-NMR, CDCl3, 300 MHz)

1. Sixto-Lopez, Y.; Bello, M.; Correa-Basurto, J. Insights into structural features of HDAC1 and its selectivity inhibition elucidated by Molecular dynamic simulation and Molecular Docking. In Sección de Estudios de Posgrado e Investigación, Escuela Superior de Medicina, Instituto Politécnico Nacional: 2017.
